# Differentiating migraine, cervicogenic headache and asymptomatic individuals based on physical examination findings: a systematic review and meta-analysis

**DOI:** 10.1186/s12891-021-04595-w

**Published:** 2021-09-03

**Authors:** E. Anarte-Lazo, G. F. Carvalho, A. Schwarz, K. Luedtke, D. Falla

**Affiliations:** 1 Centre of Precision Rehabilitation for Spinal Pain (CPR Spine), College of Life and Environmental Sciences, School of Sport, Exercise and Rehabilitation Sciences, University of Birmingham, Edgbaston, Birmingham, B15 2TT UK; 2 Department of Physiotherapy, Pain and Exercise Research Luebeck (P.E.R.L.), Institute of Health Sciences, University of Luebeck, Luebeck, Germany

**Keywords:** Cervicogenic headache, Migraine, Musculoskeletal disorders, Physical testing

## Abstract

**Background:**

Migraine and cervicogenic headache (CGH) are common headache disorders, although the large overlap of symptoms between them makes differential diagnosis challenging. To strengthen differential diagnosis, physical testing has been used to examine for the presence of musculoskeletal impairments in both conditions. This review aimed to systematically evaluate differences in physical examination findings between people with migraine, CGH and asymptomatic individuals.

**Methods:**

The databases MEDLINE, PubMed, CINAHL, Web of Science, Scopus, EMBASE were searched from inception until January 2020. Risk of bias was assessed with the Downs and Black Scale for non-randomized controlled trials, and with the Quality Assessment of Diagnostic Accuracy Studies tool for diagnostic accuracy studies. When possible, meta-analyses with random effect models was performed.

**Results:**

From 19,682 articles, 62 studies were included in this review and 41 were included in the meta-analyses. The results revealed: a) decreased range of motion [°] (ROM) on the flexion-rotation test (FRT) (17.67, 95%CI:13.69,21.65) and reduced neck flexion strength [N] (23.81, 95%CI:8.78,38.85) in CGH compared to migraine; b) compared to controls, migraineurs exhibit reduced flexion ROM [°] (− 2.85, 95%CI:-5.12,-0.58), lateral flexion ROM [°] (− 2.17, 95% CI:-3.75,-0.59) and FRT [°] (− 8.96, 95%CI:-13.22,-4.69), reduced cervical lordosis angle [°] (− 0.89, 95%CI:-1.72,-0.07), reduced pressure pain thresholds over the cranio-cervical region [kg/cm^2^], reduced neck extension strength [N] (− 11.13, 95%CI:-16.66,-5.6) and increased activity [%] of the trapezius (6.18, 95%CI:2.65,9.71) and anterior scalene muscles (2.87, 95%CI:0.81,4.94) during performance of the cranio-cervical flexion test; c) compared to controls, CGH patients exhibit decreased neck flexion (− 33.70, 95%CI:-47.23,-20.16) and extension (− 55.78, 95%CI:-77.56,-34.00) strength [N].

**Conclusion:**

The FRT and neck flexion strength could support the differential diagnosis of CGH from migraine. Several physical tests were found to differentiate both headache types from asymptomatic individuals. Nevertheless, additional high-quality studies are required to corroborate these findings.

**Study registration:**

Following indications of Prisma-P guidelines, this protocol was registered in PROSPERO on 21/05/2019 with the number CRD42019135269. All amendments performed during the review were registered in PROSPERO, indicating the date and what and why was changed.

**Supplementary Information:**

The online version contains supplementary material available at 10.1186/s12891-021-04595-w.

## Introduction

Headache is one of the most prevalent and disabling conditions resulting in reduced quality of life and lower work productivity [1–3]. Migraine and cervicogenic headache are common primary and secondary headaches, respectively [4]. The overlap of signs and symptoms between these headache types makes the differential diagnosis of headache challenging, leading to an incorrect diagnosis in ~ 50% of cases and subsequently, inappropriate treatment choices [5–7]. Convergence of cervical and trigeminal afferents in the trigeminocervical nucleus, and its bidirectionality, could explain the presence of neck pain in migraineurs and pain perceived as headache in those with cervicogenic headache [8–11].

The diagnostic criteria applied to headache typically adhere to those described by the International Headache Society (IHS) [4] and the criteria proposed by the Cervicogenic Headache International Study Group (CHISG) [12], later re-evaluated by Antonaci et al. [13] In order to strengthen the differential diagnosis of headache, physical testing has been used to determine whether musculoskeletal impairments are present that could be contributing to headache symptoms [14–21]. A previous systematic review analysed the relevance of manual examination in the diagnosis of cervicogenic headache [22] and another compared differences in physical testing between migraine and asymptomatic individuals [23]. However, no systematic review has summarized all the information available regarding the usefulness of different forms of physical testing to differentiate between each headache type and asymptomatic individuals, and especially, between both headache types. Thus, the purpose of this systematic review was to determine whether physical examination can be used to: 1) differentiate between people with cervicogenic headache from those with migraine, 2) distinguish people with migraine from asymptomatic individuals and 3) differentiate people with cervicogenic headache from asymptomatic individuals.

## Methods

The protocol for this systematic review was registered with PROSPERO (CRD42019135269) and published [24]. This review was conducted following the recommendations from the *Cochrane Handbook of Systematic Review of Interventions* [25] where possible and the reporting of the systematic review was conducted in line with the Preferred Reporting Items for Systematic Review and Meta-Analysis guidelines [26, 27].

### Eligibility criteria

The inclusion and exclusion criteria of the studies to be included in the review were defined using the PICOS (P: Population; I: Intervention; C: Comparator; O: Outcome(s); S: Study design) framework [26, 27].

### Inclusion criteria

#### Population

Any study about the physical examination of an adult population (> 18 years old) with migraine or cervicogenic headache, as defined by the IHS [4] or CHISG [12, 13], was included. We also accepted studies where these classification systems were not specifically stated in the inclusion criteria, yet the headache characteristics described were similar. Studies that included other headache types such as tension-type headache, were considered if data on cervicogenic headache or migraine were reported independently. For the studies assessing the diagnostic accuracy in cervicogenic headache, we accepted any diagnosis based on the IHS [4] and CHISG [12, 13] with the exception of diagnostic anaesthetic blocks criteria. In relation to the diagnostic accuracy studies for migraine, diagnosis was based on the IHS criteria for migraine. In addition, this diagnosis was considered acceptable if it did not meet the IHS criteria for other forms of headache. Finally, asymptomatic individuals were defined as those who had no history of described features of cervicogenic headache, migraine without aura, migraine with aura or episodic headache. To be included, the studies had to compare physical examination findings between a) cervicogenic headache and migraine, b) migraine and asymptomatic individuals, and/or c) cervicogenic headache and asymptomatic individuals.

#### Outcome measures of physical testing

Physical examination directed at evaluating the presence or absence of cervical musculoskeletal impairment in people with cervicogenic headache and/or migraine were of interest in this review. As described previously [21], physical examination tests are defined as tests or measures designed to detect a musculoskeletal impairment, performed by a clinician.

We included any study evaluating any physical examination or test designed to evaluate the cervical neuromusculoskeletal system including, but not limited to, range of motion, muscular strength and endurance, reproduction or resolution of symptoms by manual examination, tenderness palpation, proprioceptive measures and balance.

When possible, data on diagnostic accuracy were collected. For diagnostic accuracy, we collected sensitivity, specificity, positive and negative likelihood ratios (LR+ and LR-) and positive and negative predictive values (PPV and NPV). Definition of these concepts can be found in the protocol for this systematic review [24].

#### Study design

Case-control studies were the study design of preference for this review. Cohort or observational study design were also included. If diagnostic tests were performed prior to an intervention, randomized controlled trials were also included. Case studies and previous literature reviews including systematic reviews and meta-analyses were excluded.

### Exclusion criteria

Studies which included people suffering from a serious disease or another diagnosed headache condition not described in the inclusion criteria were not considered. Studies which included individuals with a history of head or neck trauma were also excluded. Studies assessing people with a diagnosed cervical pathology were excluded. In addition, all studies which were not written in English were excluded.

### Data sources and searches

The databases MEDLINE, PubMed, CINAHL, Web of Science, Scopus, EMBASE were searched from inception until January 2020 by two independent reviewers (EA and GC). The design of the search was informed by the PICOS criteria outlined previously, subject specific expertise and the completion of scoping searches. The specific search strategies were developed in consensus by all authors and facilitated by a health science librarian to adapt MESH keywords and natural language terms to the different databases. In addition, a manual search of specific journals was conducted targeting journals where we found potentially eligible studies in our initial scoping search (*Cephalalgia, Headache, The Journal of Headache and Pain, Current Pain and Headache Reports*, *Manual Therapy, Musculoskeletal Science and Practice, Physical Therapy, Journal of Manipulative and Physiological Therapeutics).* The reference lists of studies identified as eligible following the search were hand searched to ensure that no relevant studies were missed. The search strategy included terms referring to the different population studied, and the outcome measures assessed. The following search terms were combined:

- Population

(cervicogenic headache OR migraine disorder) AND

- Physical testing

Physical diagnosis OR physical examination OR manual examination OR physical tests OR cervical musculoskeletal impairments OR endurance OR cranio-cervical flexion OR muscle function OR flexion-rotation OR joint position error OR joint position sense OR tenderness OR tenderness OR trigger point OR joint OR mobility OR range of motion OR pressure pain threshold OR posture OR muscle strength.

### Study selection

Two reviewers (EA and GC) independently screened titles/abstracts against the prespecified inclusion/exclusion criteria. For those that met the inclusion criteria, the full texts were obtained. Moreover, if any uncertainty existed, the full text was retrieved for further clarification. If needed, the authors of the original work were contacted. Screening of full texts was conducted in the same manner using the predefined inclusion/exclusion criteria.

**Fig. 1:**
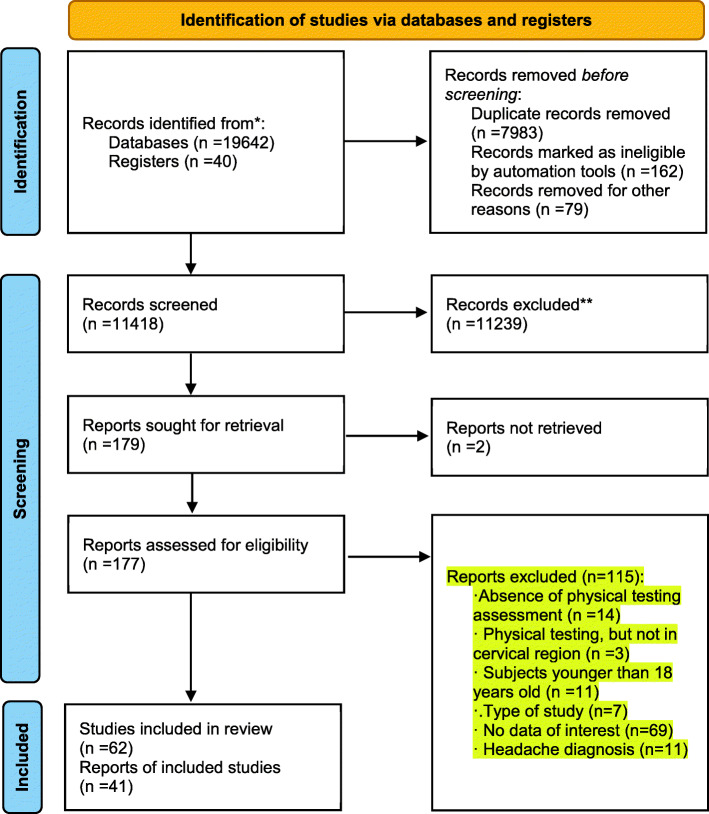
Preferred Reporting Items for Systematic Reviews and Meta-Analyses (PRISMA) flow diagram [26, 27]

Articles were included when eligibility was confirmed by both reviewers. Any disagreement between the two reviewers was first discussed in a consensus meeting between both reviewers, and if no agreement could be made, an independent reviewer (DF) was sought to decide about inclusion/exclusion. Reasons for exclusions can be seen in Fig. 1. Reviewers were not blinded to journal titles or study authors.

### Data extraction and risk of bias

Data extraction was conducted by one reviewer (EA) and then checked by a second reviewer (GC). Author names, year of publication, system used for headache classification, number of participants with each headache condition, number of asymptomatic individuals, age of participants (mean and SD), headache frequency (days/month, mean and SD), headache status during examination, tests used, and test data for headache sufferers and asymptomatic individuals (if reported) were inserted in a predesigned data extraction excel sheet.

For diagnostic accuracy studies, a different data extraction sheet was used including author names, year of publication, clinical test assessment, sensitivity and specificity, LR+/LR- and PPV/NPV [28].

Risk of bias and study quality was assessed using the Downs and Black Scale [29], (except for the diagnostic accuracy studies). This scale is used to evaluate quality of reporting, external and internal validity, and study power through 27 different items. The items that have been proposed to assess intervention efficacy (4, 8, 9, 13, 14, 16, 17, 19, 21–24, and 26) were excluded because the objective of this review was not to assess intervention efficacy. Two reviewers (AS and EA) assessed the risk of bias of each study independently. A quality index (QI) was calculated by dividing the sum of scores by the number of items. A QI of > 75% was considered to indicate a low risk of bias, a QI of 75 to 50% was considered to indicate a moderate risk of bias, and a QI of < 50% was considered to indicate a high risk of bias. For the QI, high internal consistency, good test-retest reliability, and high criterion validity were shown [29]. A Cohen’s Kappa coefficient was calculated to express agreement between reviewers before the consensus meeting. Disagreement between scores were discussed in a consensus meeting. In case of disagreement, a third reviewer (KL) was approached to reach consensus.

The Quality Assessment of Diagnostic Accuracy Studies (QUADAS-II) [30] tool was used to evaluate diagnostic accuracy studies. This tool, differentiates between risk of bias and concerns regarding applicability, classifying risk of bias as “low”, “high” or “unclear”. As with Downs and Black Scale, this tool was used to assess the quality of each study by two reviewers (EA and AS) independently. If there was disagreement, the scoring was discussed with a third reviewer (KL).

### Data synthesis and analysis

A narrative synthesis was conducted for all included studies. If different migraine diagnoses (chronic migraine, migraine with and without aura) were reported, means and standard deviation of subgroup characteristics and test results were combined into one group using Statistics Toolkit STATTOLS [31], as recommended by the *Cochrane Handbook for Systematic Reviews of Interventions* [27]. When physical examination tests were investigated in more than one study, and data was reported on comparable and homogenous scales, the results were pooled in meta-analyses using a random-effects model. Review Manager 5.4 was used to produce analyses and output figures wherever there was sufficient data, which was defined as at least two studies [32]. Heterogeneity among included studies was estimated using the following criteria: I^2^: 0% < I^2^ < 40% was considered as an unimportant heterogeneity; 30% < I^2^ < 60% was considered moderate heterogeneity; 60% < I^2^ < 75% indicated substantial heterogeneity; finally, 75 < I^2^ < 100% indicated considerable heterogeneity [27]. Post-hoc sensitivity analyses were performed excluding those studies with a moderate or high risk of bias, considered when QI was lower than 75%. Differences between patients with migraine, cervicogenic headache and asymptomatic individuals were considered significant if the overall effect had a *P* value of < 0.05 and an I^2^ < 40%.

## Results

### Study selection

Database search and manual searching of specific journals (Fig. 1) resulted in a total of 19,682 articles. After removing duplicates, 11,418 remained. Titles/abstract screening resulted in a total of 177 eligible articles. After full-text assessment, 62 articles were included in this review. Articles were excluded for different reasons: absence of physical examination assessment (*n* = 14), absence of physical examination of the cervical region (*n* = 3), population included < 18 years old (*n* = 11), no data available for extraction (*n* = 69), review article (*n* = 7); non-specific headache diagnosis or headache diagnosis other than migraine or cervicogenic headache which was not of interest for this review (*n* = 11). Excluded articles can be found in Additional file 1.

### Studies characteristics

Of the 62 studies included, 47 assessed only migraineurs, 9 only patients with cervicogenic headache and 6 both headache types. For those studies assessing cervical musculoskeletal impairments in migraine, diagnostic criteria were based on different editions from the International Classification of Headache Disorders (ICHD) (1st edition [17, 33–38], 2nd edition [14, 15, 19, 39–61] and 3rd edition [18, 62–78]). We also included two studies which diagnostic criteria were not based on those from ICHD. One used diagnostic criteria based on a proposed revision of the ICHD [78] and the other one based the diagnosis on similar criteria to this classification [79]. For those studies on cervicogenic headache, diagnostic criteria were based on the International Headache Classification Criteria [33] or on the criteria proposed by the CHISG [14, 16, 17, 19, 34, 38, 80–87].

A total of 2240 patients with migraine, 369 with cervicogenic headache and 1688 asymptomatic individuals were included in this review. Mean ages of each ranged from 22.9 years (SD = 3.5) [14] to 44 years (SD = 5) [69] for migraine, from 24.5 years (SD = 4.8) [81] to 44 years (SD = 11.9) [35] for cervicogenic headache, and from 21.6 years (SD = 0.9) [60] to 44 years (11) [66] for asymptomatic individuals. Only one study included a population with an older mean age [migraineurs, 64 (SD = 3.2); asymptomatic individuals, 65.2 (SD = 3.9)] [80]. Characteristics of the included studies are detailed in Additional file 2.

### Quality assessment

The results for the risk of bias assessment are presented in Additional file 3. The risk of bias of diagnostic accuracy studies are shown in Additional file 4. The agreement between the two assessors on the Downs and Black Scale was considered good, with a Cohen’s kappa coefficient of 0.74 (95% CI 0.67–0.81). During this procedure, only one item showed a higher level of disagreement than the others (item 11). In 29 studies, consensus discussion was needed on at least one item. Thirty-eight studies were rated as low risk of bias [18, 34, 38, 42, 43, 50–55, 59–64, 66–78, 80–83, 85–88], while 17 studies were rated as moderate risk of bias [15, 33, 35, 37–40, 44–49, 56–58, 65] and only two studies were rated as high risk of bias [36, 79].

The agreement was also good when QUADAS-II was applied to assess the quality and risk of bias in diagnostic accuracy studies, with a Cohen kappa coefficient of 0.6 (95% CI 0.26–0.92). Among the 5 studies assessed, consensus discussion was needed for one or more items of two studies. All 5 studies exhibited high risk of bias regarding patient selection and the reference standard assessed. Nonetheless, the index test exhibited a low overall risk of bias.

### Physical examination tests

The physical examinations performed in patients with migraine, cervicogenic headache and asymptomatic individuals are presented in Additional file 2. Among all studies, 23 different tests were identified: balance tests (limits of stability (LOS) [39, 64], postural oscillation [41], and others [35, 39, 41, 56, 64]), pressure pain threshold (PPT) [14, 33, 37, 42–44, 47–51, 55, 59, 62, 67, 76, 77, 80], neck strength and endurance [18, 19, 34, 53, 62, 63, 70, 71, 86], craniocervical flexion test (CCFT) [14, 18, 19, 62, 68, 69], trigger point assessment [18, 35, 40, 45, 48, 60, 73, 86], joint position sense error [14, 18, 34, 66, 81, 84], cervical and cranial tenderness [15, 47, 79], cervical stiffness [58, 60, 65], global cervical range of motion (cervical ROM) [14, 18, 19, 34, 38, 45, 54, 60, 66, 72, 75, 83, 86], posture: craniovertebral angle (CVA) [14, 18, 34, 45, 51, 52, 60, 66, 69], cervical lordosis angle (CLA) [51, 52, 66, 69] and others [14, 35, 51, 69, 71]), passive accessory intervertebral motion test (PAIVMS) [14, 18, 23, 35, 66, 71, 73], passive physiological intervertebral motion test (PPIVMS) [18, 34], skin roll test [34], nerve mechanosensitivity [14, 48, 71], neck muscle electromyography (EMG) measured during the CCFT [14, 62, 68, 69], trigeminocervical reflex (TCR) [36, 57] and other EMG measurements [37, 61, 62, 86], flexion-rotation test (FRT) [14, 16–18, 66, 83–85, 87], muscle length testing [71], muscular tests of shoulder girdle [18], upper cervical quadrant test [18], thoracic spine screening [18], reproduction and resolution of headaches [15, 18], two points discrimination test [74], tone [58] and sensory testing with von Frey hairs [78]. Most measures were not meta-analyzed due to limited data, heterogeneity of assessment and scoring methods; therefore, since some studies assessed the same measurement in different ways or outcomes were reported differently (JPE, muscle activity measured via EMG, among others), not all studies evaluating these measurements could be included in the meta-analysis. Information related to studies not included in the meta-analyses can be found in Additional file 5 (cervicogenic headache versus migraine), 6 (migraine versus asymptomatic individuals) and 7 (cervicogenic headache versus asymptomatic individuals).

To be included in the meta-analyses, the studies were categorized into three groups according to the predefined comparisons: migraine vs cervicogenic headache, migraine vs asymptomatic individuals, and cervicogenic headache vs asymptomatic individuals. Studies included in meta-analyses had to be homogenous and composed by more than one study. In addition, we performed post-hoc sensitivity analyses for those studies with low risk of bias (QI> 75%).

### Cervicogenic headache vs migraine

When cervicogenic headache and migraine were compared, meta-analyses could only be performed for cervical ROM, joint position sense and neck strength. A summary of the meta-analyses and post-hoc sensitivity analyses can be found in Table 1. Forest plots are presented in Additional file 7 for those tests with significant results. Some sub-analyses could not be conducted due to high risk of bias.

- Cervical ROM (°). Reduced range of rotation during FRT in cervicogenic headache patients when compared to migraine patients was shown in the meta-analysis for the FRT (17.67 [95%CI 13.69, 21.65]), which was verified after post-hoc sensitivity analysis, since similar studies were included [16, 17]. However, no differences was found for other movements. The forest plot for meta-analysis and post-hoc sensitivity analysis can be found in Additional file 8.
- Joint position error (°). No significant differences were found for joint position error between people with migraine and cervicogenic headache [14, 19]. Sensitivity analysis could not be performed due to a high risk of bias.
- Neck strength (Newton). Results of the meta-analysis showed a reduction in neck flexion strength (23.81 [95%CI 8.78, 38.85]) in patients with cervicogenic headache, but not for neck extension strength between headache types [19, 34]. Post-hoc sensitivity analysis could not be performed since all studies showed high risk of bias. A forest plot for the meta-analysis can be found in Additional file 8.

**Table 1:** Meta-analyses and post-hoc sensitivity analysis results of physical tests in people with migraine compared to cervicogenic headache

Test	Procedure or location	Analysis	No. of studies	Patients with migraine	Patients with cervicogenic headache	Mean difference (95% CI)	Heterogeneity (I2)	P for overall effect
Cervical ROM	Flexion + extension	Meta-analysis	3	69	79	13.85 [0.24, 27.45]	75%	*P* = 0.05
		Sensitivity analysis (QI> 75%)	0	N/A	N/A	N/A	N/A	N/A
	Rotation (sum of both sides)	Meta-analysis	3	69	79	13.88 [− 0.51, 28.27]	80%	*P* = 0.06
		Sensitivity analysis (QI> 75%)	0	N/A	N/A	N/A	N/A	N/A
	Lateral flexion (sum of both sides)	Meta-analysis	3	69	79	4.56 [− 0.72, 9.85]	0%	*P* = 0.09
		Sensitivity analysis (QI> 75%)	0	N/A	N/A	N/A	N/A	N/A
	FRT (both sides)	Meta-analysis	2	32	43	17.67 [13.69, 21.65]	0%	*P* < 0.00001*
		Sensitivity analysis (QI> 75%)	2	32	43	17.67 [13.69, 21.65]	0%	*P* < 0.00001*
JPE	Extension	Meta-analysis	2	43	49	0.10 [−1.41, 1.60]	0%	*P* = 0.09
		Sensitivity analysis (QI> 75%)	0	N/A	N/A	N/A	N/A	N/A
	Rotation	Meta-analysis	2	43	49	−0.47 [−1.28, 0.34]	0%	*P* = 0.26
		Sensitivity analysis (QI> 75%)	0	N/A	N/A	N/A	N/A	N/A
Strength	Extensors	Meta-analysis	2	38	42	41.55 [10.56, 72.54]	48%	*P* = 0.009*
		Sensitivity analysis (QI> 75%)	0	N/A	N/A	N/A	N/A	N/A
	Flexors	Meta-analysis	2	38	42	23.81 [8.78, 38.85]	0%	*P* = 0.002*
		Sensitivity analysis (QI> 75%)	0	N/A	N/A	N/A	N/A	N/A

*ROM* Range Of Motion, *JPE* joint position error, *QI* Quality Index, *FRT* Flexion-Rotation Test

*Direction of effect for cervicogenic headache

### Migraine vs asymptomatic individuals

We conducted meta-analyses for 7 assessment outcomes: Cervical ROM, joint position error, posture (CVA and CLA), PPT, neck strength, LOS, neck muscle EMG during performance of the CCFT and EMG measures for the TCR. A summary of the meta-analysis and post-hoc sensitivity analysis can be found in Table 2. Forest plots are presented in Additional file 8 for the tests that were significant. Some sub-analyses were not conducted due to high risk of bias.

**Table 2:** Meta-analysis and post-hoc sensitivity analysis of physical testing in patients with migraine compared to asymptomatic individuals

Test	Procedure or location	Analysis	No. of studies	Patients with migraine	Asympto matic indivi duals	Mean difference (95% CI)	Heterogeneity (I2)	P for overall effect
Cervical ROM	Flexion	Meta-analysis	7	258	210	−2.85 [−5.12, − 0.58]	12%	*P* = 0.01*
		Sensitivity analysis (QI> 75%)	5	216	133	−3.57 [−6.28,-0.86]	9%	*P* = 0.01*
	Extension	Meta-analysis	7	258	210	−2.30 [−5.33, 0.73]	44%	*P* = 0.14
		Sensitivity analysis (QI> 75%)	5	216	133	−3.45 [−7.40,0.50]	50%	*P* = 0.09
	Rotation	Meta-analysis	7	258	210	−0.21 [−9.13, 8.71]	96%	*P* = 0.96
		Sensitivity analysis (QI> 75%)	5	216	133	−4.42 [−6.51,-2.33]	0%	*P* < 0.0001*
	Lateral flexion	Meta-analysis	7	258	210	−2.17 [−3.75, −0.59]	0%	*P* = 0.007*
		Sensitivity analysis (QI> 75%)	5	216	133	−1.78 [−3.87,0.32]	13%	*P* = 0.10
	Rotation (sum of both sides)	Meta-analysis	3	69	93	3.54 [−2.23, 9.31]	0%	*P* = 0.23
		Sensitivity analysis (QI> 75%)	0	N/A	N/A	N/A	N/A	N/A
	Lateral flexion (sum of both sides)	Meta-analysis	3	69	93	−0.63 [−6.04, 4.77]	0%	*P* = 0.82
		Sensitivity analysis (QI> 75%)	0	N/A	N/A	N/A	N/A	N/A
	FRT (both sides)	Meta-analysis	2	67	53	−5.86 [−17.12, 5.41]	93%	*P* = 0.31
		Sensitivity analysis (QI> 75%)	2	67	53	−5.86 [− 17.12, 5.41]	93%	*P* = 0.31
	FRT (sum of both sides)	Meta-analysis	2	163	98	−8.96 [−13.22, −4.69]	0%	*P* < 0.0001*
		Sensitivity analysis (QI> 75%)	1	N/A	N/A	N/A	N/A	N/A
	Flexion + extension	Meta-analysis	3	69	93	2.46 [−3.84,8.77]	0%	*P* = 0.44
		Sensitivity analysis (QI> 75%)	0	N/A	N/A	N/A	N/A	N/A
JPE	Extension	Meta-analysis	3	118	104	0.20 [−0.48,0.88]	0%	*P* = 0.57
		Sensitivity analysis (QI> 75%)	0	N/A	N/A	N/A	N/A	N/A
	Rotation (both sides)	Meta-analysis	3	118	104	−0.07 [−0.84,0.7]	25%	*P* = 0.86
		Sensitivity analysis (QI> 75%)	0	N/A	N/A	N/A	N/A	N/A
Posture CVA	Standing	Meta-analysis	5	217	123	−1.11 [−4.10,1.89]	83%	*P* = 0.47
		Sensitivity analysis (QI> 75%)	3	172	78	−0.75 [−3.06,1.57]	62%	*P* = 0.53
	Sitting	Meta-analysis	5	195	102	−2.94 [−5.98,0.09]	75%	*P* = 0.06
		Sensitivity analysis (QI> 75%)	3	175	82	−1.62 [−3.11,-0.13]	62%	*P* = 0.02*
Posture CLA	Standing	Meta-analysis	2	139	45	−0.89 [−1.72,-0.07]	0%	*P* = 0.03*
		Sensitivity analysis (QI> 75%)	2	139	45	−0.89 [−1.72,-0.07]	0%	*P* = 0.03*
	Sitting	Meta-analysis	2	139	45	−0.81 [−1.74, 0.12]	0%	*P* = 0.09
		Sensitivity analysis (QI> 75%)	2	139	45	−0.81 [−1.74, 0.12]	0%	*P* = 0.09
PPT	Upper trapezius	Meta-analysis	5	124	110	−0.61 [−0.92,-0.29]	89%	*P* = 0.0002*
		Sensitivity analysis (QI> 75%)	4	99	85	−0.61 [− 0.99,-0.23]	92%	*P* = 0.002*
	Sternocleidomastoid muscle	Meta-analysis	3	79	65	−0.69 [− 0.98,-0.39	79%	*P* < 0.00001*
		Sensitivity analysis (QI> 75%)	3	79	65	−0.69 [− 0.98,-0.39	79%	*P* < 0.00001*
	Temporalis muscle anterior part	Meta-analysis	4	89	75	−0.67[−0.88,-0.45]	47%	*P* < 0.00001*
		Sensitivity analysis (QI> 75%)	2	49	35	−0.65 [−1.03,-0.27]	63%	*P* = 0.0008*
	Temporalis muscle central part	Meta-analysis	3	64	50	−0.70 [−0.93,-0.47]	37%	*P* < 0.00001*
		Sensitivity analysis (QI> 75%)	2	49	35	−0.64 [−1.02,-0.25]	67%	*P* = 0.001*
	Temporalis muscle posterior part	Meta-analysis	3	64	50	−0.95 [−1.15,-0.75]	0%	*P* < 0.00001*
		Sensitivity analysis (QI> 75%)	2	49	35	−1.02 [−1.25,-0.79]	0%	*P* < 0.00001*
	Average over multiple sites including the splenius capitis and trapezius	Meta-analysis	3	136	102	−0.87 [−1.44,-0.31]	0%	*P* = 0.002*
		Sensitivity analysis (QI> 75%)	3	136	102	−0.87 [−1.44,0.31]	0%	*P* = 0.002*
	GON	Meta-analysis	2	40	40	−3.46 [−9.43,2.52]	99%	*P* = 0.26
		Sensitivity analysis (QI> 75%)	1	N/A	N/A	N/A	N/A	N/A
	Suboccipital	Meta-analysis	2	50	50	−0.80 [−0.85,-0.75]	0%	*P* < 0.00001*
		Sensitivity analysis (QI> 75%)	2	50	50	−0.80 [− 0.85,-0.75]	0%	*P* < 0.00001*
Strength	Extensors	Meta-analysis	6	207	228	−11.13 [−16.66,-5.6]	26%	*P* < 0.0001*
		Sensitivity analysis (QI> 75%)	4	169	154	−10.89 [− 17.64, −4.15]	53%	*P* = 0.002*
	Flexors	Meta-analysis	6	207	228	−4.72 [−8.98,-0.45]	47%	*P* = 0.01*
		Sensitivity analysis (QI> 75%)	4	169	154	−5.00 [−9.25, −0.75]	64%	*P* = 0.03*
	Lateral flexors	Meta-analysis	3	95	101	−12.82 [−24.96,-0.68]	91%	*P* = 0.04*
		Sensitivity analysis (QI> 75%)	3	95	101	−12.82 [−24.96,-0.68]	91%	*P* = 0.04*
LOS	Average reaction time	Meta-analysis	2	130	60	0.00 [−0.58,0.59]	96%	*P* = 0.99
		Sensitivity analysis (QI> 75%)	1	N/A	N/A	N/A	N/A	N/A
EMG CCFT 22 mmHg	Upper trapezius	Meta-analysis	2	120	54	6.18 [2.65,9.71]	0%	*P* = 0.0006**
		Sensitivity analysis (QI> 75%)	2	120	54	6.18 [2.65,9.71]	0%	*P* = 0.0006**
	Splenius	Meta-analysis	2	120	54	3.38 [−0.82,7.58]	79%	*P* = 0.12
		Sensitivity analysis (QI> 75%)	2	120	54	3.38 [−0.82,7.58]	79%	*P* = 0.12
	Sternocleidomastoid muscle	Meta-analysis	2	120	54	0.95 [−2.49,4.40]	22%	*P* = 0.59
		Sensitivity analysis (QI> 75%)	2	120	54	0.95 [−2.49,4.40]	22%	*P* = 0.59
	Anterior scalene	Meta-analysis	2	120	54	2.87 [0.81,4.94]	0%	*P* = 0.006**
		Sensitivity analysis (QI> 75%)	2	120	54	2.87 [0.81,4.94]	0%	*P* = 0.006**
EMG CCFT 30 mmHg	Upper trapezius	Meta-analysis	2	120	54	8.12 [4.33,11.91]	1%	*P* < 0.0001**
		Sensitivity analysis (QI> 75%)	2	120	54	8.12 [4.33,11.91]	1%	*P* < 0.0001**
	Splenius	Meta-analysis	2	120	54	6.11 [−1.52,13.75]	90%	*P* = 0.12
		Sensitivity analysis (QI> 75%)	2	120	54	6.11 [−1.52,13.75]	90%	*P* = 0.12
	Sternocleidomastoid muscle	Meta-analysis	2	120	54	1.12 [−8.92,11.15]	72%	*P* = 0.83
		Sensitivity analysis (QI> 75%)	2	120	54	1.12 [−8.92,11.15]	72%	*P* = 0.83
	Anterior scalene	Meta-analysis	2	120	54	5.99 [0.03,11.94]	75%	*P* = 0.05
		Sensitivity analysis (QI> 75%)	2	120	54	5.99 [0.03,11.94]	75%	*P* = 0.05
Trigeminocervical reflex	Latency, ms	Meta-analysis	2	45	47	0.29 [−2.75,3.34]	56%	*P* = 0.85
		Sensitivity analysis (QI> 75%)	0	N/A	N/A	N/A	N/A	N/A
	Amplitude, mV	Meta-analysis	2	45	47	−0.34 [−0.83,0.15]	94%	*P* = 0.17
		Sensitivity analysis (QI> 75%)	0	N/A	N/A	N/A	N/A	N/A

*ROM* Range Of Motion, *FRT* Flexion Rotation Test, *JPE* Joint Position Error, *PPT* Pressure Pain Threshold, *LOS* Limits Of Stability, *CCFT* craniocervical flexion test, *QI* Quality Index

*Direction of effect found for migraine

**Direction of effect found for asymptomatic individuals

a) Range of motion (°). Results of the meta-analysis showed that range of motion was significantly reduced in patients with migraine for flexion (− 2.85 [95%CI 5.12, − 0.58]), lateral flexion (− 2.17 [95%CI -3.75, − 0.59]) and for the sum of both sides of the FRT (− 8.96 [95%CI 13.22, − 4.69]). These three measurements were considered to have low heterogeneity (I^2^ < 40%) [14, 19, 45, 54, 60, 66, 71, 73, 75]. Post-hoc sensitivity analyses indicated that results for flexion (− 3.57 [95%CI -6.28, − 0.86]) and rotation (− 4.42 [95%CI -6.5, − 2.33]) were significant [14, 54, 60, 66, 71, 73, 75]. Forest plots for meta-analysis and post-hoc sensitivity analysis are presented in Additional file 9.

b) Joint position error (°). Pooling of the data available for joint position error resulted in no significant difference between people with migraine and controls [14, 19, 66].

c) Postural angles (°). Significant differences were found for the reduction of the CLA in patients with migraine when measured in a standing position (− 0.89, [95%CI -1.72, − 0.07]), but not in sitting, both after meta-analysis and post-hoc sensitivity analysis [66, 69]. No between group differences were observed for the CVA regardless of the testing position [14, 34, 45, 51, 60, 66, 69]. Forest plots for meta-analysis and post-hoc sensitivity analysis can be found in Additional file 9.

d) PPT (kg/cm^2)^. The assessment of the posterior region of the temporalis muscle (− 0.95 [95%CI -1.15, − 0.75]) [49, 55, 59], an average of the PPT over multiple sites including the splenius capitis, trapezius, and temporalis (− 0.87 [95%CI 1.44, 0.31]) [42–44] and over the suboccipital muscles (− 0.80 [95%CI -0.85, − 0.75]) [59, 67] revealed a significant and homogenous difference between migraine and controls, both before and after the sensitivity analysis. A meta-analysis revealed a significant difference in the reduction of PPT in patients with migraine over the central region of the temporalis muscle (− 0.95 [95%CI -1.15, − 0.75]) [49, 55, 59]. Assessment of the upper trapezius (midpoint between spinous process of C7 and the acromion) [47, 50, 55, 59, 67], sternocleidomastoid (insertion point next to mastoid process) [55, 59, 67] and the anterior part of temporalis muscle [47, 49, 55, 59] revealed extensive heterogeneity and therefore could not be considered as significant between groups. Finally, PPT over the greater occipital nerve [14, 77] did not show significant differences between migraineurs and controls in the meta-analysis. Forest plots for meta-analysis and post-hoc sensitivity analysis can be found in Additional file 9.

e) Neck strength (Newton). For neck extension strength, meta-analysis [19, 34, 53, 62, 63, 71] confirmed significant difference between migraineurs and controls (− 11–13 [95%CI -16.66, − 5.6]); nonetheless, after post-hoc sensitivity analysis, large heterogeneity was found [53, 63, 71, 88]. In the case of neck flexion [34, 53, 62, 63, 71] and lateral flexion [53, 63, 71], there was high heterogeneity and therefore any difference could not be considered significant. Forest plots for the meta-analysis and post-hoc sensitivity analysis are presented in Additional file 9.

f) Limits of stability, reaction time (seconds). The only balance measure which could be included for meta-analysis (but not for post-hoc sensitivity analysis) was the average reaction time, measured in seconds, and significant differences were not found [39, 64].

g) EMG during performance of the CCFT (normalised EMG %). Among the five different stages of the CCFT, we selected 22 mmHg and 30 mmHg as stages of reference [89]. The activity of the upper trapezius, splenius capitis, sternocleidomastoid and anterior scalene were assessed. Significant differences between groups were only observed for the upper trapezius (6.18 [95%CI 2.65, 9.71]) and anterior scalene (2.87 [95%CI 0.81, 4.94]) (both before and after sensitivity analysis) at 22 mmHg and 30 mmHg [68, 69]. Forest plots for meta-analysis and post-hoc sensitivity analysis are presented in Additional file 9.

i) Trigeminocervical reflex, latency (ms) and amplitude (mV). The meta-analysis revealed no significant difference between migraine and asymptomatic individuals for either the latency or amplitude of the TCR [36, 57]. It was not possible to perform post-hoc sensitivity analysis due to high risk of bias.

### Cervicogenic headache vs asymptomatic individuals

Four physical tests were included: cervical ROM, joint position error, PPT and neck strength. A summary of meta-analysis and post-hoc sensitivity analysis can be found in Table 3. Forest plots can be found in Additional file 10 for the tests which were significant. Some sub-analyses were not conducted due to high risk of bias.

- Cervical ROM (°). Results of the meta-analysis showed that a significant difference was only found for the sum of bilateral lateral flexion (− 5.06 [95%CI, − 10.12, 0.01]) [14, 34, 38]. Due to heterogeneity among studies, some movements assessed (extension, rotation, flexion + extension and FRT) could not be considered to be significant [14, 19, 34, 38, 83–87]. After post-hoc sensitivity analysis, no significant differences were found. Forest plots for the meta-analysis can be found in Additional file 10.
- Joint position error (°). No differences were found between cervicogenic headache and asymptomatic individuals for joint position error following return from neck extension or rotation [14, 19]. Sensitivity analysis could not be performed due to high risk of bias.
- PPT (kg/cm^2)^. Pressure pain threshold was assessed in different studies however, meta-analysis could only be performed when the articular pilar of C2-C3 was evaluated, and no significant differences were found [14, 80]. Sensitivity analysis could not be performed due to high risk of bias. Other studies assessed PPT in the anterior region of temporalis muscle and over the tibialis anterior [80], 22 points distributed over the head including an occipital/frontal PPT ratio [33], over the C2 nerve root, C4 transverse process and greater occipital nerve [14]. No study reported significant difference between cervicogenic headache and asymptomatic individuals.
- Neck strength (Newton). After pooling the available data in relation to neck strength, significant differences were found for both neck flexion (− 33.70 [95%CI 47.23, 20.16]) and extension (− 55.78 [95%CI -77.56, 34.00]) strength [19, 34]. Due to the low-quality index (< 75%), post-hoc sensitivity analysis could not be developed. Forest plots for the meta-analysis can be found in Additional file 10.

**Table 3:** Meta-analysis and post-hoc sensitivity analysis of physical testing in people with cervicogenic headache and asymptomatic individuals

Test	Procedure or location	Analysis	No. of studies	Patients with cervicogenic headache	Asympto matic indivi duals	Mean difference (95% CI)	Heterogeneity (I2)	P for overall effect*
Cervical ROM	Flexion	Meta-analysis	3	86	105	−4.53 [−15.20, 6.13]	95%	*P* = 0.40
		Sensitivity analysis (QI> 75%)	2	68	48	−7.96 [− 19.42,3.49]	94%	*P* = 0.17
	Extension	Meta-analysis	3	86	105	−8.95 [−14.89, −3.01]	75%	*P* = 0.003*
		Sensitivity analysis (QI> 75%)	2	68	48	−6.28 [−13.09,0.53]	74%	*P* = 0.07
	Rotation	Meta-analysis	3	86	105	−8.73 [−17.33, −0.14]	92%	*P* = 0.05
		Sensitivity analysis (QI> 75%)	2	68	48	−4.54 [−12.37, 3.28]	86%	*P* = 0.26
	Lateral flexion	Meta-analysis	3	86	105	−4.39 [−9.48, 0.70]	83%	*P* = 0.09
		Sensitivity analysis (QI> 75%)	2	68	48	−4.71 [−12.53, 3.11]	86%	*P* = 0.26
	Flexion + extension	Meta-analysis	3	79	93	−11.87 [23.14, −0.61]	75%	*P* = 0.04*
		Sensitivity analysis (QI> 75%)	0	N/A	N/A	N/A	N/A	N/A
	Rotation (sum of both sides)	Meta-analysis	3	79	93	−11.46 [−24.77, 1.84]	78%	*P* = 0.09
		Sensitivity analysis (QI> 75%)	0	N/A	N/A	N/A	N/A	N/A
	Lateral flexion (sum of both sides)	Meta-analysis	3	79	93	−5.06 [−10.12, 0.01]	0%	*P* = 0.05
		Sensitivity analysis (QI> 75%)	0	N/A	N/A	N/A	N/A	N/A
	FRT	Meta-analysis	4	124	70	−17.47 [−20.52, − 14.42]	42%	*P* < 0.0001*
		Sensitivity analysis (QI> 75%)	4	124	70	−17.47 [−20.52,-14.42]	42%	*P* < 0.00001*
JPE	Extension	Meta-analysis	2	45	82	0.41 [−0.51, 1.32]	0%	*P* = 0.39
		Sensitivity analysis (QI> 75%)	0	N/A	N/A	N/A	N/A	N/A
	Rotation (both sides)	Meta-analysis	2	45	82	0.13 [−1.33, 1.59]	66%	*P* = 0.86
		Sensitivity analysis (QI> 75%)	0	N/A	N/A	N/A	N/A	N/A
PPT	C2-C3	Meta-analysis	2	45	42	−0.20 [−0.58, 0.18]	0%	*P* = 0.30
		Sensitivity analysis (QI> 75%)	1	N/A	N/A	N/A	N/A	N/A
Strength	Extensors	Meta-analysis	2	42	74	−55.78 [−77.56, −34.00]	0%	*P* < 0.00001*
		Sensitivity analysis (QI> 75%)	0	N/A	N/A	N/A	N/A	N/A
	Flexors	Meta-analysis	2	42	74	−33.70 [−47.23, −20.16]	0%	*P* < 0.00001*
		Sensitivity analysis (QI> 75%)	0	N/A	N/A	N/A	N/A	N/A

*ROM* Range Of Motion, *FRT* Flexion-Rotation Test, *JPE* Joint Position Error, *PPT* Pressure Pain Threshold, *QI* Quality Index

*Direction of effect found for cervicogenic headache

### Diagnostic accuracy studies

Due to the large heterogeneity between studies, it was not possible to develop meta-analyses for the diagnostic accuracy studies. Therefore, we present a narrative synthesis of the results. In addition, a summary can be found in Table 4.

**Table 4:** Diagnostic accuracy for clinical tests for cervicogenic headache and migraine

Study	Clinical Test Assessment	Sensitivity/specificity	LR+/LR-	PPV/NPV
Hall, 2008 [84]	FRT experienced examiners	90–90/90–85	9–6/0.12–0.11	0.9–0.85/0.9–0.89
	FRT inexperienced examiners	83–83/92–83	10–5/0.2–0.18	0.9–0.83/0.84–0.83
Hall, 2010 [16, 85]	FRT	70/70	2.33/0.43	0.54/0.82
Jull, 2007 [19]	Group of tests: cervical ROM, palpation C0-C3, CCFT	100/94.4	−/−	−/−
Ogince, 2007 [17]	FRT	91.3–91.3/91.4–88.6	10.65–7.99/0.095–0.098	0.87–0.84/0.94–0.94
Zito, 2006 [14]	PAIVM C0-C1 CGH	70.4/76	2.93/0.39	0.76/0.70
	PAIVM C1-C2 CGH	72.2/80	3.61/0.35	0.79/0.72
	PAIVM C2-C3 CGH	48/92	6/0.56	0.86/0.62
	PAIVM C3-C4 CGH	20.3/96	5.1/0.83	0.84/0.53
	PAIVM C0-C1 M	28/76	1.16/0.95	0.54/0.25
	PAIVM C1-C2 M	28/80	1.4/0.9	0.58/0.22
	PAIVM C2-C3 M	20/92	2.5/0.87	0.71/0.1
	PAIVM C3-C4 M	16/96	4/0.87	0.8/0.05

*LR+/LR-* positive likelihood ratio/negative likelihood ratio, *PPV/NPV* positive predictive value/negative predictive value, *CGH* cervicogenic headache, *M* Migraine, *FRT* Flexion-Rotation Test, *ROM* Range Of Motion, *PAIVM* Passive Accessory Intervertebral Movement, *CCFT* Cranio-Cervical Flexion Test

Five studies on diagnostic accuracy were included in our review, and all assessed diagnostic accuracy of physical tests in the diagnosis of cervicogenic headache, except one which also assessed diagnostic accuracy of testing for migraine. The most studied test was the FRT, which was considered in three studies. The sensitivity for cervicogenic headache ranged from 70 to 91.3%, specificity from 70 to 92%, LR+ from 2.33 to 10, LR- from 0.09 to 0.43, PPV from 0.54 to 0.9 and NPV from 0.82 to 0.9 [16, 17, 84]. Another study evaluated the sensitivity/specificity of a battery of tests applied together: cervical ROM, palpation C0-C3 and CCFT, and found values of 100/94.4 respectively [19]. Finally, another study assessed diagnostic accuracy of PAIVM from C0-C1 to C3-C4 joints, both in cervicogenic headache and migraine. In the cervicogenic headache group, sensitivity/specificity ranged from 20.3 to 72.2/76 to 96, LR+/LR- from 2.93 to 6/0.35 to 0.83, and PPV/NPV from 0.76 to 0.86/0.53 to 0.72; in the migraine group, sensitivity/specificity ranged from 16 to 28/76 to 96, LR+/LR- from 1.16 to 5.1/0.87 to 0.93, and PPV/NPV from 0.54 to 0.8/0.05 to 0.25 [14].

## Discussion

The IHS classification offers a guide to identify different headache disorders [4]. However, this classification is based on clinical presentations that can overlap among different headache types, and therefore the diagnosis of different headache types can be challenging [7]. A recent article reported a large symptomatic overlap between migraine and cervicogenic headache in relation to the location and extent of pain confirming that the consideration of symptoms alone is a major limitation for differential diagnosis [89]. The large symptomatic overlap between cervicogenic headache and migraine highlights the relevance of physical testing to strengthen differential diagnosis which would help to inform the appropriate treatment strategy.

We conducted the most comprehensive review and meta-analyses to date, analysing differences in physical impairments in people with migraine versus cervicogenic headache, and both headache conditions compared to asymptomatic individuals. Sixty-two studies assessing cervical musculoskeletal impairments were included in the systematic review and 41 of these were included in the meta-analysis.

### Differentiating migraine from cervicogenic headache based on physical examination findings

Meta-analyses of the results of these studies revealed a reduction of the range of rotation during the FRT and neck flexion strength in patients with cervicogenic headache compared to those with migraine. Overall, our findings suggest that these two physical tests could support the differentiation of cervicogenic headache from migraine; people with cervicogenic headache are more likely to present with reduced range of motion during the FRT and reduced neck flexor strength. Compared to previous publications [22, 23], our review identified additional musculoskeletal impairments in patients with cervicogenic headache, identified on physical tests (e.g. strength) other than manual therapy, which was the main focus of a previous systematic review which exclusively studied differences between headache types using manual therapy assessment [22].

### Differentiating migraine or cervicogenic headache from asymptomatic individuals based on physical examination findings

A further finding of the current review was the identification of tests of cervical musculoskeletal impairment which could be used to support the differentiation of people with headache (either cervicogenic headache or migraine) compared to asymptomatic individuals. Patients with migraine, compared to asymptomatic individuals, present with reduced cervical ROM (flexion, bilateral flexion and the sum of bilateral rotation on the FRT, but not for the mean of rotation to both sides of FRT), a reduced cervical lordosis angle when measured in a standing position, greater pressure pain sensitivity when PPT was assessed in central and posterior regions of the temporalis muscle, suboccipital muscles and an average measure of PPT when tested over multiple sites including the splenius capitis and trapezius, reduced neck extension strength, and increased activity of the trapezius and anterior scalene muscles during performance of the CCFT. The pooled data showed that the patients with cervicogenic headache presented with reduced range of bilateral lateral flexion, and reduced neck flexion and extension strength compared to asymptomatic individuals.

The current review included additional studies [39–41, 46, 51, 56, 57, 61–64, 67, 72, 74, 75, 77, 79, 85, 88] which were not evaluated in a recent systematic review comparing musculoskeletal findings between migraine and asymptomatic individuals [23]. This likely explains some discrepancy in findings between the current and previous review [23]. Specifically, increased activity in superficial flexors during the CCFT measured via electromyography, reduced cervical lordosis angle, and reduced PPT in upper trapezius was not assessed or reported in the previous review.

Interestingly, we identified more differences in musculoskeletal impairment when people with migraine were compared with asymptomatic individuals than when people with cervicogenic headache were compared to asymptomatic individuals. This however does not imply that migraine is a headache with more musculoskeletal impairments. As it has been argued before, positive findings in physical testing must be interpreted with caution and as part of a clinical reasoning process, since they may reflect increased sensitivity to nociception, caused by a sensitized trigemino-cervical nucleus [90].

Another interesting observation was that differences in FRT were identified between migraine patients and asymptomatic individuals when rotation range of motion was measured as the sum of both sides [14, 18], but not as the mean of both sides [17, 75]. This may be related to the fact that one study assessing FRT as the mean of both sides specifically excluded patients with migraine that reported neck pain [17].

### Diagnostic accuracy of physical tests for the diagnosis of cervicogenic headache or migraine

In this systematic review, we also collated information on the diagnostic accuracy of physical tests for the diagnosis of cervicogenic headache or migraine. Due to the large heterogeneity between studies, it was not possible to develop meta-analyses for the diagnostic accuracy studies. Our review of studies highlights previous findings that a positive FRT, but also a pattern of palpable painful upper cervical joint dysfunction associated with a restriction of ROM (extension) and with muscle impairment (measured through CCFT) appear to be the best clinical tests in terms of sensitivity and specificity for the detection of cervicogenic headache [17, 19, 84]. In addition, our findings show that C1-C2 was the most symptomatic segment, as reported previously [21]. Nonetheless, we should interpret these findings with caution, since different comparisons were assessed in these studies, and the inclusion criteria were not homogenous.

### Clinical considerations

Identifying the existence of musculoskeletal dysfunction in either migraine or cervicogenic headache is relevant since physical therapy interventions implemented to treat these impairments could improve clinical outcomes. For instance, our results suggest that the range of rotation during the FRT and neck flexion strength could support the differentiation of cervicogenic headache from migraine and, given the existence of these musculoskeletal impairments, they may be relevant to target during the management of people with cervicogenic headache. However, it should be noted that reduced rotation on the FRT (sum of bilateral rotation) was also one of the tests which was different between people with migraine and asymptomatic individuals. Thus, it is evident that further research is needed to determine clinically relevant cut off scores for “impaired” FRT in people with cervicogenic headache versus migraine if this test is to be used to strengthen differential diagnosis between these headache types. Due to the overlap in clinical findings both in migraine and cervicogenic headache when compared to controls, it is evident that physical testing alone could not be used to distinguish between both conditions. Instead, physical assessment findings should be integrated with subjective reports within a clinical reasoning framework to reduce any uncertainty.

### Study limitations

Despite the methodological strengths of the meta-analysis, the results of this review are limited due to the heterogeneity among studies, considering the different physical examination procedures and reporting of data. As a result, not all studies could be included in the meta-analysis, and among those studies included, not all measures were added to the quantitative synthesis. In addition, due to the heterogeneity, mean differences were analysed using random-effects model. Moreover, we combined different migraine conditions (e.g. migraine with/without aura, chronic/episodic migraine) for data analysis and it remains unknown whether our findings would differ depending on the specific subtype of migraine. A further potential consideration is that migraine is a cyclic disorder [91] and thus physical assessment findings may vary across this cycle unlike cervicogenic headache findings which are more likely to be stable over prolonged periods of time. Assessment of the stability of physical findings might prove to be one of the main differences between headache types. In addition, neck pain cannot be considered as a cause or a consequence of migraine due to the wide variety of clinical presentations [92].

It should also be recognised that we only considered physical tests performed in a physiotherapy examination, but other procedures such as other forms of quantitative sensory testing or endogenous pain modulation were not considered. These measurements could provide more accurate information in terms of pain mechanisms, although alterations in nociceptive processing may be modality, measure and location specific [93]. Finally, we did not perform a search of grey literature although this was originally considered, and non-English studies were excluded. Therefore, relevant data could be missing.

## Conclusion

In conclusion, we identified two measures of cervical musculoskeletal impairment that could help to differentiate between cervicogenic headache and migraine: the FRT and neck flexion strength. Nevertheless, reduced rotation on the sum of bilateral rotation in the FRT was also one of the tests that differentiate people with migraine to asymptomatic individuals. Given the presence of a wide range of musculoskeletal impairments in both headache types, physical findings alone cannot provide a definitive diagnosis of cervicogenic headache versus migraine. Further high-quality studies are required before definitive conclusions can be made about the role of physical testing in the differentiation of cervicogenic headache and migraine.

## Supplementary Information

**Additional file 1.** Excluded studies.

**Additional file 2.** Studies characteristics.

**Additional file 3.** Risk of bias assessment with a modified version of Downs and Black Scale.

**Additional file 4.** Quality assessment with QUADAS-II.

**Additional file 5.** Outcomes not included in the meta-analysis comparing cervicogenic headache versus migraine.

**Additional file 6.** Outcomes not included in the meta-analysis comparing migraine and asymptomatic individuals.

**Additional file 7.** Outcomes not included in the meta-analysis comparing cervicogenic headache versus asymptomatic individuals.

**Additional file 8.** Forest plots for meta-analysis and post-hoc sensitivity analysis concerning cervicogenic headache versus migraine comparison.

**Additional file 9.** Forest plots for meta-analysis and post-hoc sensitivity analysis concerning migraine and asymptomatic individuals comparison.

**Additional file 10.** Forest plots for meta-analysis and post-hoc sensitivity analysis concerning cervicogenic headache and asymptomatic individuals comparison.

## Data Availability

All data generated or analyzed data in study are included in this article.
