# Supplementary material for: Differentiating migraine, cervicogenic headache and asymptomatic individuals based on physical examination findings: a systematic review and meta-analysis
Source: BMC Musculoskelet Disord. 2021 Sep 3;22:755. doi: 10.1186/s12891-021-04595-w (PMC8417979; doi:10.1186/s12891-021-04595-w)
Supplement: Supplementary file 1 — Additional file 1. Excluded studies. [file 12891_2021_4595_MOESM1_ESM.docx]

**Additional file 1.** Excluded studies

| Article | Reference |
| --- | --- |
| Abaspour, 2015 | Abaspour O, Javanshir K, Amiri M, Karimlou M. Relationship between cross sectional area of Longus Colli muscle and pain laterality in patients with cervicogenic headache. J Back Musculoskelet Rehabil. 2015;28(2):393-9. |
| Abaspour, 2019 | Abaspour O, Akbari M, Rezasoltani A, Ahmadi A. Relationship between thickness of deep neck muscles synergy and painful side in patients with cervicogenic headache. Cranio. 2019 Oct 1:1-7. |
| Amiri, 2007 | Amiri M, Jull G, Bullock-Saxton J, Darnell R, Lander C. Cervical musculoskeletal impairment in frequent intermittent headache. Part 2: subjects with concurrent headache types. Cephalalgia. 2007 Aug;27(8):891-8. |
| Anttila, 2002 | Anttila P, Metsähonkala L, Mikkelsson M, Aromaa M, Kautiainen H, Salminen J, Viander S, Jäppilä E, Sillanpää M. Muscle tenderness in pericranial and neck-shoulder region in children with headache. A controlled study. Cephalalgia. 2002 Jun;22(5):340-4. |
| Ashina, 2014 | Ashina S, Bendtsen L, Lyngberg AC, Lipton RB, Hajiyeva N, Jensen R. Prevalence of neck pain in migraine and tension-type headache: a population study. Cephalalgia. 2015 Mar;35(3):211-9. |
| Ashina, 2018 | Ashina S, Lipton RB, Bendtsen L, Hajiyeva N, Buse DC, Lyngberg AC, Jensen R. Increased pain sensitivity in migraine and tension-type headache coexistent with low back pain: A cross-sectional population study. Eur J Pain. 2018 May;22(5):904-914. |
| Bakhtadze, 2011 | M A Bakhtadze, J Patijn, V N Galaguza, D A Bolotov & A A Popov (2011) Inter-examiner reproducibility of the segmental motion palpation springing test for side bending at level C2–C3, International Musculoskeletal Medicine,33:1, 8-14 |
| Bodes-pardo, 2013 | Bodes-Pardo G, Pecos-Martín D, Gallego-Izquierdo T, Salom-Moreno J, Fernández-de-Las-Peñas C, Ortega-Santiago R. Manual treatment for cervicogenic headache and active trigger point in the sternocleidomastoid muscle: a pilot randomized clinical trial. J Manipulative Physiol Ther. 2013 Sep;36(7):403-11 |
| Borg-stein, 2002 | Borg-Stein J. Cervical myofascial pain and headache. Curr Pain Headache Rep. 2002 Aug;6(4):324-30. |
| Bragatto, 2018 | Bragatto MM, Bevilaqua-Grossi D, Benatto MT, Lodovichi SS, Pinheiro CF, Carvalho GF, Dach F, Fernández-de-Las-Peñas C, Florencio LL. Is the presence of neck pain associated with more severe clinical presentation in patients with migraine? A cross-sectional study. Cephalalgia. 2019 Oct;39(12):1500-1508. |
| Budelmann, 2013 | Budelmann K, von Piekartz H, Hall T. Is there a difference in head posture and cervical spine movement in children with and without pediatric headache? Eur J Pediatr. 2013 Oct;172(10):1349-56. |
| Calhoun, 2010 | Calhoun AH, Ford S, Millen C, Finkel AG, Truong Y, Nie Y. The prevalence of neck pain in migraine. Headache. 2010 Sep;50(8):1273-7. |
| Carvalho, 2014 | Carvalho GF, Chaves TC, Gonçalves MC, Florencio LL, Braz CA, Dach F, Fernández de Las Peñas C, Bevilaqua-Grossi D. Comparison between neck pain disability and cervical range of motion in patients with episodic and chronic migraine: a cross-sectional study. J Manipulative Physiol Ther. 2014 Nov-Dec;37(9):641-6. |
| Chatchawan, 2014 | Chatchawan U, Eungpinichpong W, Sooktho S, Tiamkao S, Yamauchi J. Effects of Thai traditional massage on pressure pain threshold and headache intensity in patients with chronic tension-type and migraine headaches. J Altern Complement Med. 2014 Jun;20(6):486-92. |
| Christian, 2017 | Christian, Neeti. Comparative Study to Find the Effect of Mulligan's Snag Technique (C1-C2) Versus Maitland's Technique (C1-C2) in Cervicogenic Headache among Information Technology Professionals. 2017. International Journal of Physiotherapy. 4. |
| Chua, 2011 | Chua NH, van Suijlekom HA, Vissers KC, Arendt-Nielsen L, Wilder-Smith OH. Differences in sensory processing between chronic cervical zygapophysial joint pain patients with and without cervicogenic headache. Cephalalgia. 2011 Jun;31(8):953-63. |
| Cuthbert, 2018 | Cuthbert S, Blum C, Rosner A. The Association of Manual Muscle Tests and Treatment Outcomes With Headache and Cranial Dysfunctions: A Retrospective Case Series Report. Altern Ther Health Med. 2018 Nov;24(6):8-21. |
| Detommaso, 2014 | de Tommaso M, Ambrosini A, Brighina F, Coppola G, Perrotta A, Pierelli F, Sandrini G, Valeriani M, Marinazzo D, Stramaglia S, Schoenen J. Altered processing of sensory stimuli in patients with migraine. Nat Rev Neurol. 2014 Mar;10(3):144-55. |
| Do, 2018 | Do TP, Heldarskard GF, Kolding LT, Hvedstrup J, Schytz HW. Myofascial trigger points in migraine and tension-type headache. J Headache Pain. 2018 Sep 10;19(1):84. |
| Dong, 2019 | Dong Y, Guo T, Xu L, Wang C, Wen G, Zhao Z, Duan L, Zou M, Xiang Y, Wang S. Cervicogenic headache treated by acupuncture based on jin theory: study protocol for a randomized controlled trial. Trials. 2019 Jul 10;20(1):418. |
| Dunning, 2013 | Dunning JR, Butts R, Mourad F, Young I, Fernandez-de-Las Peñas C, Hagins M, Stanislawski T, Donley J, Buck D, Hooks TR, Cleland JA. Upper cervical and upper thoracic manipulation versus mobilization and exercise in patients with cervicogenic headache: a multi-center randomized clinical trial. BMC Musculoskelet Disord. 2016 Feb 6;17:64. |
| Farmer, 2014 | Farmer PK, Snodgrass SJ, Buxton AJ, Rivett DA. An investigation of cervical spinal posture in cervicogenic headache. Phys Ther. 2015 Feb;95(2):212-22. |
| Fernandez-de-las-Peñas, 2007 | Fernández-de-Las-Peñas C, Simons D, Cuadrado ML, Pareja J. The role of myofascial trigger points in musculoskeletal pain syndromes of the head and neck. Curr Pain Headache Rep. 2007 Oct;11(5):365-72. |
| Fernandez-de-las-Peñas, 2007b | César Fernández-de-las-Peñas, María Luz Cuadrado, Francisco J. Barriga & Juan A. Pareja (2009) Active Muscle Trigger Points as Sign of Sensitization in Chronic Primary Headaches, Journal of Musculoskeletal Pain, 17:2, 155-161, |
| Fernandez-de-las-Peñas, 2017 | Fernández-de-Las-Peñas C, Falla D, Palacios-Ceña M, Fuensalida-Novo S, Arias-Buría JL, Schneebeli A, Arend-Nielsen L, Barbero M. Perceived Pain Extent is Not Associated With Widespread Pressure Pain Sensitivity, Clinical Features, Related Disability, Anxiety, or Depression in Women With Episodic Migraine. Clin J Pain. 2018 Mar;34(3):217-221. |
| Ferracini, 2014 | Ferracini GN, Stuginsk-Barbosa J, Dach F, Speciali JG. A comparison pressure pain threshold in pericranial and extracephalic regions in children with migraine. Pain Med. 2014 Apr;15(4):702-9. |
| Ferracini, 2016b | Ferracini GN, Florencio LL, Dach F, Chaves TC, Palacios-Ceña M, Fernández-de-Las-Peñas C, Bevilaqua-Grossi D, Speciali JG. Myofascial Trigger Points and Migraine-related Disability in Women With Episodic and Chronic Migraine. Clin J Pain. 2017 Feb;33(2):109-115. |
| Ferracini, 2016c | Ferracini GN, Chaves TC, Dach F, Bevilaqua-Grossi D, Fernández-de-Las-Peñas C, Speciali JG. Relationship Between Active Trigger Points and Head/Neck Posture in Patients with Migraine. Am J Phys Med Rehabil. 2016 Nov;95(11):831-839. |
| Ferracini, 2017b | Ferracini GN, Chaves TC, Dach F, Bevilaqua-Grossi D, Fernández-de-Las-Peñas C, Speciali JG. Analysis of the cranio-cervical curvatures in subjects with migraine with and without neck pain. Physiotherapy. 2017 Dec;103(4):392-399. |
| Florencio, 2016b | Florencio LL, Ferracini GN, Chaves TC, Palacios-Ceña M, Ordás-Bandera C, Speciali JG, Falla D, Grossi DB, Fernández-de-Las-Peñas C. Active Trigger Points in the Cervical Musculature Determine the Altered Activation of Superficial Neck and Extensor Muscles in Women With Migraine. Clin J Pain. 2017 Mar;33(3):238-245. |
| Florencio, 2017 | Florencio LL, de Oliveira AS, Carvalho GF, Dach F, Bigal ME, Fernández-de-Las-Peñas C, Bevilaqua-Grossi D. Association Between Severity of Temporomandibular Disorders and the Frequency of Headache Attacks in Women With Migraine: A Cross-Sectional Study. J Manipulative Physiol Ther. 2017 May;40(4):250-254. |
| Gandolfi, 2018 | Gandolfi M, Geroin C, Valè N, Marchioretto F, Turrina A, Dimitrova E, Tamburin S, Serina A, Castellazzi P, Meschieri A, Ricard F, Saltuari L, Picelli A, Smania N. Does myofascial and trigger point treatment reduce pain and analgesic intake in patients undergoing onabotulinumtoxinA injection due to chronic intractable migraine? Eur J Phys Rehabil Med. 2018 Feb;54(1):1-12. |
| Garrigos-pedron, 2018 | Garrigós-Pedrón M, La Touche R, Navarro-Desentre P, Gracia-Naya M, Segura-Ortí E. Effects of a Physical Therapy Protocol in Patients with Chronic Migraine and Temporomandibular Disorders: A Randomized, Single-Blinded, Clinical Trial. J Oral Facial Pain Headache. 2018 Spring;32(2):137-150. |
| Garrigos-pedron, 2019 | Garrigós-Pedrón M, La Touche R, Navarro-Desentre P, Gracia-Naya M, Segura-Ortí E. Widespread mechanical pain hypersensitivity in patients with chronic migraine and temporomandibular disorders: relationship and correlation between psychological and sensorimotor variables. Acta Odontol Scand. 2019 Apr;77(3):224-231. |
| Ghanbari, 2015 | Ghanbari A, Askarzadeh S, Petramfar P, Mohamadi M. Migraine responds better to a combination of medical therapy and trigger point management than routine medical therapy alone. NeuroRehabilitation. 2015;37(1):157-63. |
| Giamberardino, 2007 | Giamberardino MA, Tafuri E, Savini A, Fabrizio A, Affaitati G, Lerza R, Di Ianni L, Lapenna D, Mezzetti A. Contribution of myofascial trigger points to migraine symptoms. J Pain. 2007 Nov;8(11):869-78. |
| Gobel, 1992 | Göbel H, Weigle L, Kropp P, Soyka D. Pain sensitivity and pain reactivity of pericranial muscles in migraine and tension-type headache. Cephalalgia. 1992 Jun;12(3):142-51. |
| Gonçalves, 2013 | Gonçalves MC, Florencio LL, Chaves TC, Speciali JG, Bigal ME, Bevilaqua-Grossi D. Do women with migraine have higher prevalence of temporomandibular disorders? Braz J Phys Ther. 2013 Jan-Feb;17(1):64-8. |
| Gonçalves, 2014 | Gonçalves MC, Chaves TC, Florencio LL, Carvalho GF, Dach F, Fernández-De-Las-Penãs C, Bevilaqua-Grossi D. Is pressure pain sensitivity over the cervical musculature associated with neck disability in individuals with migraine? J Bodyw Mov Ther. 2015 Jan;19(1):67-71. |
| Grossi, 2015 | Bevilaqua-Grossi D, Gonçalves MC, Carvalho GF, Florencio LL, Dach F, Speciali JG, Bigal ME, Chaves TC. Additional Effects of a Physical Therapy Protocol on Headache Frequency, Pressure Pain Threshold, and Improvement Perception in Patients With Migraine and Associated Neck Pain: A Randomized Controlled Trial. Arch Phys Med Rehabil. 2016 Jun;97(6):866-74. |
| Guerrero-Peral, 2017 | Guerrero-Peral ÁL, Ruíz M, Barón J, Palacios-Ceña M, Arendt-Nielsen L, Fernández-de-Las-Peñas C. Roller pressure algometry as a new tool for assessing dynamic pressure sensitivity in migraine. Cephalalgia. 2018 Jun;38(7):1257-1266. |
| Haas, 2010 | Haas M, Spegman A, Peterson D, Aickin M, Vavrek D. Dose response and efficacy of spinal manipulation for chronic cervicogenic headache: a pilot randomized controlled trial. Spine J. 2010 Feb;10(2):117-28. |
| Haas, 2018 | Haas M, Bronfort G, Evans R, Schulz C, Vavrek D, Takaki L, Hanson L, Leininger B, Neradilek MB. Dose-response and efficacy of spinal manipulation for care of cervicogenic headache: a dual-center randomized controlled trial. Spine J. 2018 Oct;18(10):1741-1754. |
| Hall, 2007 | Hall T, Chan HT, Christensen L, Odenthal B, Wells C, Robinson K. Efficacy of a C1-C2 self-sustained natural apophyseal glide (SNAG) in the management of cervicogenic headache. J Orthop Sports Phys Ther. 2007 Mar;37(3):100-7. |
| Hall, 2008b | Hall, T., Briffa, K., & Hopper, D. (2008). Clinical evaluation of cervicogenic headache: a clinical perspective. The Journal of manual & manipulative therapy, 16(2), 73–80. |
| Hall, 2010c | Hall T, Briffa K, Hopper D, Robinson K. Long-term stability and minimal detectable change of the cervical flexion-rotation test. J Orthop Sports Phys Ther. 2010 Apr;40(4):225-9. |
| Hall, 2010d | Hall T, Briffa K, Hopper D, Robinson K. Reliability of manual examination and frequency of symptomatic cervical motion segment dysfunction in cervicogenic headache. Man Ther. 2010 Dec;15(6):542-6. |
| Hanssen, 2017 | Hanssen H, Minghetti A, Magon S, Rossmeissl A, Rasenack M, Papadopoulou A, Klenk C, Faude O, Zahner L, Sprenger T, Donath L. Effects of different endurance exercise modalities on migraine days and cerebrovascular health in episodic migraineurs: A randomized controlled trial. Scand J Med Sci Sports. 2018 Mar;28(3):1103-1112. |
| Hanten, 2002 | William P. Hanten, Sharon L. Olson & Greta Matson Ludwig (2002) Reliability of Manual Mobility Testing of the Upper Cervical Spine in Subjects with Cervicogenic Headache, Journal of Manual & Manipulative Therapy, 10:2, 76-82 |
| Heredia Rizo, 2012 | Heredia Rizo AM, Pascual-Vaca ÁO, Cabello MA, Blanco CR, Pozo FP, Carrasco AL. Immediate effects of the suboccipital muscle inhibition technique in craniocervical posture and greater occipital nerve mechanosensitivity in subjects with a history of orthodontia use: a randomized trial. J Manipulative Physiol Ther. 2012 Jul;35(6):446-53. |
| Hong, 2010 | Hong JP, Lai CH, Lin YC, Chou SW. Clinical assessment of patients with cervicogenic headache: a preliminary study. Chang Gung Med J. 2010 Jan-Feb;33(1):58-66. |
| Jafari, 2017 | Jafari M, Bahrpeyma F, Togha M. Effect of ischemic compression for cervicogenic headache and elastic behavior of active trigger point in the sternocleidomastoid muscle using ultrasound imaging. J Bodyw Mov Ther. 2017 Oct;21(4):933-939. |
| Janani, 2018 | Janani AS, Pope KJ, Fenton N, Grummett TS, Bakhshayesh H, Lewis TW, Watson DH, Whitham EM, Willoughby JO. Resting cranial and upper cervical muscle activity is increased in patients with migraine. Clin Neurophysiol. 2018 Sep;129(9):1913-1919. |
| Jensen, 1988 | Jensen K, Tuxen C, Olesen J. Pericranial muscle tenderness and pressure-pain threshold in the temporal region during common migraine. Pain. 1988 Oct;35(1):65-70. |
| Jensen, 1993 | Jensen R, Rasmussen BK, Pedersen B, Olesen J. Muscle tenderness and pressure pain thresholds in headache. A population study. Pain. 1993 Feb;52(2):193-199. |
| Jull, 1997 | Jull G, Zito G, Trott P, Potter H, Shirley D. Inter-examiner reliability to detect painful upper cervical joint dysfunction. Aust J Physiother. 1997;43(2):125-129. |
| Jull, 2002 | Jull G, Trott P, Potter H, Zito G, Niere K, Shirley D, Emberson J, Marschner I, Richardson C. A randomized controlled trial of exercise and manipulative therapy for cervicogenic headache. Spine (Phila Pa 1976). 2002 Sep 1;27(17):1835-43; discussion 1843. |
| Katsarava, 2003 | Katsarava Z, Giffin N, Diener HC, Kaube H. Abnormal habituation of 'nociceptive' blink reflex in migraine--evidence for increased excitability of trigeminal nociception. Cephalalgia. 2003 Oct;23(8):814-9. |
| Kidd, 1993 | Kidd RF, Nelson R. Musculoskeletal dysfunction of the neck in migraine and tension headache. Headache. 1993 Nov-Dec;33(10):566-9. |
| Kirthika, 2018 | Kirthika, Veena & .s, Sudhakar & Kumar, Vijaya & Kuppuswamy, Padmanabhan. Is mulligan’s sustained natural apophyseal glides (Snags) or muscle energy technique is effective in the non-surgical management of cervicogenic headache? a two-group pretest-posttest randomized controlled trial. 2018. Asian Journal of Pharmaceutical and Clinical Research. 11. 230. |
| Landgraf, 2017 | Landgraf MN, Biebl JT, Langhagen T, Hannibal I, Eggert T, Vill K, Gerstl L, Albers L, von Kries R, Straube A, Heinen F. Children with migraine: Provocation of headache via pressure to myofascial trigger points in the trapezius muscle? - A prospective controlled observational study. Eur J Pain. 2018 Feb;22(2):385-392. |
| Lawler, 2006 | Lawler SP, Cameron LD. A randomized, controlled trial of massage therapy as a treatment for migraine. Ann Behav Med. 2006 Aug;32(1):50-9. |
| Lim, 2018 | Lim, Y. H., Kim, J. S., Lee, H. W., & Kim, S. H. (2018). Postural Instability Induced by Visual Motion Stimuli in Patients With Vestibular Migraine. Frontiers in neurology, 9, 433. https://doi.org/10.3389/fneur.2018.00433 |
| Lin, 2012 | Lin YC, Lai CH, Chang WH, Tu LW, Lin JC, Chou SW. Immediate effects of ischemic compression on neck function in patients with cervicogenic cephalic syndrome. J Manipulative Physiol Ther. 2012 May;35(4):301-7. |
| Lous, 1982 | Lous I, Olesen J. Evaluation of pericranial tenderness and oral function in patients with common migraine, muscle contraction headache and 'combination headache'. Pain. 1982 Apr;12(4):385-393. |
| Makofsky, 2012 | Makofsky HW, Douris P, Goldstein LB, Discepolo A, Grion K, Kushnir G, Malner S, Singh J, Urgolites L, Zic F, Oricchio J. The effect of the PostureJac on deep cervical flexor endurance: implications in the management of cervicogenic headache and mechanical neck pain. Cranio. 2011 Jul;29(3):187-93. |
| Malo-urribés, 2017 | Malo-Urriés M, Tricás-Moreno JM, Estébanez-de-Miguel E, Hidalgo-García C, Carrasco-Uribarren A, Cabanillas-Barea S. Immediate Effects of Upper Cervical Translatoric Mobilization on Cervical Mobility and Pressure Pain Threshold in Patients With Cervicogenic Headache: A Randomized Controlled Trial. J Manipulative Physiol Ther. 2017 Nov-Dec;40(9):649-658. |
| Mingels, 2018 | Mingels S, Granitzer M. Pericranial Tenderness in Females With Episodic Cervical Headache vs Asymptomatic Controls: A Cross-sectional Study. J Manipulative Physiol Ther. 2018 Jul-Aug;41(6):488-495. |
| Mohamed, 2019 | Mohamed AA, Shendy WS, Semary M, Mourad HS, Battecha KH, Soliman ES, Sayed SHE, Mohamed GI. Combined use of cervical headache snag and cervical snag half rotation techniques in the treatment of cervicogenic headache. J Phys Ther Sci. 2019 Apr;31(4):376-381. |
| Mongini, 2005 | Mongini F, Deregibus A, Rota E. Psychiatric disorders and muscle tenderness in episodic and chronic migraine. Expert Rev Neurother. 2005 Sep;5(5):635-42. |
| Mongini, 2005b | Mongini F, Rota E, Deregibus A, Mura F, Francia Germani A, Mongini T. A comparative analysis of personality profile and muscle tenderness between chronic migraine and chronic tension-type headache. Neurol Sci. 2005 Oct;26(4):203-7. |
| Oksanen, 2007 | Oksanen A, Pöyhönen T, Metsähonkala L, Anttila P, Hiekkanen H, Laimi K, Salminen JJ. Neck flexor muscle fatigue in adolescents with headache: an electromyographic study. Eur J Pain. 2007 Oct;11(7):764-72. |
| Oksanen, 2008 | Airi Oksanen, Erkintalo M, Metsähonkala L, Anttila P, Laimi K, Hiekkanen H, Salminen JJ, Aromaa M, Sillanpää M. Neck muscles' cross-sectional area in adolescents with and without headache - MRI study. Eur J Pain. 2008 Oct;12(7):952-9. |
| Oksanen, 2008b | Oksanen A, Pöyhönen T, Ylinen JJ, Metsähonkala L, Anttila P, Laimi K, Hiekkanen H, Aromaa M, Salminen JJ, Sillanpää M. Force production and EMG activity of neck muscles in adolescent headache. Disabil Rehabil. 2008;30(3):231-9. |
| Olivier, 2018 | Olivier B, Pramod A, Maleka D. Trigger Point Sensitivity Is a Differentiating Factor between Cervicogenic and Non-Cervicogenic Headaches: A Cross-Sectional, Descriptive Study. Physiother Can. 2018 Fall;70(4):323-329. |
| Palacios-Ceña, 2017 | Palacios-Ceña M, Ferracini GN, Florencio LL, Ruíz M, Guerrero ÁL, Arendt-Nielsen L, Fernández-de-Las-Peñas C. The Number of Active But Not Latent Trigger Points Associated with Widespread Pressure Pain Hypersensitivity in Women with Episodic Migraines. Pain Med. 2017 Dec 1;18(12):2485-2491. |
| Prakash, 2016 | Prakash S, Rathore C, Makwana P, Dave A. A Cross-Sectional Clinic-Based Study in Patients With Side-Locked Unilateral Headache and Facial Pain. Headache. 2016 Jul;56(7):1183-93. |
| Premlata, 2019 | Rishi, Priyanka. Effect of positional release technique versus ischemic compression on pressure pain threshold, range of motion, and headache disability in cervicogenic headache patients among college going students: A Randomized Controlled Trial.2019. International Journal of Physiotherapy and Research. 6. 140-148. |
| Rinne, 2015 | Rinne M, Garam S, Häkkinen A, Ylinen J, Kukkonen-Harjula K, Nikander R. Therapeutic Exercise Training to Reduce Chronic Headache in Working Women: Design of a Randomized Controlled Trial. Phys Ther. 2016 May;96(5):631-40. |
| Rossi, 2005 | Rossi C, Alberti A, Sarchielli P, Mazzotta G, Capocchi G, Faralli M, Ricci G, Molini E, Altissimi G. Balance disorders in headache patients: evaluation by computerized static stabilometry. Acta Neurol Scand. 2005 Jun;111(6):407-13. |
| Ruscheweyh, 2019 | Ruscheweyh R, Pereira D, Hasenbring MI, Straube A. Pain-related avoidance and endurance behaviour in migraine: an observational study. J Headache Pain. 2019 Jan 18;20(1):9. |
| Russo, 2018 | Russo, A., Coppola, G., Pierelli, F., Parisi, V., Silvestro, M., Tessitore, A., & Tedeschi, G. (2018). Pain Perception and Migraine. Frontiers in neurology, 9, 576. https://doi.org/10.3389/fneur.2018.00576 |
| Saha, 2019 | Saha, F. J., Pulla, A., Ostermann, T., Miller, T., Dobos, G., & Cramer, H. (2019). Effects of occlusal splint therapy in patients with migraine or tension-type headache and comorbid temporomandibular disorder: A randomized controlled trial. Medicine, 98(33), e16805. https://doi.org/10.1097/MD.0000000000016805 |
| Sand, 1997 | Sand T, Zwart JA, Helde G, Bovim G. The reproducibility of cephalic pain pressure thresholds in control subjects and headache patients. Cephalalgia. 1997 Nov;17(7):748-55. |
| Satpute, 2018 | Satpute K, Nalband S, Hall T. The C0-C2 axial rotation test: normal values, intra- and inter-rater reliability and correlation with the flexion rotation test in normal subjects. J Man Manip Ther. 2019 May;27(2):92-98. |
| Schäfer, 2018 | Schäfer A, Lüdtke K, Breuel F, Gerloff N, Knust M, Kollitsch C, Laukart A, Matej L, Müller A, Schöttker-Königer T, Hall T. Validity of eyeball estimation for range of motion during the cervical flexion rotation test compared to an ultrasound-based movement analysis system. Physiother Theory Pract. 2018 Aug;34(8):622-628. |
| Sedighi, 2017 | Sedighi A, Nakhostin Ansari N, Naghdi S. Comparison of acute effects of superficial and deep dry needling into trigger points of suboccipital and upper trapezius muscles in patients with cervicogenic headache. J Bodyw Mov Ther. 2017 Oct;21(4):810-814. |
| Seo, 2018 | Seo JG, Park SP. Clinical significance of sensory hypersensitivities in migraine patients: does allodynia have a priority on it? Neurol Sci. 2019 Feb;40(2):393-398. |
| Shevel, 2004 | Shevel, E., & Spierings, E. H. (2004). Cervical muscles in the pathogenesis of migraine headache. The Journal of Headache and Pain, 5(1), 12–14. |
| Silva Jr, 2014 | Silva AA Jr, Brandão KV, Faleiros BE, Tavares RM, Lara RP, Januzzi E, Carvalho AB, Carvalho EM, Gomes JB, Leite FM, Alves BM, Gómez RS, Teixeira AL. Temporo-mandibular disorders are an important comorbidity of migraine and may be clinically difficult to distinguish them from tension-type headache. Arq Neuropsiquiatr. 2014 Feb;72(2):99-103. |
| Sjaastad, 2008 | Sjaastad O. Cervicogenic headache: comparison with migraine without aura; Vågå study. Cephalalgia. 2008 Jul;28 Suppl 1:18-20. |
| Smith, 2008 | Smith K, Hall T, Robinson K. The influence of age, gender, lifestyle factors and sub-clinical neck pain on the cervical flexion-rotation test and cervical range of motion. Man Ther. 2008 Dec;13(6):552-9. |
| Sorrell, 2006 | Sorrell MR. The physical examination of migraine. Curr Pain Headache Rep. 2006 Oct;10(5):350-4. |
| Sorrell, 2010 | Michael R. Sorrell. Myofascial Examination Leads to Diagnosis and Successful Treatment of Migraine Headache. Journal of Musculoskeletal Pain, 2010, 18:1, 31-37, |
| Szikszay, 2018 | Szikszay TM, Luedtke K, Harry von P. Increased mechanosensivity of the greater occipital nerve in subjects with side-dominant head and neck pain - a diagnostic case-control study. J Man Manip Ther. 2018 Aug;26(4):237-248. |
| Tassorelli, 2018 | Tassorelli C, Grazzi L, de Tommaso M, Pierangeli G, Martelletti P, Rainero I, Dorlas S, Geppetti P, Ambrosini A, Sarchielli P, Liebler E, Barbanti P; PRESTO Study Group. Noninvasive vagus nerve stimulation as acute therapy for migraine: The randomized PRESTO study. Neurology. 2018 Jul 24;91(4):e364-e373. |
| Tolentino, 2018 | Tolentino GA, Bevilaqua-Grossi D, Carvalho GF, Carnevalli APO, Dach F, Florencio LL. Relationship Between Headaches and Neck Pain Characteristics With Neck Muscle Strength. J Manipulative Physiol Ther. 2018 Oct;41(8):650-657. |
| Ünal-Artik, 2017 | Ünal-Artık HA, İnan LE, Ataç-Uçar C, Yoldaş TK. Do bilateral and unilateral greater occipital nerve block effectiveness differ in chronic migraine patients? Neurol Sci. 2017 Jun;38(6):949-954. |
| Uthaikhup, 2009 | Uthaikhup S, Sterling M, Jull G. Cervical musculoskeletal impairment is common in elders with headache. Manual Therapy. 2009 Dec;14(6):636-641. |
| Uthaikhup, 2015 | Uthaikhup S, Prasert R, Paungmali A, Boontha K (2015) Altered Pain Sensitivity in Elderly Women with Chronic Neck Pain. PLoS ONE 10(6): e0128946. |
| Uthaikhup, 2016 | Uthaikhup S, Assapun J, Watcharasaksilp K, Jull G. Effectiveness of physiotherapy for seniors with recurrent headaches associated with neck pain and dysfunction: a randomized controlled trial. Spine J. 2017 Jan;17(1):46-55. |
| Uthaikhup, 2017 | Uthaikhup S, Assapun J, Kothan S, Watcharasaksilp K, Elliott JM. Structural changes of the cervical muscles in elder women with cervicogenic headache. Musculoskelet Sci Pract. 2017 Jun;29:1-6. |
| Van Suijlekom, 2000 | Van Suijlekom HA, De Vet HC, Van Den Berg SG, Weber WE. Interobserver reliability in physical examination of the cervical spine in patients with headache. Headache. 2000 Jul-Aug;40(7):581-6. |
| Varkey, 2017 | Varkey E, Grüner Sveälv B, Edin F, Ravn-Fischer A, Cider Å. Provocation of Migraine after Maximal Exercise: A Test-Retest Study. Eur Neurol. 2017;78(1-2):22-27. |
| Vavrek, 2010 | Vavrek D, Haas M, Peterson D. Physical examination and self-reported pain outcomes from a randomized trial on chronic cervicogenic headache. J Manipulative Physiol Ther. 2010 Jun;33(5):338-48 |
| Vincent, 1999 | Vincent MB, Luna RA. Cervicogenic headache: a comparison with migraine and tension-type headache. Cephalalgia. 1999 Dec;19 Suppl 25:11-6. |
| Voigt, 2011 | Voigt K, Liebnitzky J, Burmeister U, Sihvonen-Riemenschneider H, Beck M, Voigt R, Bergmann A. Efficacy of osteopathic manipulative treatment of female patients with migraine: results of a randomized controlled trial. J Altern Complement Med. 2011 Mar;17(3):225-30. |
| Vonpiekartz, 2007 | von Piekartz HJ, Schouten S, Aufdemkampe G. Neurodynamic responses in children with migraine or cervicogenic headache versus a control group. A comparative study. Man Ther. 2007 May;12(2):153-60. |
| Vonpiekartz, 2011 | von Piekartz H, Lüdtke K. Effect of treatment of temporomandibular disorders (TMD) in patients with cervicogenic headache: a single-blind, randomized controlled study. Cranio. 2011 Jan;29(1):43-56. |
| Vuralli, 2016 | Vuralli D, Evren Boran H, Cengiz B, Coskun O, Bolay H. Chronic Migraine Is Associated With Sustained Elevation of Somatosensory Temporal Discrimination Thresholds. Headache. 2016 Oct;56(9):1439-1447. |
| Watson, 1993 | Watson DH, Trott PH. Cervical headache: an investigation of natural head posture and upper cervical flexor muscle performance. Cephalalgia. 1993 Aug;13(4):272-84; discussion 232. |
| Watson, 2014 | Watson DH, Drummond PD. Cervical referral of head pain in migraineurs: effects on the nociceptive blink reflex. Headache. 2014 Jun;54(6):1035-45. |
| Weber Hellstenius, 2009 | Weber Hellstenius SA. Recurrent neck pain and headaches in preadolescents associated with mechanical dysfunction of the cervical spine: a cross-sectional observational study with 131 students. J Manipulative Physiol Ther. 2009 Oct;32(8):625-34. |
| Ylinen, 2010 | Ylinen J, Nikander R, Nykänen M, Kautiainen H, Häkkinen A. Effect of neck exercises on cervicogenic headache: a randomized controlled trial. J Rehabil Med. 2010 Apr;42(4):344-9. |
| Youssef, 2013 | Youssef EF, Shanb AS. Mobilization versus massage therapy in the treatment of cervicogenic headache: a clinical study. J Back Musculoskelet Rehabil. 2013;26(1):17-24. |
