# Supplementary material for: Differentiating migraine, cervicogenic headache and asymptomatic individuals based on physical examination findings: a systematic review and meta-analysis
Source: BMC Musculoskelet Disord. 2021 Sep 3;22:755. doi: 10.1186/s12891-021-04595-w (PMC8417979; doi:10.1186/s12891-021-04595-w)
Supplement: Supplementary file 2 — Additional file 2. Studies characteristics. [file 12891_2021_4595_MOESM2_ESM.docx]

**Additional file 2.** Studies characteristics.

|  | Individual Characteristics | | | | | Assessment | | | | Results [mean, SD] | |
| --- | --- | --- | --- | --- | --- | --- | --- | --- | --- | --- | --- |
|  |  | No (%) of | | Age (mean, SD) | |  |  |  |  |  |  |
| Study | Diagnosis | Sympto  matic (M; CGH) | Asympto  matic indivi  duals | Sympto  matic (M; CEH) | Asympto  matic indivi  duals | Mean (SD). Headache frequency, d/mo | No (%) participant pain free at examination | Test evaluated | Test procedure or location | Symptomatic indiividuals | Asympto  matic indivi  duals |
| Akdal, 2009^39^ | ICDH-II | 25 M (50%) | 25 (50%) | 36.2 (9.2) | 36.2 (9.3) | 1.54, (1.16) | 25 (100%) | mCTSIB, static posturography | Postural sway, degrees/second | 0.8 (0.1) | 0.6 (0.0.1) |
|  |  |  |  |  |  |  |  | LOS, static posturography | Average reaction time, s | 1.0 (0.1) | 0.7 (0.03) |
|  |  |  |  |  |  |  |  |  | Movement velocity, degrees/seconds | 3.8 (1.2) | 3.6 (1.1) |
|  |  |  |  |  |  |  |  |  | Maximum excursion, % | 101 (3) | 98 (4.5) |
|  |  |  |  |  |  |  |  |  | Directional control, % | 85 (2) | 86 (4.5) |
|  |  |  |  |  |  |  |  | Tandem walking, static posturography | Step width (cm) | 9.6 (0.9) | 8.5 (1.2) |
|  |  |  |  |  |  |  |  |  | Walk speed (cm/s) | 17.3 (3.1) | 22.3 (6.7) |
| Assapun, 2017^81^ | CHISG | 18 CGH (51,42%) | 17 (48,88%) | 64 (3.2) | 65.2 (3.9) | 13.2 (8) | 8 (44.44%) | PPT, kg/cm2, algometer | Anterior part temporalis muscle | 1.6 (0.4) | 1.8 (0.6) |
|  |  |  |  |  |  |  |  |  | Articular pillars C2-C3 | 1.9 (0.6) | 2.1 (0.7) |
| Baron, 2017^62^ | ICDH-III | 162 M (79.41%) | 42 (20.59%) | 38.4 (17.7) | 39 (12.8) | 14.2 (3.8) | 162 (100%) | PPT, kg/cm2, algometer (just recorded here where statistical difference) | Fp1 (frontal point) | 1.95 (1.1) | 2.65 (0.8) |
|  |  |  |  |  |  |  |  |  | Fp2 (frontal point) | 197 (1.1) | 2.75 (0.8) |
| Benatto, 2019^63^ | ICDH-III | 52 M (50%) | 52 (50%) | 33.8 (10.6) | 28.9 (8.3) | 11.9 (8.8) | NR | MVIC Ratio, EMG | Flexion | 0.3 (0.2) | 0.4 (0.2) |
|  |  |  |  |  |  |  |  |  | Extension | 2.8 (1.0) | 3.0 (2.5) |
|  |  |  |  |  |  |  |  | Strength, N, dynamometer | Flexors | 35.4 (14.0) | 48.4 (21.2) |
|  |  |  |  |  |  |  |  |  | Extensors | 86.0 (31.7) | 92.0 (32.2) |
|  |  |  |  |  |  |  |  |  | Ext/F | 0.4 (0.2) | 0.3 (0.2) |
|  |  |  |  |  |  |  |  | CCFT, logarithmic mean, EMG | 22 mmHg | 0.35 (0.2) | 0.4 (0.3) |
|  |  |  |  |  |  |  |  |  | 30 mmHg | 0.4 (0.3) | 0.5 (0.3) |
| Bevilaqua-Grossi, 2009^54^ | ICHD-II | 30 M (66.66%) | 15 (33.33%) | 36.5 (10.5) | 39.9 (10.5) | 13.9 (5.2) | 18 (60%) | ROM, º, goniometer | Flexion | 51.6 (9.6) | 57.6 (10.3) |
|  |  |  |  |  |  |  |  |  | Extension | 63.9 (10.6) | 68.1 (11.3) |
|  |  |  |  |  |  |  |  |  | Rotation, both sides | 60.7 (9.6) | 64.1 (7.3) |
|  |  |  |  |  |  |  |  |  | Lateral flexion, both sides | 44.5 (8.2) | 47.4 (6.4) |
| Bevilaqua-Grossi, 2011^55^ | ICHD-II | 29 M (66%) | 15 (34 %) | 37.1 (10.3) | 39.9 (10.5) | 13.8 (4.9) | NR | PPT, kg/cm2, algometer | 10 points | 1.44 (0.57) to 3.24 (1.03) | 1.78 (0.57) to 3.49 (0.83) |
|  |  |  |  |  |  |  |  |  | Frontal | 2.1 (0.6) | 2.8 (0.7) |
|  |  |  |  |  |  |  |  |  | Anterior part temporalis muscle | 2.5 (0.7) | 2.9 (0.7) |
|  |  |  |  |  |  |  |  |  | Medium part temporalis muscle | 2,5 (0.7) | 2.9 (0.6) |
|  |  |  |  |  |  |  |  |  | Posterior part temporalis muscle | 2.3 (0.9) | 3.4 (0.8) |
|  |  |  |  |  |  |  |  |  | Masseter origin | 1.6 (0.5) | 1.9 (0.5) |
|  |  |  |  |  |  |  |  |  | Masseter belly | 1.7 (0.5) | 1.9 (0.5) |
|  |  |  |  |  |  |  |  |  | Masseter insertion | 1.8 (0.5) | 2.1 (0.5) |
|  |  |  |  |  |  |  |  |  | Trapezius insertion | 2.4 (0.8) | 2.9 (0.8) |
|  |  |  |  |  |  |  |  |  | Trapezius upper | 2.7 (0.9) | 3.4 (0.9) |
|  |  |  |  |  |  |  |  |  | SCM insertion | 2.1 (0.9) | 2.5 (0.6) |
| Bovim, 1992^33^ | IHS | 32 CGH (33.68%); 26 M (27.36%) | 20 (21.05%) | 43 (11) CEH; 34.8 (8.9) M | 42 (15) | NR | 32 CGH (100%); 26 M (100%) | PPT, kg/cm2, 22 points, algometer | Whole head | M: 4.7 (1.6); CEH: 3.47 (0.6) | 5.0 (1.7) |
|  |  |  |  |  |  |  |  |  | Occipital/frontal ratio | M: 0.9 (0.1); CEH: 0.9 (0.1) | 1.0 (0.1) |
|  |  |  |  |  |  |  |  |  | Dominant/non dom. ratio | M: 1.0 (0.1); CEH: 0.9 (0.1) | NR |
| Calandre, 2006^40^ | ICHD-II | 98 M (75.38%) | 32 (24.62%) | 41.4 (16.8) | 38.5 (13.5) | NR | 98 (100%) | Trigger points, %, manual palpation | Supraciliary arch, temporal muscle, suboccipital area, occipital area and trapezius muscle | 93.9 | 29 |
| Carnevalli, 2018^64^ | ICHD-III | 25 M (50%) | 25 (50%) | 27 (10) | 27 (9) | 9.1 (6.3) | 25 (100%) | Neck muscle strength, N, dynamometer | Flexors | 35.0 (5.3) | 37.7 (5.8) |
|  |  |  |  |  |  |  |  |  | Extensors | 85.4 (14.71) | 100.3 (8.4) |
|  |  |  |  |  |  |  |  |  | Right lateral flexion | 62.8 (8.04) | 66.6 (8.4) |
|  |  |  |  |  |  |  |  |  | Left lateral flexion | 61.5 (10.2) | 67.1 (8.5) |
| Carvalho, 2013^41^ | ICHD-II | 62 M (67.39) | 30 (32.21) | 37.5 (9) | 33 (9) | 7 (3) | NR | Postural oscillation, cm2, force plates | OE | 2.3 (1.3) | 1.5 (0.8) |
|  |  |  |  |  |  |  |  |  | CE | 2.9 (2.5) | 1.7 (1.2) |
|  |  |  |  |  |  |  |  |  | ROE | 5.7 (2.1) | 5.0 (1.8) |
|  |  |  |  |  |  |  |  |  | LOE | 5.6 (2.0) | 5.1 (1.4) |
|  |  |  |  |  |  |  |  | Mobility | TUG test, seconds | 8.35 (1.2) | 6.5 (0.9) |
| Carvalho, 2016^65^ | ICHD-III | 105 M (75%) | 35 (25%) | 36 (14.1) | 36 (7.7) | 10 (1.8) | 105 (100%) | MSOT, cm2, BioDynamicsBr Analysis software | Firm surface | 3.1 (3.02) | 1.4 (1) |
|  |  |  |  |  |  |  |  |  | Foam surface | 14.1 (8.87) | 9.95 (2.33) |
|  |  |  |  |  |  |  |  | LOS, Balance Master System | Reaction time, seconds | 0.73 (0.71) | 0.97 (0.4) |
| Chen, 2018^82^ | CHISG | 13 CGH (48%) | 14 (52%) | 24.5 (4.8) | 23.9 (2.7) | NR | 13 (100%) | Thickness measurement, mm, ultrasound | Oblique capitis inferior, rest | 10.7 (2.9) | 10.3 (1.8) |
|  |  |  |  |  |  |  |  |  | Oblique capitis inferior, contraction | 12.6 (3.0) | 12.3 (2.0) |
|  |  |  |  |  |  |  |  | Cervical kinesthesia, º, ultrasound-based motion analysis | HRT, H-ECNHP, CE | 2.6 (4.4) | 4.3 (3.6) |
|  |  |  |  |  |  |  |  |  | HRT, H-TT, CE | 1.7 (4.5) | 2.9 (4.1) |
|  |  |  |  |  |  |  |  |  | HRT, H-ECNHP, AE | 4.4 (2.7) | 4.8 (2.4) |
|  |  |  |  |  |  |  |  |  | HRT, H-TT, AE | 4.2 (2.3) | 4.1 (2.2) |
| Cooke, 2007^79^ | Other | 15 M (50%) | 15 (50%) | 36.1 (9.3) | 36.7 (8.7) | >15 | NR | Von Frey Hairs, numbers | Face | 53.2 (5.6) | 142.6 (3.8) |
|  |  |  |  |  |  |  |  |  | Trapezius | 40.8 (7.8) | 144 (3.6) |
|  |  |  |  |  |  |  |  |  | Forearm | 53. 3 (7.2) | 134. 8 (3.4) |
| De Hertogh, 2008^83^ | CHISG | 10 CGH (29,4%) | 23 (70,6%) | 41 (16) | 34 (12) | 7.6 (NR) | 10 (100%) | Cervical kinesthesia, cm, electromagnetic tracking device | Flexion | 1.2 (0.7) | 1.2 (0.8) |
|  |  |  |  |  |  |  |  |  | Extension | 1.4 (0.7) | 1.1 (0.5) |
|  |  |  |  |  |  |  |  |  | Rotation, both sides | 1.2 (0.6) | 1.1 (0.6) |
| Drummond, 1987^80^ | Other | 68 M (49.63%) | 35 (25.54%) | 42 (-) | 39 (-) | NR | 68 (100%) | Scalp tenderness (range 0-7), algometer | Forehead | 3.2 (NR) | 1.1 (NR) |
|  |  |  |  |  |  |  |  |  | Temples | 4.2 (NR) | 1.4 (NR) |
|  |  |  |  |  |  |  |  |  | Occiput | 4.2 (NR) | 1.2 (NR) |
|  |  |  |  |  |  |  |  |  | Neck | 4.3 (NR) | 1.6 (?) |
| Dugailly, 2017^66^ | ICHD-III | 30 M (23.44%) | 80 (74.1%) | 36 (11) | 37 (12) | NR | 30 (100%) | Stiffness (nm/*), torsionmeter | Right | 0.06 (0.05) | 0.07 (0.06) |
|  |  |  |  |  |  |  |  |  | Left | 0.05 (0.04) | 0.06 (0.04) |
| Dumas, 2001^34^ | CHISG (CEH) / IHS (M) | 24 CGH (31.16%) 16 M (20.77%) | 17(22.07%) | CGH: 44 (11.9) / M: 39 (12.5) | 43 (14.1) | CGH: 16.8 (9.1) / M: 5.2 (3.1) | NR | Neck mobility, ROM, º, goniometer | Flex + Ext | CEH: 114 (13.8) / M: 125 (16.5) | 123 (15.5) |
|  |  |  |  |  |  |  |  |  | Rot (both) | CEH: 132 (11.1) / M: 142 (12.3) | 136 (14.0) |
|  |  |  |  |  |  |  |  |  | LF (both) | CEH: 75 (17.4) / M: 82 (18.8) | 76 (16.1) |
|  |  |  |  |  |  |  |  | Cervical kinesthesia, goniometer, º | Rot both sides 30º | CEH: 5.5 (4.0) / M: 6.0 (3.9) | 4.7 (3.1) |
|  |  |  |  |  |  |  |  |  | Rot both sides 50º | CEH: 3.2 (2.8) / M 3.9 (3.2) | 3.6 (2.2) |
|  |  |  |  |  |  |  |  |  | LF both sides 20º | CEH 3.9 (3.8) / M: 3.7 (3.5) | 3.1 (1.9) |
|  |  |  |  |  |  |  |  | Posture CVA, º, photograph | Sitting position | CEH: 50.4 (5.2) / M: 49.0 (8.3) | 47.4 (6.3) |
|  |  |  |  |  |  |  |  | Strength, N, dynamometer | Extensors | CGH: 126.6 (43.4) / M: 150.6 (58.9) | 170.8 (67.9) |
|  |  |  |  |  |  |  |  |  | Flexors | CGH: 59.9 (31.7) / M: 76.54 (51.2) | 97.32 (40.1) |
|  |  |  |  |  |  |  |  | Endurance, s, dynamometer | Short neck flexors | CGH: 13.9 (5.3) / M: 14.2 (6.5) | 18.9 (7.7) |
|  |  |  |  |  |  |  |  | PAIVM, %, manual palpation | Cervical spine | CGH: 16.8 to 58.3 / M: 6.1 to 52.4 | 4.4 to 67.3 |
|  |  |  |  |  |  |  |  | PPIVM, %, manual palpation | Cervical spine | CGH: 13.8 to 48.6 / M: 17.8 to 43.5 | 12.4 to 50.1 |
|  |  |  |  |  |  |  |  | Skin roll test, VAS 0-100, manual palpation | Trapezius | CGH: 54.85 (31.8)/ M: 33.5 (32.4) | 16.5 (14.9) |
|  |  |  |  |  |  |  |  |  | Mandible | CGH: 14.6 (15.7) / M: 10.5 (13.8) | 5.3 (4.7) |
| Engstrom, 2013^42^ | ICHD-II | 33 M (49%) | 34 (51%) | 36.4 (12.9) | 39.6 (13.7) | 2.3 (1.9) | 33 (100% | PPT, kg/cm2, algometer | Average: splenius, trapezius, temporalis, second finger | 5.6 (1.4) | 6.7 (2.5) |
| Engstrom, 2013b^43^ | ICHD-II | 50 M (59.5%) | 34 (30.5%) | 38.1 (12.5) | 39.6 (13.7) | 2.2 (0.7) | 50 (100%) | PPT, kg/cm2, algometer | Average: splenius, trapezius, temporalis, second finger | 6.4 (2.3) | 6.7 (2.5) |
| Engstrom, 2014^44^ | ICHD-II | 53 M (63.1%) | 34 (36.9%) | 38.2 (12) | 39.6 (13.7) | 2.2 (0.7) | 53 (100%) | PPT, kg/cm2, algometer | Average: splenius, trapezius, temporalis, second finger | 5.6 (1.4) | 6.7 (2.5) |
| Fernandez-de-las-Peñas, 2006^45^ | ICHD-II | 20 M (50%) | 20 (50%) | 33 (10) | 30 (8) | 3-6 | 20 (100%) | Latent trigger points, %, manual palpation | Suboccipital muscles | 40 | 3.3 |
|  |  |  |  |  |  |  |  |  | SCM | 27.5 | 20 |
|  |  |  |  |  |  |  |  |  | Temporalis muscle | 17.5 | 8.4 |
|  |  |  |  |  |  |  |  |  | Upper trapezius muscle | 32.5 | 25 |
|  |  |  |  |  |  |  |  | Posture, CVA, º photograph | Sitting position | 42.2 (6.4) | 52.6 (7.2) |
|  |  |  |  |  |  |  |  |  | Standing position | 44.7 (9.6) | 53.7 (7.2) |
|  |  |  |  |  |  |  |  | ROM, º, goniometer | Flexion | 57.0 (11.5) | 61.0 (6.8) |
|  |  |  |  |  |  |  |  |  | Extension | 60.6 (9.6) | 68.9 (12.7) |
|  |  |  |  |  |  |  |  |  | Rotation, both sides | 67.7 (10.9) | 72.7 (5.9) |
|  |  |  |  |  |  |  |  |  | Lateral flexion, both sides | 35.5 (6.0) | 39.3 (6.5) |
| Fernandez-de-las-Peñas, 2006b^46^ | ICHD-II | 20 M (50%) | 20 (50%) | 33 (10) | 34 (10) | 3-4 | 20 (100%) | Trigger point in trochlear region, VAS, manual palpation | Local pain | 4.9 (1.6) | 2.1 (1.1) |
|  |  |  |  |  |  |  |  |  | Referred pain with compression | 5.2 (1.3) | 2.7 (0.4) |
|  |  |  |  |  |  |  |  |  | Referred pain with contraction | 6.1 (1.7) | 3 (0.5) |
|  |  |  |  |  |  |  |  |  | Referred pain with stretching | 6.2 (1.8) | 2.6 (0.7) |
| Fernandez-de-las-Peñas, 2008^47^ | ICHD-II | 25 M (50%) | 25 (50%) | 32 (7) | 31 (9) | 3-4 | 25 (100%) | Total tenderness score, (0-10) manual palpation | 8 muscles from craniocervical region | 8.1 (3.5) | 6.0 (2.5) |
|  |  |  |  |  |  |  |  | PPT, kg/cm2, algometer | Upper trapezius muscle | 1.9 (0.6) | 2.5 (0.5) |
|  |  |  |  |  |  |  |  |  | Anterior part temporalis muscle | 1.8 (0.6) | 2.3 (0.4) |
| Fernandez-de-las-Peñas, 2009^48^ | ICHD-II | 20 M (50%) | 20 (50%) | 36 (11) | 35 (8) | 2.5 (NR) | 20 (100%) | PPT, kg/cm2, algometer | Median nerve | 2.0 (0.5) | 2.8 (0.5) |
|  |  |  |  |  |  |  |  |  | Ulnar nerve | 2.9 (0.4) | 3.8 (0.7) |
|  |  |  |  |  |  |  |  |  | Radial nerve | 2.5 (0.7) | 3.3 (0.5) |
|  |  |  |  |  |  |  |  |  | Supraorbital nerve | 1.2 (0.3) | 1.9 (0.3) |
| Fernandez-de-las-Peñas, 2009b^49^ | ICHD-II | 15 M (50%) | 15 (50%) | 36 (10) | 37 (6) | 2.7 (0.7) | 15 (100%) | PPT, kg/cm2, algometer | 9 points of temporalis muscle | 2.13 (0.68) to 2.73 (0.72) | 3.10 (0.46) to 3.47 (0.39) |
|  |  |  |  |  |  |  |  |  | Anterior part temporalis muscle | 2.3 (0.7) | 3.2 (0.4) |
|  |  |  |  |  |  |  |  |  | Central part temporalis muscle | 2.4 (0.6) | 3.2 (0.4) |
|  |  |  |  |  |  |  |  |  | Posterior part temporalis muscle | 2.7 (0.7) | 3.4 (0.5) |
| Fernandez-de-las-Peñas, 2010^50^ | ICHD-II | 20 M (50%) | 20 (50%) | 37 (9) | 37 (8) | 3 (NR) | 20 (100%) | PPT, kg/cm2, algometer | 11 points of trapezius muscle | 2.1 (0.4) to 3.41 (0.3) | 3.4 (0.2) to 3.9 (0.4) |
|  |  |  |  |  |  |  |  |  | Point 1 | 2.5 (0.5) | 3.4 (0.4) |
|  |  |  |  |  |  |  |  |  | Point 2 | 2.2 (0.5) | 3.4 (0.4) |
|  |  |  |  |  |  |  |  |  | Point 3 | 2.8 (0.6) | 3.8 (0.3) |
|  |  |  |  |  |  |  |  |  | Point 4 | 2.9 (0.7) | 3.5 (0.3) |
|  |  |  |  |  |  |  |  |  | Point 5 | 3.0 (0.5) | 3.5 (0.2) |
|  |  |  |  |  |  |  |  |  | Point 6 | 3.2 (0.5) | 3.6 (0.2) |
|  |  |  |  |  |  |  |  |  | Point 7 | 3.1 (0.6) | 3.7 (0.3) |
|  |  |  |  |  |  |  |  |  | Point 8 | 2.9 (0.6) | 3.5 (0.3) |
|  |  |  |  |  |  |  |  |  | Point 9 | 3.3 (0.5) | 3.7 (0.2) |
|  |  |  |  |  |  |  |  |  | Point 10 | 3.3 (0.4) | 3.4 (0.2) |
|  |  |  |  |  |  |  |  |  | Point 11 | 3.3 (0.3) | 3.6 (0.4) |
| Ferracini, 2016^51^ | ICHD-II | 33 M (50%) | 33 (50%) | 32 (11.3) | 33 (12.6) | 12.9 (8.2) | 19 (58%) | Posture, CVA, º, photograph | Standing position | 46.1 (5.3) | 44.5 (5.1) |
| Ferracini, 2017^67^ | ICHD-III | 71 M (76.34%) | 22 (23.66%) | 41.7 (12.2) | 44 (11) | 14 (9.4) | NR | ROM,º, goniometer | Flexion | 50.1 (14.7) | 51.4 (11.7) |
|  |  |  |  |  |  |  |  |  | Extension | 59.3 (12.6) | 65.9 (14.0) |
|  |  |  |  |  |  |  |  |  | Rotation, both sides | 62.4 (8.9) | 69.5 (9.9) |
|  |  |  |  |  |  |  |  |  | Lateral flexion, both sides | 38.7 (9.4) | 43.2 (10.9) |
|  |  |  |  |  |  |  |  |  | FRT, % | 27 | 42.2 |
|  |  |  |  |  |  |  |  | PAIVM, %, manual palpation | C0-C1 | 23.6 | (NR) |
|  |  |  |  |  |  |  |  |  | C1-C2 | 22.7 | (NR) |
|  |  |  |  |  |  |  |  | Cervical kinesthesia, º, laser | Extension | 4.6 (2.3) | 4.8 (2.3) |
|  |  |  |  |  |  |  |  |  | Rotation (both sides) | 6.0 (2.2) | 5.8 (2.1) |
|  |  |  |  |  |  |  |  | Posture CVA, º, photograph | Standing position | 35.4 (6.9) | 37.9 (5.3) |
|  |  |  |  |  |  |  |  |  | Sitting position | 40.0 (5.2) | 41.4 (4.4) |
|  |  |  |  |  |  |  |  | Posture cervical lordosis, º, photograph | Standing position | 5.7 (2.0) | 6.7 (2.9) |
|  |  |  |  |  |  |  |  |  | Sitting position | 7.9 (2.0) | 8.6 (3.1) |
| Ferreira, 2014^52^ | ICHD-II | 22 M (33.33%) | 22 (33.33%) | 31.7 (9.8) | 24.4 (7.0) | 8.3 (NR) | 22 (100%) | Posture CVA, º, photograph | Standing position | 69.9 (3.1) | 73.0 (5.3) |
|  |  |  |  |  |  |  |  | Posture cervical lordosis, º, photograph | Standing position | 15.0 (1.0) | 16.4 (1.6) |
|  |  |  |  |  |  |  |  | Posture thoracic kyphosis, º, photograph | Standing position | 42.9 (4.0) | 47.3 (4.2) |
| Florencio, 2015^53^ | ICHD-II | 52 M (62.65%) | 31 (37.35%) | 33.4 (10.6) | 31 (9.1) | 11.8 (9.2) | 52 (100%) | Strength, N, dynamomter | Flexion | 39.7 (11.0) | 41.5 (9.9) |
|  |  |  |  |  |  |  |  |  | Extension | 95.9 (25.2) | 113.1 (17.9) |
|  |  |  |  |  |  |  |  |  | Lateral flexion | 60.9 (15.1) | 70.2 (13.4) |
| Florencio, 2015b^68^ | ICHD-III | 30 M (50%) | 30 (50%) | 37 (12) | 32 (10) | 10 (9) | 30 (100%) | PPT, kg/cm2, algometer | Upper trapezius muscle | 2.5 (0.1) | 2.8 (0.2) |
|  |  |  |  |  |  |  |  |  | Suboccipital | 1.6 (0.1) | 2.4 (0.1) |
|  |  |  |  |  |  |  |  |  | SCM | 1.6 (0.1) | 2.5 (0.1) |
|  |  |  |  |  |  |  |  |  | Scalene | 1.4 (0.1) | 2.2 (0.1) |
|  |  |  |  |  |  |  |  |  | Levator scapulae | 1.7 (0.1) | 3.0 (0.1) |
| Florencio, 2016^69^ | ICHD-III | 52 M (62.65%) | 31 (37.35%) | 33.4 (10.6) | 31 (9.1) | 14.6 (10.52) | 52 (100%) | EMG activity during CCFT, 22mmHg, RMS | Upper trapezius muscle | 20.5 (10.8) | 14.42 (6.6) |
|  |  |  |  |  |  |  |  |  | Splenius | 7.6 (5.7) | 6.2 (4.2) |
|  |  |  |  |  |  |  |  |  | SCM | 7.7 (5.5) | 6.0 (3.8) |
|  |  |  |  |  |  |  |  |  | Anterior scalene | 7.7 (6.9) | 5.0 (3.0) |
|  |  |  |  |  |  |  |  | EMG activity during CCFT 30 mmHg, RMS | Upper trapezius muscle | 22.5 (12.0) | 15.3 (7.1) |
|  |  |  |  |  |  |  |  |  | Splenius | 9.4 (6.8) | 7.0 (4.4) |
|  |  |  |  |  |  |  |  |  | SCM | 11.1 (8.7) | 5.9 (7.7) |
|  |  |  |  |  |  |  |  |  | Anterior scalene | 11.1 (8.5) | 7.9 (7.3) |
| Florencio, 2018^70^ | ICHD-III | 68 M (74.72%) | 23 (25.28%) | 44 (14.23) | 43 (11.6) | 11.7 (12.3) | 68 (100%) | Posture CVA, º, photograph | Standing position | 40.0 (5.2) | 41.4 (4.4) |
|  |  |  |  |  |  |  |  |  | Sitting position | 35.3 (7.0) | 37.9 (5.3) |
|  |  |  |  |  |  |  |  | Cervical lordosis angle, photograph, º | Standing position | 7.9 (2.3) | 8.6 (3.1) |
|  |  |  |  |  |  |  |  |  | Sitting position | 5.7 (1.9) | 6.6 (2.9) |
|  |  |  |  |  |  |  |  | EMG activity during CCFT, 22mmHg, RMS | Upper trapezius muscle | 41.9 (26.5) | 35.1 (20.7) |
|  |  |  |  |  |  |  |  |  | Splenius | 13.4 (10.7) | 7.7 (4.8) |
|  |  |  |  |  |  |  |  |  | SCM | 13.6 (10.3) | 16.8 (19.3) |
|  |  |  |  |  |  |  |  |  | Anterior scalene | 24.4 (17.9) | 19.5 (14.8) |
|  |  |  |  |  |  |  |  | EMG activity during CCFT, 30mmHg, RMS | Upper trapezius muscle | 43.9 (24.1) | 31.7 (16.4) |
|  |  |  |  |  |  |  |  |  | Splenius | 18.9 (14.8) | 8.7 (5.3) |
|  |  |  |  |  |  |  |  |  | SCM | 15.5 (11.6) | 20.8 (24.4) |
|  |  |  |  |  |  |  |  |  | Anterior scalene | 29.5 (20.5) | 20.2 (1.8) |
| Florencio, 2019^71^ | ICHD-III | 26 M (50%) | 26 (50% | 29.8 (7.5) | 28.6 (3.7) | 9.3 (7.9) | NR | Muscle endurance, s, chronometer | Flexors | 35.0 (30.0) | 60.5 (36.3) |
|  |  |  |  |  |  |  |  |  | Extensors | 166.5 (135.3) | 290.5 (213.3) |
| Hall, 2004^84^ | CHISG | 28 CGH (50%) | 28 (50%) | 43.3 (11.5) | 43 (13.5) | 13.8 (9) | NR | ROM, º, goniometer | Flexion | 49.1 (9.8) | 51 (9.4) |
|  |  |  |  |  |  |  |  |  | Extension | 58 (15.6) | 60 (9.3) |
|  |  |  |  |  |  |  |  |  | Rotation | 65.2 (9.9) | 65.5 (9.1) |
|  |  |  |  |  |  |  |  |  | Lateral flexion | 35.1 (10.3) | 35.5 (7.9) |
|  |  |  |  |  |  |  |  |  | FRT towards side | 27.6 (6.6) | 44.7 (7.2) |
|  |  |  |  |  |  |  |  |  | FRT from headache side | 42.6 (6.7) | 43.4 (7.8) |
| Hall, 2008^85^ | CHISG | 12 CGH (50%) | 12 (50%) | 29 (4) | 30 (7) | NR | NR | FRT. º, goniometer | Supine, passive | 26 (8.4) | 44.5 (2.8) |
| Hall, 2010^16^ | M: ICHD-II; CGH: CHISG | 20 M (33%); 20 CGH (33%) | NR | M: 30 (6.5); CGH: 35 (10.9) | NR | NR | 40 (100%) | FRT.º, goniometer | Supine, passive | M 41.8 (5.1); CEH: 25.2 (11.1) | NR |
| Hall, 2010b^86^ | CHISG | 72 CGH (78.26%) | 20 (21.7%) | 39 (12.8) | 35 (9.2) | 15.33 (6.63) | 72 (100%) | FRT,º, goniometer | More restricted side | 22.2 (7.7) | 41.5 (6.2) |
| Horwitz, 2015^72^ | ICHD-III | 40 M (46.5%) | 46 (53.5%) | NR | NR | NR | NR | ROM, º, goniometer | Flexion | 53.7 (11.1) | 57.8 (10.9) |
|  |  |  |  |  |  |  |  |  | Extension | 71.2 (11.8) | 73.9 (13.5) |
|  |  |  |  |  |  |  |  |  | Rotation | 85.2 (11.9) | 90.8 (10.1) |
|  |  |  |  |  |  |  |  |  | Lateral flexion | 45.7 (12.3) | 46.5 (9.2) |
|  |  |  |  |  |  |  |  | Strength, N, dynamometer | Flexors | 55.0 (17.4) | 59.3 (17.6) |
|  |  |  |  |  |  |  |  |  | Extensors | 104.9 (22.5) | 107.5 (23.6) |
|  |  |  |  |  |  |  |  |  | Rotators | 67.0 (17.4) | 70.4 (17.2) |
|  |  |  |  |  |  |  |  |  | Lateral flexors | 67.0 (18.9) | 70.5 (19.1) |
|  |  |  |  |  |  |  |  | Muscle length, reduced range, manual examination | Trapezius | > length restriction | NR |
|  |  |  |  |  |  |  |  |  | SCM | > length restriction | NR |
|  |  |  |  |  |  |  |  |  | Deep occipital muscles | > length restriction | NR |
|  |  |  |  |  |  |  |  | Trigger point, %, manual palpation | Trapezius | 79 | 44.6 |
|  |  |  |  |  |  |  |  |  | Scalene | 31.3 | 19.6 |
|  |  |  |  |  |  |  |  |  | SCM | 56.2 | 34 |
|  |  |  |  |  |  |  |  |  | Suboccipital | 47.5 | 32.6 |
|  |  |  |  |  |  |  |  |  | Levator scapulae | 55 | 35.9 |
|  |  |  |  |  |  |  |  | Neural mobility, %, manual examination | ULTT | 25 | 7 |
|  |  |  |  |  |  |  |  | PAIVMS, VAS, manual palpation | C2 | 1.4 (1.68) | 1.24 (1.58) |
|  |  |  |  |  |  |  |  |  | C3 | 2.44 (2.12) | 1.66 (2.0) |
|  |  |  |  |  |  |  |  |  | C4 | 3.21 (2.13) | 2.07 (1.95) |
|  |  |  |  |  |  |  |  |  | C5 | 2.54 (2.29) | 1.54 (2.03) |
|  |  |  |  |  |  |  |  |  | C6 | 2.91 (2.37) | 1.37 (1.9) |
|  |  |  |  |  |  |  |  |  | C7 | 2.15 (2.02) | 1.39 (1.72) |
| Huber, 2012^87^ | CHISG | 40 CGH (66.66%) | 20 (33.33%) | 38.6 (9.4) | 36.4 (8.5) | NR | NR | ROM, º, goniometer | Extension | 26 (4.3) | 35.1 (4.3) |
|  |  |  |  |  |  |  |  |  | Flexion | 28.7 (6.0) | 42.3 (3.7) |
|  |  |  |  |  |  |  |  |  | Rotation | 37.5 (4.0) | 45.8 (6.6) |
|  |  |  |  |  |  |  |  |  | Lateral flexion | 19.1 (3.1) | 27.5 (4.1) |
|  |  |  |  |  |  |  |  | Trigger point, %, manual palpation | SCM | 17.5 | 0 |
|  |  |  |  |  |  |  |  |  | Trapezius | 80 | 0 |
|  |  |  |  |  |  |  |  |  | Erector spinae | 42.5 | 0 |
|  |  |  |  |  |  |  |  | Muscle strength, Lovett's scale, 0-5 | SCM | 3.3 (0.8) | 5 |
|  |  |  |  |  |  |  |  |  | Trapezius | 3.5 (0.9) | 5 |
|  |  |  |  |  |  |  |  |  | Erector spinae | 3.7 (0.7) | 5 |
|  |  |  |  |  |  |  |  | EMG, at rest, amplitude | SCM | 26.0 (6.9) | 19.5 (4.5) |
|  |  |  |  |  |  |  |  |  | Trapezius | 30.1 (8.8) | 24.0 (4.2) |
|  |  |  |  |  |  |  |  |  | Erector spinae | 28.2 (8.6) | 23.9 (4.0) |
|  |  |  |  |  |  |  |  | EMG, maximal contraction, amplitude | SCM | 434.3 (135.9) | 537.5 (79.7) |
|  |  |  |  |  |  |  |  |  | Trapezius | 452.5 (140.4) | 555 (108.8) |
|  |  |  |  |  |  |  |  |  | Erector spinae | 442.5 (91.0) | 490 (74.6) |
| Jull, 2007^19^ | M: ICHD-II; CGH: CHISG | 22 M (16.9%); 18 CGH (13.8%) | 57 (43.8%) | M: 39.8 (12.2); CGH: 40.6 (10.9) | 37.8 (11.3) | NR | NR | CROM, º, goniometer | Flexion | M: 45.7 (10.2); CGH: 47.6 (10.4) | 45.1 (9.0) |
|  |  |  |  |  |  |  |  |  | Extension | M: 54.8 (11.5); CGH: 38.2 (12.6) | 53.8 (12.2) |
|  |  |  |  |  |  |  |  |  | Rotation (both sides) | M: 64.3 (9.3); CGH: 48.5 (8.6) | 65.9 (6.8) |
|  |  |  |  |  |  |  |  |  | Lateral flexion (both sides) | M: 38 (8.2); CGH: 36.7 (8.6) | 40 (6.4) |
|  |  |  |  |  |  |  |  | PAIVMS, %, manual palpation | C0-C1 | M: 2.2; CGH: 47.2 | 0.9 |
|  |  |  |  |  |  |  |  |  | C1-C2 | M: 0; CGH: 38.9 | 0 |
|  |  |  |  |  |  |  |  |  | C2-C3 | M: 0; CGH: 36.0 | 0 |
|  |  |  |  |  |  |  |  |  | C3-C4 | M: 0; CGH: 11.2 | 0 |
|  |  |  |  |  |  |  |  |  | C4-C5 | M: 4.6; CGH: 8.4 | 0.9 |
|  |  |  |  |  |  |  |  |  | C5-C6 | M: 2.3; CGH: 2.3 | 0 |
|  |  |  |  |  |  |  |  |  | C6-C7 | M: 2.3; CGH: 0 | 0 |
|  |  |  |  |  |  |  |  |  | C7-T1 | M: 0; CGH: 2.3 | 0 |
|  |  |  |  |  |  |  |  | Strength, N, dynamometer | Flexion | M: 84.5 (30.9); CGH: 57.8 (26.5) | 89.5 (44.2) |
|  |  |  |  |  |  |  |  |  | Extension | M: 165.7 (52.6); CGH: 109.4 (42.4) | 171.5 (72.0) |
|  |  |  |  |  |  |  |  | EMG during CCFT, normalized RMS | SCM, 22 mmHg | M: 0.27 (0.17); CGH: 0.25 (0.1) | 0.25 (0.13) |
|  |  |  |  |  |  |  |  |  | SCM, 30 mmHg | M: 0.6 (0.31); CGH: 0.85 (0.37) | 0.56 (0.28) |
|  |  |  |  |  |  |  |  | Cervical kinesthesia, Fastrak system | Extension | M: 4.0 (2.4); CGH: 3.9 (2.5) | 3.3 (2.1) |
|  |  |  |  |  |  |  |  |  | Rotation (both sides) | M: 3.0 (2.4); CGH: 3.5 (2.0) | 2.7 (1.6) |
| Luedtke, 2018^73^ | ICHD-III | 74 M (72.5%) | 28 (27.5%) | 39.2 (12.8) | 43.3 (12.2) | 14.5 (4.5) | 44.60% | EMG, mean amplitude change stress-rest, % | Trapezius | 22.1 (32.6) | 4.75 (10.3) |
| Luedtke, 2018b^74^ | ICHD-III | 179 M (71.03) | 73 (28.97%) | 40 (15) | 40 (13) | 12 (8) | 179 (100%) | PAIVM, %, manual palpation | C0-C2 local pain | 88.8 | 49.3 |
|  |  |  |  |  |  |  |  |  | C0-C2 referred pain | 46.6 | 15.5 |
| Luedtke, 2018c^18^ | ICHD-III | 138 M (65.4%) | 73 (34.6%) | 39 (12.2) | 40 (13.4) | 12.4 (8.4) | 138 (100%) | HFP, cm, CROM device | Sitting position | 17.9 (1.7) | 18.1 (2.0) |
|  |  |  |  |  |  |  |  | ROM, º, goniometer | All movements | 335.7 (46) | 356.6 (46.4) |
|  |  |  |  |  |  |  |  | FRT, º, goniometer | Both sides | 89 (13.9) | 98 (17.3) |
|  |  |  |  |  |  |  |  | Latent trigger points, no, manual palpation | 17 different points | 11.2 (6.8) | 5.1 (4.8) |
|  |  |  |  |  |  |  |  | Upper Cx Q: restriction, pain, or both, manual examination |  | 0.5 (0-4) | 0 (0-3) |
|  |  |  |  |  |  |  |  | PPIVMs, manual examination | C0-C2 | 0 (0-7) | 0 (0-12) |
|  |  |  |  |  |  |  |  | PAIVMs, manual examination | C0-C3 | 6 (1-12) | 6 (1-11) |
|  |  |  |  |  |  |  |  | Thoracic spine screening, manual examination | Clinical signs (pain or hypomobility), 0-6 | 2.2 (0-9) | 1 (0-6) |
|  |  |  |  |  |  |  |  | CCFT, mmHg hold for 10 seconds | Stabilizer | 26 (22-28) | 28 (22-28) |
|  |  |  |  |  |  |  |  | Muscle strength: weak, moderate or strong | Upper trapezius, prone, 0-2 | 0 (0-2) | 0 (0-2) |
|  |  |  |  |  |  |  |  | Reproduction and resolution, %, manual examination | Sustained unilateral PAIVM C0-C3 | 45.7 | 15 |
| Luedtke, 2018d^75^ | ICHD-III | 28 M (57.14%) | 21 (42.86%) | 34.4 (11.9) | 39.8 (13.6) | 9.4 (9.1) | 28 (100%) | TPD, mm, callipers | Neck, left | 27.4 (8.2) | 24.9 (7.1) |
|  |  |  |  |  |  |  |  |  | Neck, right | 26.3 (7.3) | 21.7 (6.2) |
|  |  |  |  |  |  |  |  |  | Hand | 8.3 (4.0) | 6.4 (2.2) |
| Maranhao, 2015^56^ | ICHD-II | 30 M (50%) | 30 (50%) | 39.5 (NR) | 38.9 (NR) | NR | NR | HIT, -/+, (%) | Passive unpredictable head rotation | 19/11 (63.3/36.7) | 23/7 (76.7/23.3) |
|  |  |  |  |  |  |  |  | HST, -/+ (%) | Presence of nystagmus after rotation | 28/2 (93.3/6.7) | 30/0 (100/0) |
|  |  |  |  |  |  |  |  | DVA, -/+ (%) | ETDRS chart | 17/13 (56.7/43.3) | 22/8 (73.3/26.7) |
|  |  |  |  |  |  |  |  | SVV, -/+ (%) | Redirect a line drawn in a bucket | 26/4 (86.7/13.3) | 26/4 (86.7/13.3) |
|  |  |  |  |  |  |  |  | mCTISB, -/+ (%) | Standing in a firm surface | 24/6 (80/20) | 27/3 (90/10) |
|  |  |  |  |  |  |  |  | RTT, -/+ (%) | Standing, 30 s eyes closed, 30 s opened | 19/11 (63.3/36.7) | 27/3 (90/10) |
|  |  |  |  |  |  |  |  | PPTest, -/+ (%) | Closed eyes, to perceive starting position | 24/6 (80/20) | 26/4 (86.7/13.3) |
|  |  |  |  |  |  |  |  | FT, -/+ (%) | Closed eyes, stepping in place | 13/17 (43.3/56.7) | 18/12 (60/40) |
|  |  |  |  |  |  |  |  | TUG, -/+ (%) | Time to complete the path | 29/1 (96.7/3.3) | 30/0 (100/0) |
|  |  |  |  |  |  |  |  | 5TSST, -/+ (%) | Stands up and sit down 5 times | 26/4 (86.7/13.3) | 30/0 (100/0) |
|  |  |  |  |  |  |  |  | Forward reaching test, -/+ (%) | Bending the trunk | 30/0 (100/0) | 30/0 (100/0) |
|  |  |  |  |  |  |  |  | DGI, -/+ (%) | Eight item task | 26/4 (86.7/13.3) | 29/1 (96.7/3.3) |
|  |  |  |  |  |  |  |  | PT, -/+ (%) | Standing, pulled backward by shoulders | 26/4 (86.7/13.3) | 30/0 (100/0) |
| Marcus, 1999^35^ | ICHD-I | 24 M (25%) | 24 (25%) | NR | NR | NR | NR | Posture, Kendall criteria, % | Normal posture | 16.7 | 54.2 |
|  |  |  |  |  |  |  |  |  | Mild abnormalities | 66.7 | 45.8 |
|  |  |  |  |  |  |  |  |  | Moderate to severe abnormalities | 16.7 | 0 |
|  |  |  |  |  |  |  |  | Latent trigger points, %, manual palpation | Different muscles | 79.2 | 50 |
| Milanov, 2003^36^ | ICHD-I | 15 M (23.33%) | 32 (53.33%) | 40 (12.8) | 34 (6.6) | 4 (2.2) | NR | Trigeminocervical reflex, EMG | Latency, ms | 47.6 (6.8) | 49.7 (7.6) |
|  |  |  |  |  |  |  |  |  | Duration, ms | 70.5 (29.8) | 73.7 (24.6) |
|  |  |  |  |  |  |  |  |  | Amplitude, mV | 0.3 (0.2) | 0.44 (0.2) |
|  |  |  |  |  |  |  |  |  | Area mV*ms | 5.2 (3) | 4.9 (4.4) |
| Nardone, 2008^57^ | ICHD-II | 30 M (54.5% | 15 (27.2%) | 37.2 (20.1) | 38.4 (16.4) | 3 (1) | NR | Trigeminocervical reflex, EMG | Latency, ms | 20.3 (1.4) | 19.0 (1.3) |
|  |  |  |  |  |  |  |  |  | Amplitude, mV | 0.53 (0.38) | 1.12 (0.26) |
| Ogince, 2007^17^ | M: ICHD-I; CGH: CHISG | 12 M (20.6%); 23 CGH (39.7%) | 23 (39.7%) | M: 37 (NR); CGH 46 (NR) | 40 (NR) | NR | NR | FRT, º, goniometer |  | M: 39 (6.9); CGH: 20 (11) | 39 (6.5) |
| Oliveira-Souza, 2019^76^ | ICHD-III | 55 M (64.7%) | 30 (35.3%) | 35.3 (14.6) | 32 (11.26) | 15.4 (1.9) | 55 (100%) | ROM, º, goniometer | Extension | 62 (18.5) | 72 (8.1) |
|  |  |  |  |  |  |  |  |  | Flexion | 52 (15.5) | 58 (9.5) |
|  |  |  |  |  |  |  |  |  | Rotation, both sides | 57.8 (9.1) | 62 (8.7) |
|  |  |  |  |  |  |  |  |  | Lateral flexion, both sides | 34.5 (9) | 37 (6.7) |
|  |  |  |  |  |  |  |  |  | FRT, both sides | 29.5 (9.2) | 41 (7.4) |
| Palacios-Ceña, 2016^77^ | ICHD-III | 103 M (67.7%) | 52 (33.3%) | 40.5 (16.5) | 40 (12.6) | 11.9 (1.7) | 103 (100%) | PPT, kg/cm2, algometer | Temporalis muscle | 1.8 (1.1) | 2.8 (0.5) |
|  |  |  |  |  |  |  |  |  | C5/C6 | 1.7 (1.0) | 2.7 (1.4) |
|  |  |  |  |  |  |  |  |  | Second metacarpal | 2.4 (1.5) | 3.5 (1.7) |
|  |  |  |  |  |  |  |  |  | Tibialis anterior muscle | 3.3 (1.7) | 5 (1.4) |
| Park, 2017^68^ | ICHD-II | 20 CGH (50%) | 20 (50%) | 33 (5.5) | 31 (6.1) | NR | NR | Tone, Hz, MyotonPro | Suboccipital muscle | 15.6 (2.9) | 13.3 (3.3) |
|  |  |  |  |  |  |  |  |  | Trapezius muscle | 16.1 (2.3) | 19.4 (2.5) |
|  |  |  |  |  |  |  |  | Stiffness, N/m, MyotonPro | Suboccipital muscle | 323.9 (35.9) | 260.1 (32.1) |
|  |  |  |  |  |  |  |  |  | Trapezius muscle | 355.5 (56.7) | 293.4 (33.3) |
|  |  |  |  |  |  |  |  | Elasticity, log decrement, MyotonPro | Suboccipital muscle | 1.3 (0.2) | 1.2 (0.1) |
|  |  |  |  |  |  |  |  |  | Trapezius muscle | 0.9 (0.1) | 1.1 (0.1) |
| Petersen, 2015^88^ | CHISG | 12 CGH (54.45%) | 10 (45.5%) | 29.41 (9.76) | 29.08 (8.25) | NR | NR | FRT, º, goniometer | Active, both sides | 27.9 (9.3) | 28.5 (7.2) |
|  |  |  |  |  |  |  |  |  | Passive, both sides | 42 (15.7) | 43.4 (8.5) |
| Pires, 2017^69^ | ICHD-II | 20 M (33.33%) | 20 (33.33%) | 34.7 (8.1) | 34.0 (9.1) | NR | NR | PPT, kg/cm2, algometer | Temporalis muscle, 4 points | 0.9 (0.3) | 1.7 (0.4) |
|  |  |  |  |  |  |  |  |  | Anterior point | 0.9 (0.2) | 1.7 (0.4) |
|  |  |  |  |  |  |  |  |  | Medium point 1 | 0.9 (0.3) | 1.7 (0.4) |
|  |  |  |  |  |  |  |  |  | Medium point 2 | 0.8 (0.3) | 1.7 (0.5) |
|  |  |  |  |  |  |  |  |  | Posterior point | 0.8 (0.3) | 1.8 (0.5) |
|  |  |  |  |  |  |  |  |  | SCM, 6 points | 0.7 (0.3) | 1.3 (0.4) |
|  |  |  |  |  |  |  |  |  | Sternal 1 | 0.6 (0.3) | 1.2 (0.5) |
|  |  |  |  |  |  |  |  |  | Sternal 2 | 0.6 (0.2) | 1.1 (0.4) |
|  |  |  |  |  |  |  |  |  | Sternal 3 | 0.8 (0.3) | 1.3 (0.4) |
|  |  |  |  |  |  |  |  |  | Clavicular 1 | 0.6 (0.2) | 1.3 (0.5) |
|  |  |  |  |  |  |  |  |  | Clavicular 2 | 0.6 (0.2) | 1.2 (0.4) |
|  |  |  |  |  |  |  |  |  | Clavicular 3 | 0.8 (0.3) | 1.4 (0.4) |
|  |  |  |  |  |  |  |  |  | Suboccipital muscles, 2 points | 0.8 (0.3) | 1.7 (0.4) |
|  |  |  |  |  |  |  |  |  | Point 1 | 0.9 (0.4) | 1.7 (0.5) |
|  |  |  |  |  |  |  |  |  | Point 2 | 0.8 (0.3) | 1.6 (0.3) |
|  |  |  |  |  |  |  |  |  | Upper trapezius muscle, 1 point | 0.7 (0.3) | 2.1 (1.4) |
| Sandrini, 1994^37^ | ICHD-I | 21 M (20.6%) | 37 (36.3%) | 39 (6.3) | NR | NR | NR | PPT, kg/cm2, algometer | Frontalis muscle | 3.5 (0.89) | 3.8 (0.96) |
|  |  |  |  |  |  |  |  |  | Trapezius muscle | 2.9 (0.79) | 3.5 (1.3) |
|  |  |  |  |  |  |  |  | EMG at rest | Trapezius muscle | 295 (130) | 216 (81) |
|  |  |  |  |  |  |  |  | EMG during contraction | Trapezius muscle | 889 (758) | 1024 (613) |
|  |  |  |  |  |  |  |  | EMG at rest | Frontalis muscle | 204 (92) | 209 (60) |
|  |  |  |  |  |  |  |  | EMG during contraction | Frontalis muscle | 726 (383) | 1037 (425) |
| Tali, 2014^70^ | ICHD-II | 20 M (50%) | 20 (50%) | 24.95 (1.8) | 25.65 (1.4) | 6.6 (5.9) | NR | ROM, º, goniometer | Flexion | 55.7 (13.6) | 53.1 (11.4) |
|  |  |  |  |  |  |  |  |  | Extension | 86.3 (11.2) | 89.4 (18.1) |
|  |  |  |  |  |  |  |  |  | Rotation, both sides | 70 (9.7) | 70.7 (7.9) |
|  |  |  |  |  |  |  |  |  | Lateral flexion, both sides | 51.4 (9.1) | 48.9 (6.6) |
|  |  |  |  |  |  |  |  | Posture, CVA, º, photograph | Sitting position | 51 (7.3) | 53.2 (4.4) |
|  |  |  |  |  |  |  |  | Segmental stiffness, %, manual examination | C0-C1 | 20 | 0 |
|  |  |  |  |  |  |  |  |  | C1-C2 | 50 | 5 |
|  |  |  |  |  |  |  |  |  | C2-C3 | 40 | 20 |
|  |  |  |  |  |  |  |  |  | C3-C4 | 55 | 25 |
|  |  |  |  |  |  |  |  |  | C4-C5 | 25 | 45 |
|  |  |  |  |  |  |  |  | Latent trigger points, % | Trapezius muscle | 32.5 | 47.5 |
| Vuralli, 2016^78^ | ICHD-III | 15 M (50%) | 15 (50%) | 35.2 (10.7) | 35.4 (11.8) | 17.4 (2.3) | 15 (100%) | PPT, kg/cm2, algometer | Temporalis muscle | 1.7 (0.5) | 3.9 (0.8) |
|  |  |  |  |  |  |  |  |  | GON | 1.5 (0.4) | 4.4 (0.7) |
| Wanderley, 2015^61^ | ICHD-II | 21 M (43.8%) | 11 (22.9%) | 23.1 (2.2) | 21.6 (0.9) | 4.1 (2.1) | NR | EMG, RMS | SCM, beginning of contraction | 0.5 (0.3) | 0.6 (0.1) |
|  |  |  |  |  |  |  |  |  | SCM, end of contraction | 0.4 (0.1) | 0.5 (0.1) |
| Watson, 2012^15^ | ICHD-II | 20 M (41.7%) | 14 (29.2%) | 35.3 (NR) | 32.8 (NR) | NR | NR | Tenderness, NRS, manual examination | C0-C1 | 6.7 (0.4) | 6 (0.5) |
|  |  |  |  |  |  |  |  |  | C2-C3 | 7.3 (0.4) | 6.1 (0.5) |
|  |  |  |  |  |  |  |  | Headache reproduction, % | C0-C1 and C2-C3 assessment | 95 | 57 |
| Zito, 2006^14^ | M: ICHD-II; CGH: CHISG | 25 M (32.47%); CGH: 27 (35.05%) | 25 (32.47%) | M: 22.9 (3.5); CGH: 25.3 (3.9) | 22.9 (3.5) | Not reported | Not reported | Posture, CVA, º, photograph | Standing position | M: 53.3 (3.9); CGH: 51.1 (5.8) | 50.3 (4.6) |
|  |  |  |  |  |  |  |  | Posture, eye-tragion angle, º, photograph | Standing position | M: 15.9 (4.9); CGH: 15.2 (5.0) | 13.0 (5.7) |
|  |  |  |  |  |  |  |  | PPT, kg/cm2, algometer | C2 | M: 3.0 (1.1); CGH: 3.3 (1.1) | 3.2 (1.0) |
|  |  |  |  |  |  |  |  |  | GON | M: 4.5 (2.0); CGH: 4.3 (1.4) | 4.9 (1.6) |
|  |  |  |  |  |  |  |  |  | C2-C3 | M: 3.3 (1.2); CGH: 3.4 (1.0) | 3.6 (1.1) |
|  |  |  |  |  |  |  |  |  | C4 | M: 3.8 (1.2); CGH: 3.9 (1.1) | 4.5 (1.2) |
|  |  |  |  |  |  |  |  | Cervical kinesthesia, º, laser | Flexion | M: 4.3 (1.7); CGH: 4.3 (2.0) | 4.1 (1.9) |
|  |  |  |  |  |  |  |  |  | Extension | M: 5.3 (2.7); CGH: 5.4 (2.7) | 5.2 (2.1) |
|  |  |  |  |  |  |  |  |  | Rotation, both sides | M: 5.1 (2.7); CGH: 5.5 (2.6) | 6.2 (2.5) |
|  |  |  |  |  |  |  |  | CROM, goniometer | Upper cervical flexion/extension | M: 41.1 (15.9); CGH: 39.2 (14.2) | 42.8 (13.4) |
|  |  |  |  |  |  |  |  |  | Flex + Ext | M: 137.4 (36.3); CGH: 136.7 (26.8) | 138.8 (16.9) |
|  |  |  |  |  |  |  |  |  | Rotation (both sides) | M: 152.2 (32.5); CGH: 151.2 (30.8) | 157.5 (22.5) |
|  |  |  |  |  |  |  |  |  | Lateral flexion (both sides) | M: 83.7 (20.1); CGH: 82.3 (19.7) | 84.4 (20.4) |
|  |  |  |  |  |  |  |  | FRT, º, goniometer | Sum of sides | 83.2 (20.7); CGH: 81.6 (34.5) | 91.9 (20.6) |
|  |  |  |  |  |  |  |  | PAIVMs, stiffness % and VAS, manual examination | C0-C1, both sides | M: 28 (2); CGH: 76 (5) | 24 (2) |
|  |  |  |  |  |  |  |  |  | C1-C2, both sides | M: 28 (2); CGH: 78 (5) | 20 (2) |
|  |  |  |  |  |  |  |  |  | C2-C3, both sides | M: 20 (2); CGH: 52 (3) | 8 (1) |
|  |  |  |  |  |  |  |  |  | C3-C4, both sides | M: 16 (1); CGH: 22 (2) | 2 (1) |
|  |  |  |  |  |  |  |  | Muscle soreness, %, manual palpation | Upper trapezius | M: 6.3; CGH: 16.7 | 6.2 |
|  |  |  |  |  |  |  |  |  | Levator scapulae | M: 7.1; CGH: 18.9 | 4.2 |
|  |  |  |  |  |  |  |  |  | Scalene | M: 2.6; CGH: 17.6 | 8.1 |
|  |  |  |  |  |  |  |  |  | Suboccipital | M: 15.8; CGH: 25.2 | 12.1 |
|  |  |  |  |  |  |  |  |  | Pectoralis major | M: 2.2; CGH: 8.2 | 3.1 |
|  |  |  |  |  |  |  |  |  | Pectoralis muscle | M: 0; CGH: 5.1 | 2 |
|  |  |  |  |  |  |  |  | Mechanosensitivity of neural muscle, %, manual palpation | ULTT and SLRT + CCF | M: 0; CGH 7.4 | 0 |
|  |  |  |  |  |  |  |  | CCFT, EMG, % value at 20mmHg | SCM, 22 mmHG | M: 112.7 (56.13); CGH: 128.23 (105.1) | 108.3 (50.42) |
|  |  |  |  |  |  |  |  |  | SCM, 30 mmHG | M: 209.6 (115.37); CGH: 276.4 (296.9) | 206.1 (180.1) |
| Zwart, 1997^38^ | M: ICHD-I; CGH: CHISG | M: 28 (19.86%); CGH: 28 (19.86%) | 51 (36.17%) | M: 39.5 (10.4); CGH: 42 (8.8) | 42.8 (16.7) | NR | NR | ROM, º, goniometer | Flex + Ext | M: 133 (19.9); CGH: 107 (17.6) | 129 (17.9) |
|  |  |  |  |  |  |  |  |  | Rotation, both sides | M: 174 (16.6); CGH: 146 (23.4) | 170 (22) |
|  |  |  |  |  |  |  |  |  | Lateral flexion, both sides | M: 91 (14.2); CGH: 86 (12.9) | 94 (17.9) |

AE: Absolut Error; CCFT: Cranio-Cervical Flexion Test; CE: Constant Error; CGH: Cervicogenic Headache; CHISG: Cervicogenic Headache Internation Study Group; CROM: Cervical Range Of Motion; CVA: Cranio-vertebral angle; DGI: Dynamic Gait Index; DVA: Dynamic Visual Acuity; FT: Fukuda Test; FRT: Flexion-Rotation Test ; FRTest: Forward Reaching Test; HRT: Head Repositioning Test; HFP: Head Forward Posture; HIT: Head Impulse Test; HST: Head Shaking Test H-ECNHP: Head-to-Eye Closed Neutral Head Position; H-TT; Head-to-Target Test; ICHD: International Classification of Headache Disorders; JPE: joint position error; LOS: Limits of Stability; M: Migraine; MIT: Minimal Ice Test; N: Newtons; mCTSIB: Modified Clinical Test of Sensory Interaction and Balance; NR: Not Reported; PAIVM: Passive Accesory Intervertebral Movement; PPIVM: Passive Physiological Intervertebral Movement;PPT: Pressure Pain Threshold; PPTest: Past Pointing Test; PT: Pull Test

RMS: Root Mean Sqaure; ; RTT: Romberg Tandem Test; SCM: Sternocleidomastoid; S: Seconds; SLRT: Straight Leg Raise Test; SVV: Subjective Visual Vertical; TCR: Trigemino-Cervical Reflex; TUG: Timed Up and Go; ULTT: Upper Limb Tension Test; VAS: Visual Analogue Scale; ; 5TSST: 5 Times Sit To Stand Test;
