# Supplementary material for: Differentiating migraine, cervicogenic headache and asymptomatic individuals based on physical examination findings: a systematic review and meta-analysis
Source: BMC Musculoskelet Disord. 2021 Sep 3;22:755. doi: 10.1186/s12891-021-04595-w (PMC8417979; doi:10.1186/s12891-021-04595-w)
Supplement: Supplementary file 3 — Additional file 3. Risk of bias assessment with a modified version of Downs and Black Scale. [file 12891_2021_4595_MOESM3_ESM.docx]

**Appitional File 3.** Risk of bias assessment with a modified version of Down’s and Black Scale

|  | Reporting (partly or rather completely fulfilled: 1; not fulfilled: 0) | | | | | | External validity | | | Internal validity | | | | Power | Sum | Quality index (%) |
| --- | --- | --- | --- | --- | --- | --- | --- | --- | --- | --- | --- | --- | --- | --- | --- | --- |
| Study | 1 | 2 | 3 | 5 | 6 | 7 | 10 | 11 | 12 | 15 | 18 | 20 | 25 | 27 |  |  |
| Akdal, 2009^39^ | 1 | 1 | 0 | 2 | 1 | 1 | 1 | 0 | 0 | 0 | 1 | 1 | 1 | 0 | 9 | 60 |
| Assapun, 2017^81^ | 1 | 1 | 1 | 2 | 1 | 1 | 1 | 1 | 0 | 1 | 1 | 1 | 1 | 1 | 14 | 93.3 |
| Baron, 2017^62^ | 1 | 1 | 1 | 2 | 1 | 1 | 1 | 0 | 1 | 1 | 1 | 1 | 1 | 1 | 14 | 93.3 |
| Benatto, 2019^63^ | 1 | 1 | 1 | 2 | 1 | 1 | 1 | 1 | 0 | 0 | 1 | 1 | 1 | 1 | 13 | 86.6 |
| Bevilaqua-Grossi, 2009^54^ | 1 | 1 | 1 | 2 | 1 | 1 | 1 | 0 | 0 | 1 | 1 | 1 | 1 | 1 | 13 | 86.6 |
| Bevilaqua-Grossi, 2011^55^ | 1 | 1 | 1 | 2 | 1 | 1 | 1 | 1 | 0 | 1 | 1 | 1 | 0 | 1 | 13 | 86.6 |
| Bovim, 1992^33^ | 0 | 1 | 0 | 1 | 1 | 1 | 1 | 0 | 0 | 0 | 1 | 1 | 0 | 0 | 7 | 46.6 |
| Calandre, 2006^40^ | 1 | 1 | 1 | 1 | 1 | 1 | 1 | 1 | 1 | 0 | 1 | 1 | 0 | 0 | 7 | 46.6 |
| Carnevalli, 2018^64^ | 1 | 1 | 1 | 2 | 1 | 1 | 0 | 1 | 0 | 0 | 1 | 1 | 1 | 1 | 12 | 80 |
| Carvalho, 2013^41^ | 1 | 1 | 1 | 1 | 1 | 1 | 1 | 1 | 1 | 1 | 1 | 1 | 1 | 1 | 14 | 93.3 |
| Carvalho, 2016^65^ | 1 | 1 | 1 | 2 | 1 | 1 | 1 | 1 | 0 | 1 | 1 | 1 | 1 | 1 | 12 | 80 |
| Chen, 2018^82^ | 1 | 1 | 1 | 2 | 1 | 1 | 1 | 1 | 0 | 0 | 1 | 1 | 1 | 1 | 10 | 66.6 |
| Cooke, 2007^79^ | 1 | 1 | 1 | 2 | 1 | 1 | 1 | 1 | 1 | 1 | 1 | 0 | 0 | 1 | 13 | 86.6 |
| Dehertogh, 2008^83^ | 1 | 1 | 1 | 2 | 1 | 1 | 1 | 1 | 1 | 0 | 1 | 1 | 1 | 0 | 13 | 86.6 |
| Drummond, 1987^80^ | 0 | 1 | 0 | 1 | 1 | 0 | 0 | 1 | 0 | 0 | 1 | 1 | 0 | 0 | 6 | 40 |
| Dugailly, 2017^66^ | 1 | 1 | 1 | 0 | 1 | 1 | 1 | 1 | 0 | 0 | 1 | 1 | 1 | 0 | 10 | 60 |
| Dumas, 2001^34^ | 1 | 1 | 1 | 2 | 1 | 1 | 1 | 1 | 1 | 0 | 1 | 1 | 0 | 0 | 10 | 66.6 |
| Engstrom, 2013^42^ | 1 | 1 | 1 | 2 | 1 | 1 | 1 | 1 | 1 | 1 | 1 | 0 | 1 | 0 | 13 | 86.6 |
| Engstrom, 2013b^43^ | 1 | 1 | 1 | 2 | 1 | 1 | 1 | 1 | 1 | 1 | 1 | 0 | 1 | 1 | 13 | 86.6 |
| Engstrom, 2014^44^ | 1 | 0 | 1 | 2 | 1 | 1 | 0 | 1 | 1 | 1 | 1 | 0 | 1 | 0 | 12 | 80 |
| Fernandez-de-las-Peñas, 2006^45^ | 1 | 1 | 0 | 2 | 1 | 1 | 1 | 0 | 0 | 1 | 1 | 1 | 1 | 0 | 11 | 73.3 |
| Fernandez-de-las-Peñas, 2006b^46^ | 1 | 1 | 0 | 2 | 1 | 1 | 0 | 0 | 0 | 1 | 1 | 1 | 1 | 0 | 10 | 66.6 |
| Fernandez-de-las-Peñas, 2008^47^ | 1 | 1 | 0 | 2 | 1 | 1 | 1 | 0 | 0 | 1 | 1 | 1 | 1 | 0 | 11 | 73.3 |
| Fernandez-de-las-Peñas, 2009^48^ | 1 | 1 | 1 | 0 | 1 | 1 | 0 | 0 | 0 | 1 | 1 | 1 | 0 | 0 | 8 | 53.3 |
| Fernandez-de-las-Peñas, 2009b^49^ | 1 | 1 | 1 | 0 | 1 | 1 | 0 | 0 | 0 | 1 | 1 | 1 | 1 | 0 | 9 | 60 |
| Fernandez-de-las-Peñas, 2010^50^ | 1 | 1 | 1 | 1 | 1 | 0 | 1 | 1 | 0 | 1 | 1 | 1 | 1 | 1 | 12 | 80 |
| Ferracini, 2016^51^ | 1 | 1 | 1 | 2 | 1 | 1 | 1 | 1 | 1 | 1 | 1 | 1 | 1 | 0 | 14 | 93.3 |
| Ferracini, 2017^67^ | 1 | 1 | 1 | 2 | 1 | 1 | 1 | 1 | 1 | 1 | 1 | 1 | 1 | 0 | 14 | 93.3 |
| Ferreira, 2014^52^ | 1 | 1 | 1 | 2 | 1 | 1 | 0 | 1 | 0 | 1 | 1 | 1 | 1 | 1 | 13 | 86.6 |
| Florencio, 2015^53^ | 1 | 1 | 1 | 2 | 1 | 1 | 1 | 1 | 1 | 1 | 1 | 1 | 0 | 1 | 14 | 93.3 |
| Florencio, 2015b^68^ | 1 | 1 | 1 | 2 | 1 | 1 | 1 | 1 | 1 | 1 | 1 | 1 | 1 | 1 | 15 | 100 |
| Florencio, 2016^64^ | 1 | 1 | 1 | 2 | 1 | 1 | 1 | 1 | 0 | 1 | 1 | 0 | 1 | 0 | 12 | 80 |
| Florencio, 2018^70^ | 1 | 1 | 1 | 2 | 1 | 1 | 1 | 1 | 1 | 1 | 1 | 1 | 1 | 0 | 14 | 93.3 |
| Florencio, 2019^71^ | 1 | 1 | 1 | 2 | 1 | 1 | 1 | 1 | 1 | 1 | 1 | 1 | 1 | 1 | 15 | 100 |
| Hall, 2004^84^ | 1 | 1 | 1 | 1 | 1 | 1 | 1 | 1 | 1 | 1 | 1 | 0 | 1 | 0 | 12 | 80 |
| Hall, 2010b^86^ | 1 | 1 | 1 | 2 | 1 | 1 | 1 | 1 | 1 | 1 | 1 | 1 | 1 | 1 | 15 | 100 |
| Horwitz, 2015^72^ | 1 | 1 | 1 | 2 | 1 | 1 | 1 | 1 | 0 | 1 | 1 | 1 | 0 | 1 | 13 | 86.6 |
| Huber, 2012^87^ | 1 | 1 | 1 | 2 | 1 | 1 | 1 | 1 | 1 | 1 | 1 | 1 | 1 | 0 | 14 | 93.3 |
| Luedtke, 2018^73^ | 1 | 1 | 1 | 2 | 1 | 1 | 1 | 1 | 1 | 1 | 1 | 0 | 1 | 1 | 14 | 93.3 |
| Luedtke, 2018b^74^ | 1 | 1 | 1 | 2 | 1 | 1 | 1 | 1 | 0 | 1 | 1 | 0 | 1 | 1 | 13 | 86.6 |
| Luedtke, 2018c^18^ | 1 | 1 | 1 | 2 | 1 | 1 | 1 | 1 | 0 | 1 | 1 | 1 | 1 | 1 | 14 | 93.3 |
| Luedtke, 2018d^75^ | 1 | 1 | 1 | 2 | 1 | 1 | 1 | 1 | 0 | 1 | 1 | 1 | 1 | 1 | 14 | 93.3 |
| Maranhao, 2015^56^ | 1 | 0 | 1 | 0 | 1 | 0 | 1 | 0 | 0 | 1 | 0 | 0 | 0 | 0 | 5 | 33.3 |
| Marcus, 1999^35^ | 1 | 1 | 0 | 2 | 1 | 1 | 1 | 1 | 0 | 0 | 0 | 1 | 1 | 0 | 10 | 66.6 |
| Milanov, 2003^36^ | 1 | 1 | 1 | 1 | 1 | 0 | 0 | 0 | 0 | 0 | 1 | 0 | 0 | 0 | 6 | 40 |
| Nardone, 2008^57^ | 1 | 1 | 1 | 1 | 1 | 1 | 0 | 0 | 0 | 1 | 1 | 1 | 0 | 0 | 9 | 60 |
| Oliveira-Souza, 2019^76^ | 1 | 1 | 1 | 2 | 1 | 1 | 0 | 1 | 1 | 1 | 1 | 1 | 1 | 1 | 14 | 93.3 |
| Palacios-Ceña, 2016^77^ | 1 | 1 | 1 | 2 | 1 | 1 | 1 | 0 | 1 | 1 | 1 | 1 | 1 | 1 | 14 | 93.3 |
| Park, 2017^68^ | 1 | 1 | 0 | 1 | 1 | 1 | 0 | 0 | 0 | 0 | 1 | 0 | 0 | 0 | 6 | 40 |
| Petersen, 2015^88^ | 1 | 1 | 1 | 2 | 1 | 1 | 1 | 0 | 0 | 1 | 1 | 1 | 1 | 1 | 13 | 86.6 |
| Pires, 2017^69^ | 1 | 1 | 1 | 2 | 1 | 1 | 1 | 1 | 0 | 0 | 1 | 1 | 1 | 1 | 13 | 86.6 |
| Sandrini, 1994^37^ | 1 | 1 | 0 | 1 | 1 | 1 | 0 | 0 | 0 | 1 | 1 | 0 | 1 | 0 | 8 | 53.3 |
| Tali, 2014^70^ | 1 | 1 | 1 | 2 | 1 | 1 | 1 | 0 | 0 | 1 | 1 | 1 | 1 | 0 | 12 | 80 |
| Vuralli, 2016^78^ | 1 | 1 | 1 | 2 | 1 | 1 | 1 | 1 | 1 | 1 | 1 | 0 | 1 | 1 | 14 | 93.3 |
| Wanderley, 2015^61^ | 1 | 1 | 1 | 2 | 1 | 1 | 1 | 0 | 1 | 1 | 1 | 0 | 0 | 0 | 11 | 73.3 |
| Watson, 2012^15^ | 1 | 1 | 1 | 1 | 1 | 1 | 1 | 1 | 0 | 0 | 1 | 1 | 0 | 0 | 10 | 66.6 |
| Zwart, 1997^38^ | 1 | 1 | 0 | 2 | 1 | 1 | 1 | 0 | 0 | 1 | 1 | 1 | 0 | 0 | 10 | 66.6 |

1: hypothesis/aim/objective of the study clearly described (yes=1; no=0)

2: main outcomes to be measured clearly described in the Introduction or Methods section (yes=1; no=0)

3. characteristics of the patients included in the study clearly described (yes=1;no=0)

5: distributions of principal confounders in each group of participants to be compared clearly described (yes=2; partially yes=1; no=0)

6: main findings of the study clearly described (yes=1; no=0)

7: study provided estimates of the random variability in the data for the main outcomes (yes=1; no=0)

10: actual probablity values reported (yes=1; no=0)

11: people asked to participate in the study representative of the entire population from which they were recruited (recruitement method and place (yes=1; no=0)

12: people prepared to participate representative of the entire population from which they were recruited (yes=1; no=0)

15: attempt made to mask particpants measurinf the main outcomes of the intervention (yes=1; no=0)

18: statistical tests used to assess the main outcomes appropiate (yes=1; no=0)

20: main outcome measures used accurate (reliable) (yes=1; no=0)

25: adequate adjustement for confounding in the analyses from which the main findings were drawn (yes=1; no=0)

27: study had sufficient power to detect a clinically important effect simple size calculation or n>50 per group (yes=1; no=0)

Quality Index (QI) was calculated as the percentage of items present in each study. A QI<75% indicates moderate or high risk of bias. QI>75% indicates low risk of bias
