# Supplementary material for: Differentiating migraine, cervicogenic headache and asymptomatic individuals based on physical examination findings: a systematic review and meta-analysis
Source: BMC Musculoskelet Disord. 2021 Sep 3;22:755. doi: 10.1186/s12891-021-04595-w (PMC8417979; doi:10.1186/s12891-021-04595-w)
Supplement: Supplementary file 4 — Additional file 4. Quality assessment with QUADAS-II. [file 12891_2021_4595_MOESM4_ESM.docx]

**Additional file 4.** Quality assessment of diagnostic accuracy studies with QUADAS-II

|  | Risk of Bias | | | | Applicability Concerns | | |
| --- | --- | --- | --- | --- | --- | --- | --- |
| Study | Patient selection | Index test | Reference standard | Flow and Timing | Patient selection | Index test | Reference standard |
| Hall, 2008 | :( | :) | :( | :) | :) | :) | :( |
| Hall, 2010 | :( | :) | :( | :) | :) | :) | :( |
| Jull, 2007 | :( | :) | :( | ? | :) | :) | :( |
| Ogince, 2007 | :( | :) | :( | ? | :( | :) | :( |
| Zito, 2006 | :( | :( | :( | :) | :( | :) | :( |

:) = low risk; :( = high risk; ¿ = unclear risk

**Patient Selection**

- Risk of bias: Could the selection of patients have introduced bias?

Question 1: Was a consecutive or random sample of patients entolled?

Question 2: Was a case-control design avoided?

Question 3: Did the study avoid inappropiate exclusions?

- Applicablity: Are there concerns that the included patients and setting do not match the rview question?

**Index Test**

- Risk of bias: Could the conduct or interpretation of the index test have introduced bias?

Question 1: Were the index test results interpreted without knowledge of the results of the reference standard?

Question 2: If a threshold was used, was it prespecified?

- Applicability: Are there concerns that the index test, its conduct, or its interpretation differ from the review question?

**Reference Standard**

- Could the reference standard, its conduct, or its interpretation have introduced bias?

Question 1: Is the reference standard likely to correctly classify the target condition?

Were the reference standard results interpreted without knowledge of the results of the index test?

Question 2: Were the reference standard results interpreted without knowledge of the results of the index test?

- Applicability: Are there concerns that the target condition as defined by the reference standard does not match the question?

**Flow and Timing**

- Risk of bias: Could the patient flow have introduced bias?

Question 1: Was there an appropiate interval between the index test and reference standard?

Question 2: Did all patients receive the same reference standard?

Question 3: Were all patients included in the analysis?
