# Supplementary material for: Differentiating migraine, cervicogenic headache and asymptomatic individuals based on physical examination findings: a systematic review and meta-analysis
Source: BMC Musculoskelet Disord. 2021 Sep 3;22:755. doi: 10.1186/s12891-021-04595-w (PMC8417979; doi:10.1186/s12891-021-04595-w)
Supplement: Supplementary file 5 — Additional file 5. Outcomes not included in the meta-analysis comparing cervicogenic headache versus migraine. [file 12891_2021_4595_MOESM5_ESM.docx]

**Additional file 5.** Outcomes not included in the meta-analysis comparing cervicogenic headache versus migraine

| Test evaluated | Study | Test procedure or location | Significant differences |
| --- | --- | --- | --- |
| FRT | Zito, 2006^14^ | Sum of both sides, º | No difference between groups |
| PPT, kg/cm2 | Bovim, 1992^33^ | 22 points in whole head | Reduced in CGH |
|  | Zito, 2006^14^ | C2 nerve root | No difference between groups |
|  |  | GON | No difference between groups |
|  |  | C2-C3 Zygapophyseal joint | No difference between groups |
|  |  | C4 transverse process | No difference between groups |
| PAIVM, % | Zito, 2006^14^ | C0-C1, both sides | Increased VAS and stiffness % in CGH |
|  |  | C1-C2, both sides | Increased VAS and stiffness % in CGH |
|  |  | C2-C3, both sides | Increased VAS and stiffness % in CGH |
|  |  | C3-C4, both sides | Increased VAS and stiffness % in CGH |
|  | Jull, 2007^19^ | C0-C1 | Increased % of SCJ in CGH |
|  |  | C1-C2 | Increased % of SCJ in CGH |
|  |  | C2-C3 | Increased % of SCJ in CGH |
|  |  | C3-C4 | Increased % of SCJ in CGH |
|  |  | C4-C5 | No difference between groups |
|  |  | C5-C6 | No difference between groups |
|  |  | C6-C7 | No difference between groups |
|  |  | C7-T1 | No difference between groups |
|  | Dumas, 2001^34^ | Cervical spine, 3 point scale | Increased % of dysfunction in CGH |
| PPIVMs, % | Dumas, 2001^34^ | Cervical spine, 3 point scale | No difference between groups |
| Skin roll test | Dumas, 2001^34^ | VAS 0-100 | No difference between groups |
| CCFT | Jull, 2007^19^ | SCM, 22mmHg, EMG, RMS | Poorer performance in CGH |
|  |  | SCM, 30mmHg, EMG, RMS | Poorer performance in CGH |
|  | Zito, 2006^14^ | SCM, 22mmHg, % EMG value | No difference between groups |
|  |  | SCM, 30mmHg, % EMG value | No difference between groups |
| Endurance | Dumas, 2001^34^ | Deep neck flexors, seconds | No difference between groups |
| Muscle soreness, %, manual palpation | Zito, 2006^14^ | Upper trapezius | No difference between groups |
|  |  | Levator scapulae | No difference between groups |
|  |  | Scalene | No difference between groups |
|  |  | Suboccipital | No difference between groups |
|  |  | Pectoralis major | No difference between groups |
|  |  | Pectoralis muscle | No difference between groups |
| Posture | Zito, 2006^14^ | CVA, º, standing position | No difference between groups |
|  | Zito, 2006^14^ | Eye-tragion angle, º, standing position | No difference between groups |
| Mechanosensitivity of neural tissue, % | Zito, 2006^14^ | ULTT and SLRT + CCF | More prevalent in CGH |
| JPE, º | Dumas, 2001^34^ | Rotation, both sides 30º | No difference between groups |
|  |  | Rotation, both sides 50º | No difference between groups |
|  |  | LF both sides, 20º | No difference between groups |

CCF: Cranio-Cervical Flexion; CVA: cranio-vertebral angle;EMG: Electromyography; FRT: Flexion-Rotation Test; GON: Greater Occipital Nerve; JPE: Joint Position Error; LF: Lateral Flexion; PAIVM: Passive Accesory Intervertebral Movements; PPT: Pressure Pain Threshold; SCM: Sternocleidomastoid; SCJ: Symptomatic Cervical Joint

SLRT: Straight Leg Raising Test; ULTT: Upper Limb Tension Test; VAS: Visual Analogue Scale.
