# Supplementary material for: Differentiating migraine, cervicogenic headache and asymptomatic individuals based on physical examination findings: a systematic review and meta-analysis
Source: BMC Musculoskelet Disord. 2021 Sep 3;22:755. doi: 10.1186/s12891-021-04595-w (PMC8417979; doi:10.1186/s12891-021-04595-w)
Supplement: Supplementary file 6 — Additional file 6. Outcomes not included in the meta-analysis comparing migraine and asymptomatic individuals. [file 12891_2021_4595_MOESM6_ESM.docx]

**Additional file 6**. Outcomes not included in the meta-analysis comparing migraine and asymptomatic individuals

| Test evaluated | Study | Test procedure or location | Significance |
| --- | --- | --- | --- |
| ROM | Luedtke, 2018c^18^ | All movements, º | Reduced in migraine |
| FRT | Ferracini, 2017^67^ | % of positive tests | More prevalent in migraine |
| JPE, º | Dumas, 2001^34^ | Rotation, both sides 30º | No difference between groups |
|  |  | Rotation, both sides 50º | No difference between groups |
|  |  | LF both sides, 20º | No difference between groups |
| Posture | Ferreira, 2014^52^ | CVA, standing position, º | No difference between groups |
|  |  | CLA, standing position, º | No difference between groups |
|  |  | Thoracic kyphosis, standing position, º | No difference between groups |
|  | Ferracini, 2016^51^ | High cervical angle, º | No difference between groups |
|  |  | Distance C0-C1, cm | No difference between groups |
|  | Horwitz, 2015^72^ | Distance plumb line-mid-neck, cm | No difference between groups |
|  | Luedtke, 2018c^18^ | HFP, cm | No difference between groups |
|  | Marcus, 1999^35^ | Kendall criteria (none, mild, moderate-severe) | Increased abnormalities in migraine |
|  | Zito, 2006^14^ | Eye-traction angle, º | No difference between groups |
| PPT, kg/cm2 | Bevilaquia-Grossi, 2011^55^ | Fp1 (frontal point) | Reduced in migraine |
|  |  | Fp2 (frontal point) | Reduced in migraine |
|  | Barón, 2017^62^ | 10 points | Reduced in migraine |
|  | Sandrini, 1994^37^ | Frontalis muscle | No difference between groups |
|  |  | Temporalis muscle | No difference between groups |
|  | Fernández-de-las-Peñas, 2009^48^ | Supraorbital, radial, median and ulnar nerves | Reduced in migraine |
|  | Fernández-de-las-Peñas, 2009b^49^ | 9 points of temporalis muscle | Reduced in migraine |
|  | Pires, 2017^69^ | Temporalis muscle | Reduced in migraine |
|  |  | SCM | Reduced in migraine |
|  |  | Suboccipital | Reduced in migraine |
|  |  | Upper trapezius | Reduced in migraine |
|  | Palacios-Ceña, 2016^77^ | Temporalis muscle | Reduced in migraine |
|  | Fernández-de-las-Peñas, 2010^50^ | Upper trapezius | Reduced in migraine |
|  |  | Levator scapulae | Reduced in migraine |
|  | Florencio, 2015b^68^ | Scalene | Reduced in migraine |
|  | Zito, 2006^14^ | C2 | No difference between groups |
|  |  | GON | No difference between groups |
|  |  | C2-C3 | No difference between groups |
|  |  | C4 | No difference between groups |
|  | Bovim, 1992^33^ | 22 points in whole head | No difference between groups |
| CCFT | Zito, 2006^14^ | SCM, 22mmHg, % EMG value | No difference between groups |
|  |  | SCM, 30mmHg, % EMG value | Increased in migraine |
|  | Luedtke, 2018c^18^ | Hold for ten seconds, stabilizer | Worse performance in migraine in last stages |
|  | Jull, 2007^19^ | SCM, 22mmHg, EMG, RMS | No difference between groups |
|  |  | SCM, 30mmHg, EMG, rms | Worse performance in migraine |
|  | Benatto, 2019^63^ | 22 mmHg, logarithmic mean | No difference between groups |
|  |  | 30mmHg, logarithmic mean | Worse performance in migraine |
| Latent trigger points | Fernández-de-las-Peñas, 2006^45^ | Suboccipital, % | Increased in migraine |
|  |  | SCM, % | Increased in migraine |
|  |  | Temporalis muscle, % | Increased in migraine |
|  |  | Upper trapezius muscle, % | Increased in migraine |
|  | Calandre, 2006^40^ | Temporalis muscle, % | Increased in migraine |
|  |  | Suboccipital area, % | Increased in migraine |
|  |  | Occipital area, % | Increased in migraine |
|  |  | Trapezius muscle, % | Increased in migraine |
|  | Luedtke, 2018c^18^ | 17 points in craniocervical area | Increased in migraine |
|  | Horwitz, 2015^72^ | Trapezius, % | Increased in migraine |
|  |  | SCM, % | Increased in migraine |
|  | Fernández-de-las-Peñas, 2006b^46^ | Trcohlear region, VAS | Increased in migraine |
| Endurance short neck flexors | Dumas, 2001^34^ | Short neck flexors, seconds | Reduced in migraine |
| Strength, Newton | Florencio 2019^71^ | Flexors | Reduced in migraine |
|  |  | Extensors | Reduced in migraine |
| Muscle length, reduced range, manual examination | Horwitz, 2015^72^ | Trapezius | Reduced in migraine |
|  |  | Levator scapulae | No difference between groups |
|  |  | SCM | Reduced in migraine |
|  |  | Pectoralis minor | No difference between groups |
|  |  | Anterior scalene | No difference between groups |
|  |  | Deep occipital muscles | No difference between groups |
| Stifness | Dugailly, 2017^66^ | Axial rotation, torsionmeter, | No difference between groups |
|  | Tali, 2014^70^ | Manual palpation, C0-C1, % | Increased in migraine |
|  |  | Manual palpation, C1-C2, % | Increased in migraine |
|  |  | Manual palpation, C2-C3, % | No difference between groups |
|  |  | Manual palpation, C3-C4, % | No difference between groups |
|  |  | Manual palpation; C4-C5, % | No difference between groups |
| PAIVM | Zito, 2006^14^ | C0-C1, both sides, % and VAS | No difference between groups |
|  |  | C1-C2, both sides, % and VAS | No difference between groups |
|  |  | C2-C3, both sides, % and VAS | No difference between groups |
|  |  | C3-C4, both sides, % and VAS | No difference between groups |
|  | Luedtke, 2018b^74^ | % C0-C2 local pain | Increased in migraine |
|  |  | % C0-C2 referred pain | Increased in migraine |
|  | Luedtke, 2018c^18^ | C0-C3, manual examination, number | Increased in migraine |
|  | Jull, 2007^19^, %, manual palpation | C0-C1 | Increased in migraine |
|  |  | C1-C2 | Increased in migraine |
|  |  | C2-C3 | Increased in migraine |
|  |  | C3-C4 | Increased in migraine |
|  |  | C4-C5 | No difference between groups |
|  |  | C5-C6 | No difference between groups |
|  |  | C6-C7 | No difference between groups |
|  |  | C7-T1 | No difference between groups |
|  | Dumas, 2001^34^ | Cervical spine, 3 point scale | No difference between groups |
|  | Horwitz, 2015^72^ | C2, VAS | No difference between groups |
|  |  | C3, VAS | No difference between groups |
|  |  | C4, VAS | Increased in migraine |
|  |  | C5, VAS | Increased in migraine |
|  |  | C6, VAS | Increased in migraine |
|  |  | C7, VAS | No difference between groups |
|  | Ferracini, 2017^67^ | C0-C1, % | Increased in migraine |
|  |  | C1-C2, % | Increased in migraine |
| PPIVM | Dumas, 2001^34^ | Cervical spine, 3 point scale | No difference between groups |
| Skin roll test | Dumas, 2001^34^ | Trapezius muscle, VAS | No difference between groups |
|  |  | Mandible, VAS | No difference between groups |
| Headache reproduction | Watson, 2012^15^ | C0-C1 and C2-C3 assessment | Increased in migraine |
|  | Luedtke, 2018c^18^ | Sustained unilateral PAM C0-C3 | Increased in migraine |
| Thoracic spine screening | Luedtke, 2018c^18^ | Clinical signs (pain or hypomovility), 0-6 | Increased in migraine |
| Neural mobility | Horwitz, 2015^72^ | ULTT, % | Increased in migraine |
| Mechanosensitivity of neural tissue | Zito, 2006^14^ | ULTT and SLRT + CCF, %, | No difference between groups |
| Two-point discrimination test, mm | Luedtke, 2018d^75^ | Neck, left | No difference between groups |
|  |  | Neck, right | No difference between groups |
|  |  | Hand | No difference between groups |
| Von Frey Hairs stimulation | Cooke, 2007^79^ | Von Frey Hairs, numbers | Increased in migraine |
| Balance | Carvalho, 2013^41^ | Oscillation area, cm^2^ | Increased in migraine |
|  | Carvalho, 2016^65^ | Sensory Organization Test,cm^2^ | Poorer in migraine |
|  | Maranhao, 2015^56^ | Vestibular bedside tests | Poorer performarnce in migraine |
| EMG | Luedtke, 2018^73^ | Cervical flexors, mean amplitude change stress-rest, % | No difference between groups |
|  | Wanderley, 2015^61^ | Upper trapezius, RMS, beginning and end contraction | No difference between groups |

C0-T1: joints from atlanto-axial joint to cervico-thoracic joint; CCF: Cranio-Cervical Flexion; CLA: Cervical Lordosis Angle; CVA: cranio-vertebral angle; EMG: electromyography; GON: Greater Occipital Nerve; HFP: Head Forward Posture; LF: Lateral Flexion; PPIVM: Passive Physiological Intervertebral Movement; SCM: Sternocleidomastoid; SLRT: Straight Leg Raising Test; ULTT: Upper Limb Tension Test; VAS: Visual Analogue Scale;
