# Supplementary material for: Differentiating migraine, cervicogenic headache and asymptomatic individuals based on physical examination findings: a systematic review and meta-analysis
Source: BMC Musculoskelet Disord. 2021 Sep 3;22:755. doi: 10.1186/s12891-021-04595-w (PMC8417979; doi:10.1186/s12891-021-04595-w)
Supplement: Supplementary file 7 — Additional file 7. Outcomes not included in the meta-analysis comparing cervicogenic headache versus asymptomatic individuals. [file 12891_2021_4595_MOESM7_ESM.docx]

**Additional file 7.** Outcomes not included in the meta-analysis comparing cervicogenic headache versus asymptomatic individuals

| Test evaluated | Study | Test procedure or location | Significance |
| --- | --- | --- | --- |
| FRT, º | Bravo-Petersen, 2015^88^ | Active | No difference between groups |
|  | Hall, 2004^84^ | Asymptomatic side | No difference between groups |
|  | Zito, 2006^14^ | Sum of both sides | No difference between groups |
|  | Ogince, 2007^17^ | Mean of both sides | Increased in CGH |
| JPE, º | Dumas, 2001^34^ | Rotation, both sides 30º | No difference between groups |
|  |  | Rotation, both sides 50º | No difference between groups |
|  |  | LF both sides, 20º | No difference between groups |
|  | Chen, 2018^82^ | Head-to-target test | Poorer performance in CGH |
|  | De Hertogh, 2008^83^ | Head repositioning accuracy, cm | No difference between groups |
| PPT, kg/cm2 | Assapun, 2017^81^ | Temporalis muscle | No difference between groups |
|  | Assapun, 2017^81^ | Tibialis anterior belly | No difference between groups |
|  | Bovim, 1992^33^ | 22 points of whole head | No difference between groups |
|  | Zito, 2006^14^ | C2 nerve root | No difference between groups |
|  |  | C4 transverse process | No difference between groups |
|  |  | GON | No difference between groups |
| Strength | Huber, 2012^87^ | SCM, Lovett's scale | Reduced in CGH |
|  |  | Trapezius, Lovett's scale | Reduced in CGH |
|  |  | Cervical erector spinae, Lovett's scale | Reduced in CGH |
| Thickness | Chen, 2018^82^ | Suboccipitalis, mm | No difference between groups |
| Soreness | Zito, 2006^14^ | Trapezius length | Reduced in CGH |
|  |  | Pectoralis minor length | Reduced in CGH |
|  |  | Levator scapulae length | Reduced in CGH |
|  |  | Scalene length | Reduced in CGH |
|  |  | Suboccipitalis muscle length | Reduced in CGH |
|  |  | Pectoralis muscle length | Reduced in CGH |
| Trigger point |  | Trapezius, prevalence | Increased in CGH |
|  |  | SCM, prevalence | Increased in CGH |
|  |  | Cervical erector spinae, prevalence | Increased in CGH |
| Endurance | Dumas, 2001^34^ | Short neck flexors, seconds | No difference between groups |
| CCFT | Zito, 2006^14^ | SCM, 22mmHg, % EMG value | No difference between groups |
|  |  | SCM, 30mmHg, % EMG value | Increased activity in CGH |
|  | Jull, 2007^19^ | SCM, 22 mmHg, RMS | No difference between groups |
|  |  | SCM, 30 mmHg, RMS | Increased in CGH |
| EMG | Huber, 2012^87^ | Trapezius, at rest | Increased in CGH |
|  |  | Trapezius, maximal contraction | Increased in CGH |
|  |  | SCM, at rest | Increased in CGH |
|  |  | SCM, maximal contraction | Increased in CGH |
| PAIVMS | Zito, 2006^14^ | C0-C1, both sides, % and VAS | Increased SCJ in CGH |
|  |  | C1-C2, both sides, % and VAS | Increased SCJ in CGH |
|  |  | C2-C3, both sides, % and VAS | Increased SCJ in CGH |
|  |  | C3-C4, both sides, % and VAS | Increased SCJ in CGH |
|  | Jull, 2007^19^ | C0-C1, % | Increased SCJ in CGH |
|  |  | C1-C2, % | Increased SCJ in CGH |
|  |  | C2-C3, % | Increased SCJ in CGH |
|  |  | C3-C4, % | Increased SCJ in CGH |
|  |  | C4-C5, % | No difference between groups |
|  |  | C5-C6, % | No difference between groups |
|  |  | C6-C7, % | No difference between groups |
|  |  | C7-T1, % | No difference between groups |
|  | Dumas, 2001^34^ | Cervical spine, 3 point scale | Increased % of dysfunction in CGH |
| PPIVMS | Dumas, 2001^34^ | Cervical spine, 3 point scale | No difference between groups |
| Skin roll test | Dumas, 2001^34^ | Trapezius muscle, VAS | No difference between groups |
| Mechano-sensitivity of neural tissue | Zito, 2006^14^ | ULTT and SLRT + CCF, % | Increased in CGH |
| Posture, º | Zito, 2006^14^ | Eye-traction angle | No difference between groups |
|  |  | CVA, standing | No difference between groups |
|  | Dumas, 2001^34^ | CVA, sitting | No difference between groups |

C0-T1: joints from atlanto-axial joint to cervico-thoracic joint; CCF: Cranio-Cervical Flexion; CD: Cervical Dysfunction;CGH: Cervicogenic Headache; CVA: cranio-vertebral angle; EMG: electromyography; FRT: Flexion-Rotation Test; GON: Greater Occipital Nerve; JPE: Joint Position Error; LF: Lateral Flexion; ; PAIVMS: Passive Accesory Intervertebral Movements; PPIVMS: Passive Physiological Intervertebral Movements; PPT: Pressure Pain Threshold; SCJ: Symptomatic Cervical Joint; SCM: Sternocleidomastoid; SLRT: Straight Leg Raising Test; ULTT: Upper Limb Tension Test; VAS: Visual Analogue Scale;
