# Supplementary material for: Differentiating migraine, cervicogenic headache and asymptomatic individuals based on physical examination findings: a systematic review and meta-analysis
Source: BMC Musculoskelet Disord. 2021 Sep 3;22:755. doi: 10.1186/s12891-021-04595-w (PMC8417979; doi:10.1186/s12891-021-04595-w)

**Additional file 8.** Forest plots for meta-analysis and post-hoc sensitivity analysis concerning cervicogenic headache versus migraine comparison. Studies were excluded from post-hoc sensitivity analysis due to moderate/high risk of bias (Quality Index <75%)

1. Cervical ROM.

A.1. Meta-analysis and pos-hoc sensitivity analysis


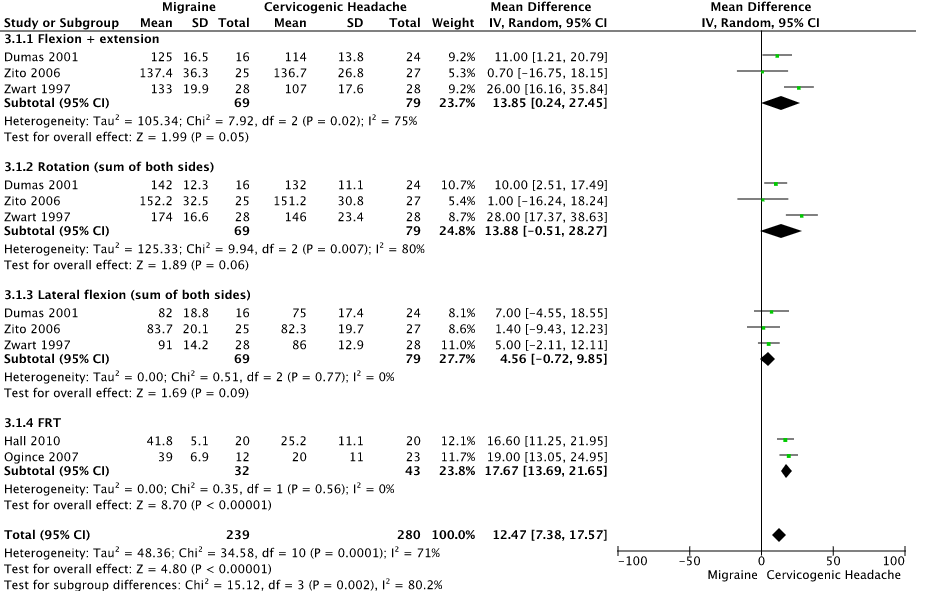


A.2. Pos-hoc sensitivity analysis


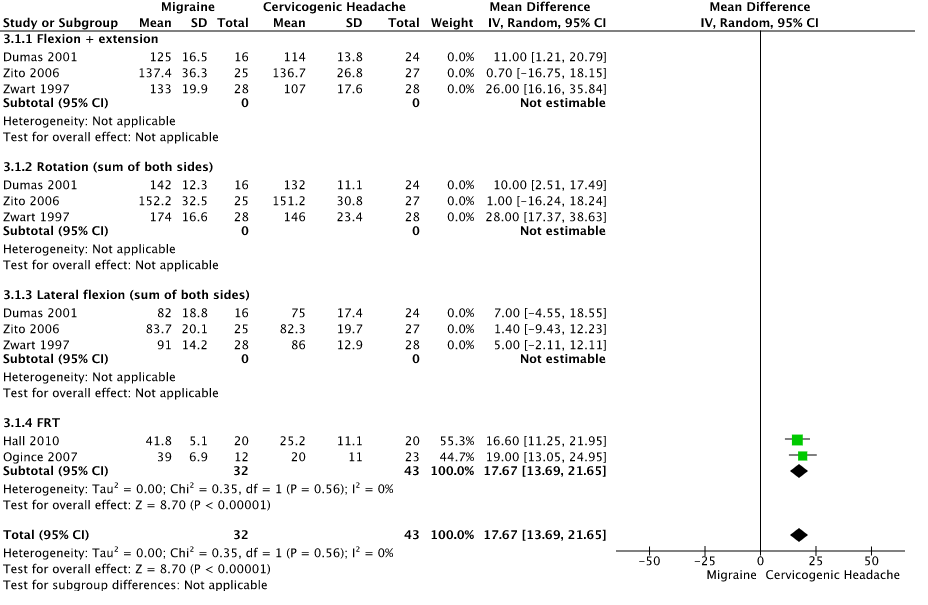


1. Neck strength.

B.1. Meta-analysis


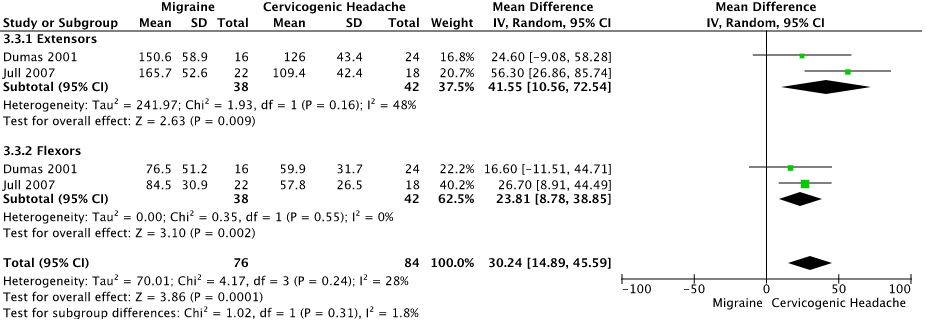

Supplement: Supplementary file 8 — Additional file 8. Forest plots for meta-analysis and post-hoc sensitivity analysis concerning cervicogenic headache versus migraine comparison. [file 12891_2021_4595_MOESM8_ESM.docx]
