# Supplementary material for: Differentiating migraine, cervicogenic headache and asymptomatic individuals based on physical examination findings: a systematic review and meta-analysis
Source: BMC Musculoskelet Disord. 2021 Sep 3;22:755. doi: 10.1186/s12891-021-04595-w (PMC8417979; doi:10.1186/s12891-021-04595-w)

**Additional file 9.** Forest plots for meta-analysis and post-hoc sensitivity analysis concerning migraine and asymptomatic individuals comparison. Studies were excluded from post-hoc sensitivity analysis due to moderate/high risk of bias (Quality Index <75%)

1. Cervical ROM

A.1. Meta-analysis


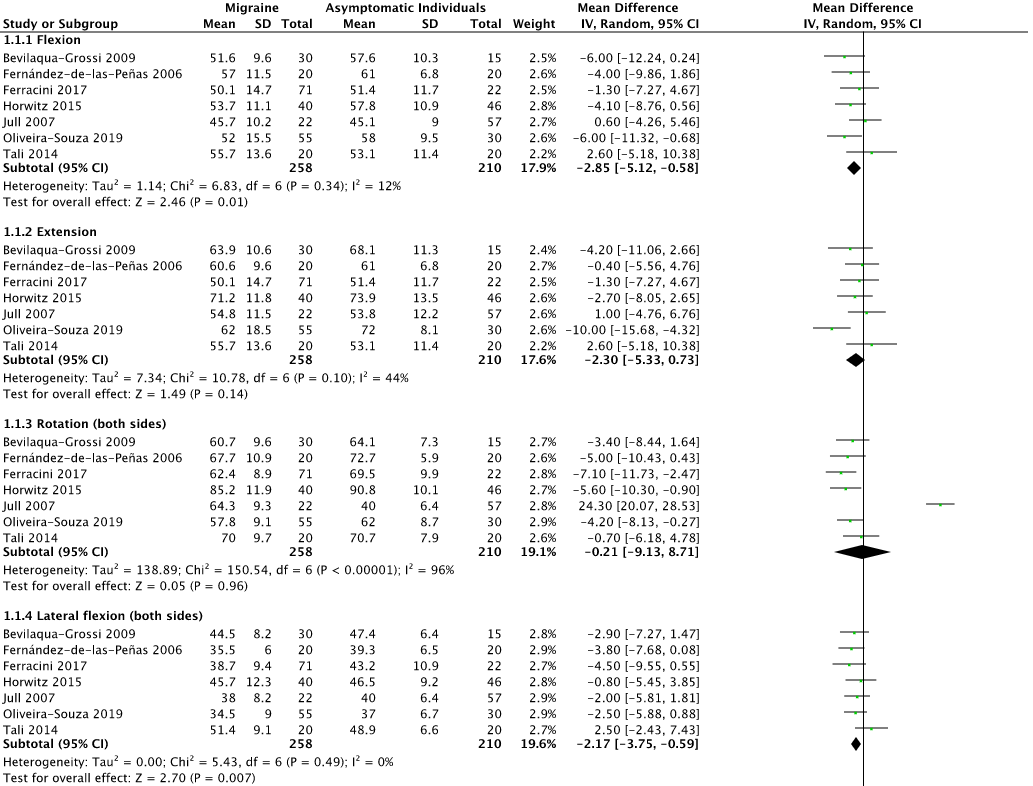


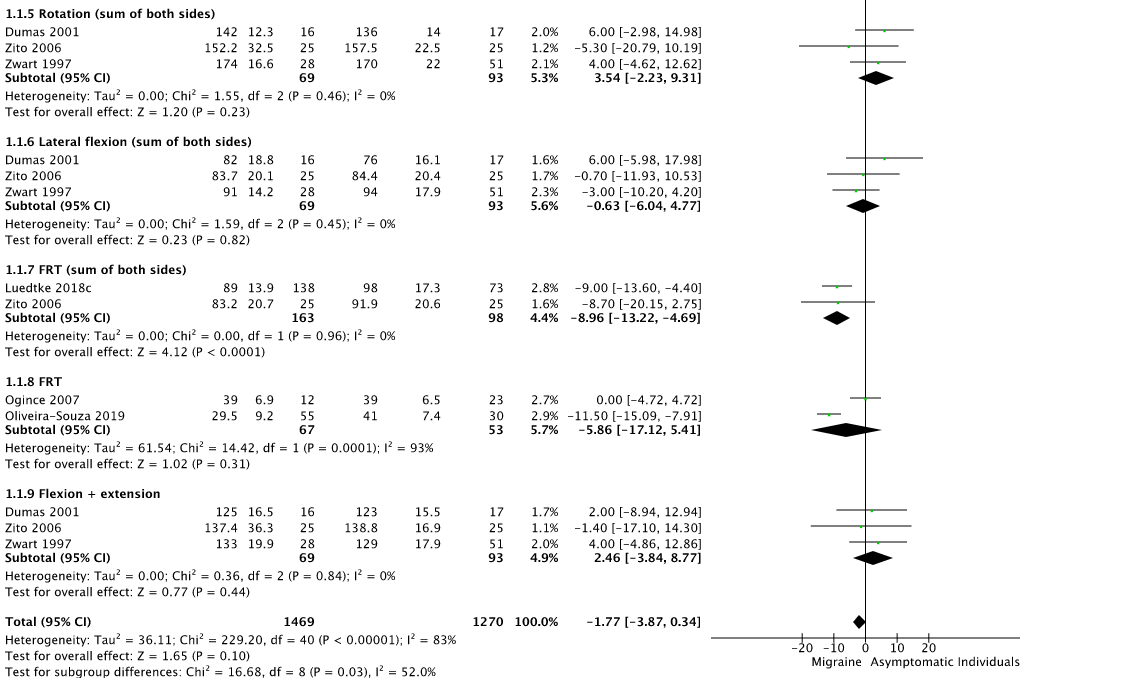


A.2. Pos-hoc sensitivity analysis

**
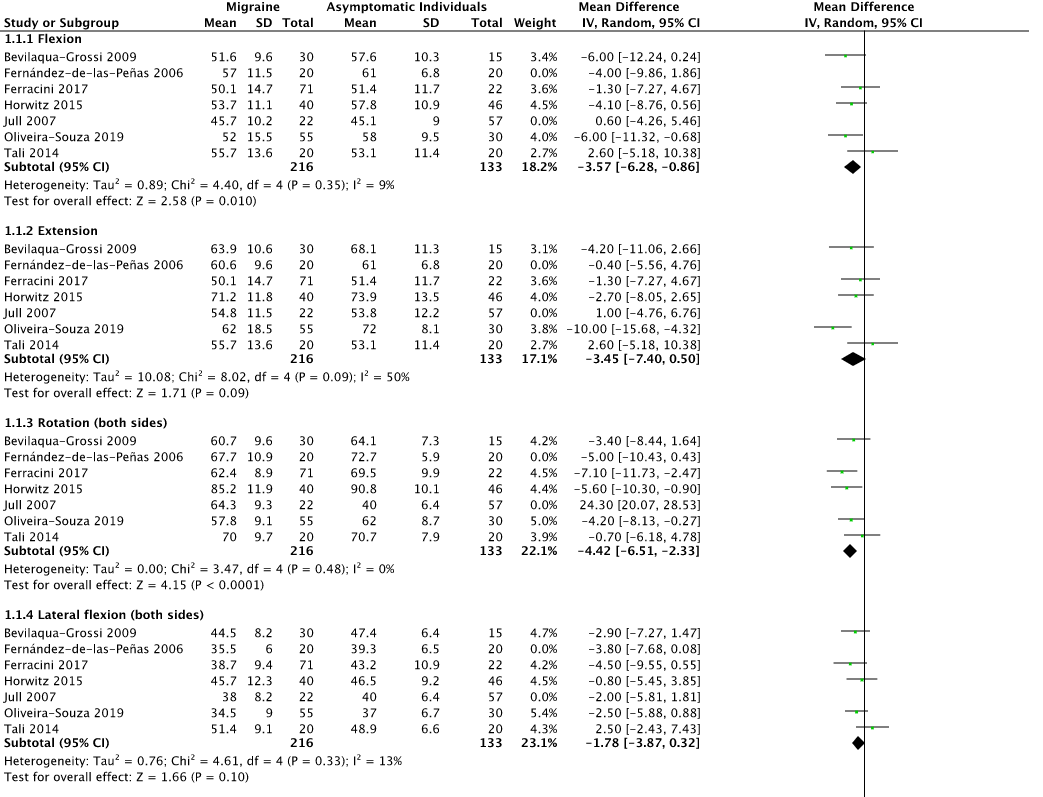
**

**
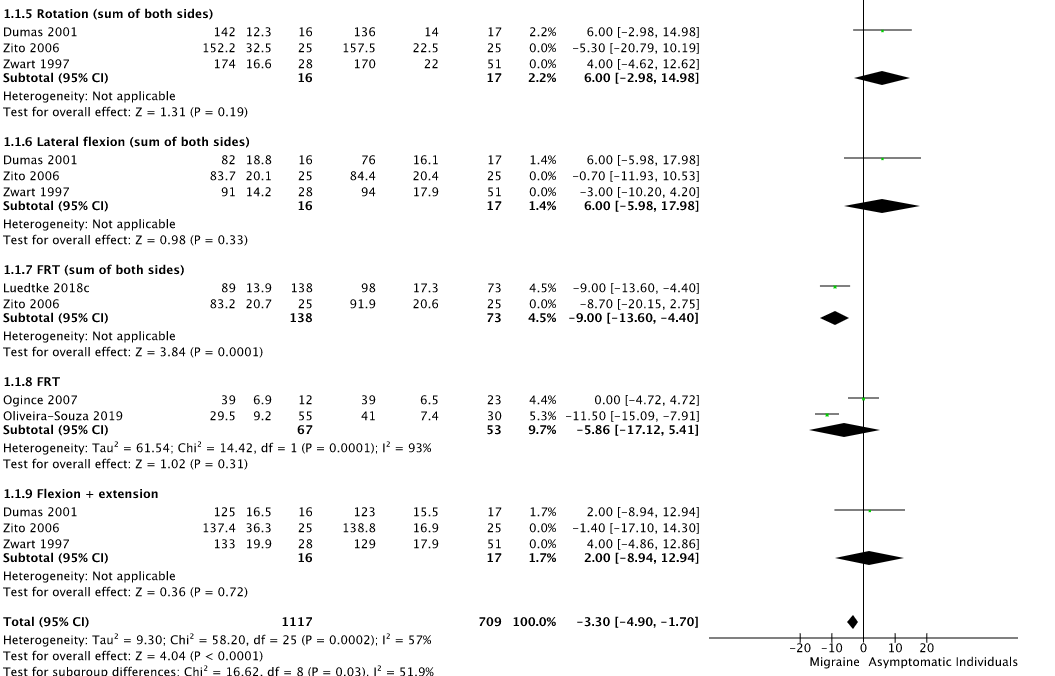
**

1. Posture CLA.

B.1. Meta-analysis and post-hoc sensitivity analysis


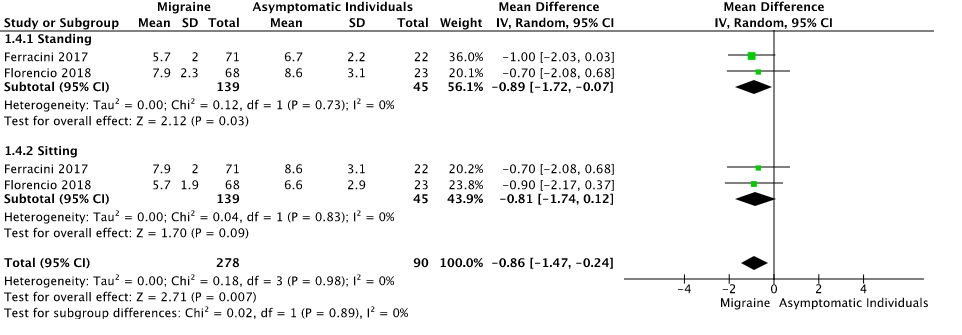


1. Pressure Pain Threshold

C.1. Meta-analysis


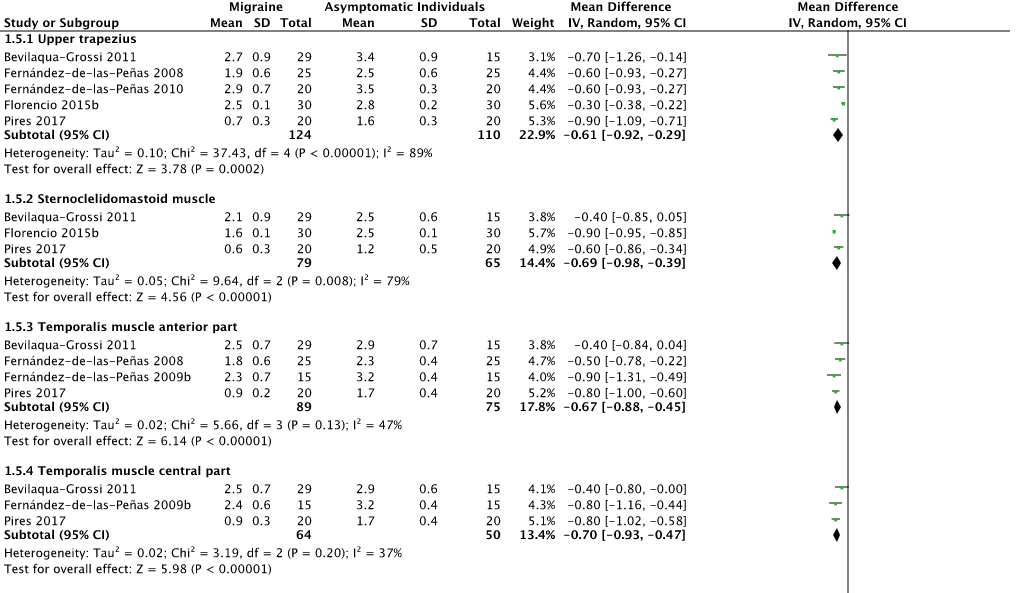


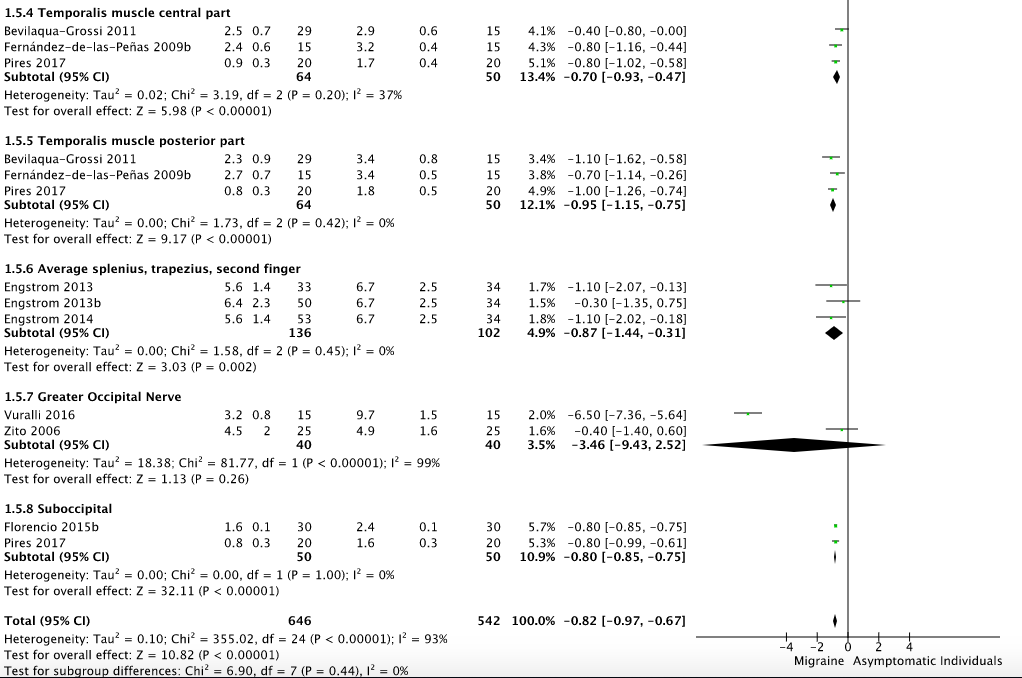


C.2. Post-hoc sensitivity analysis


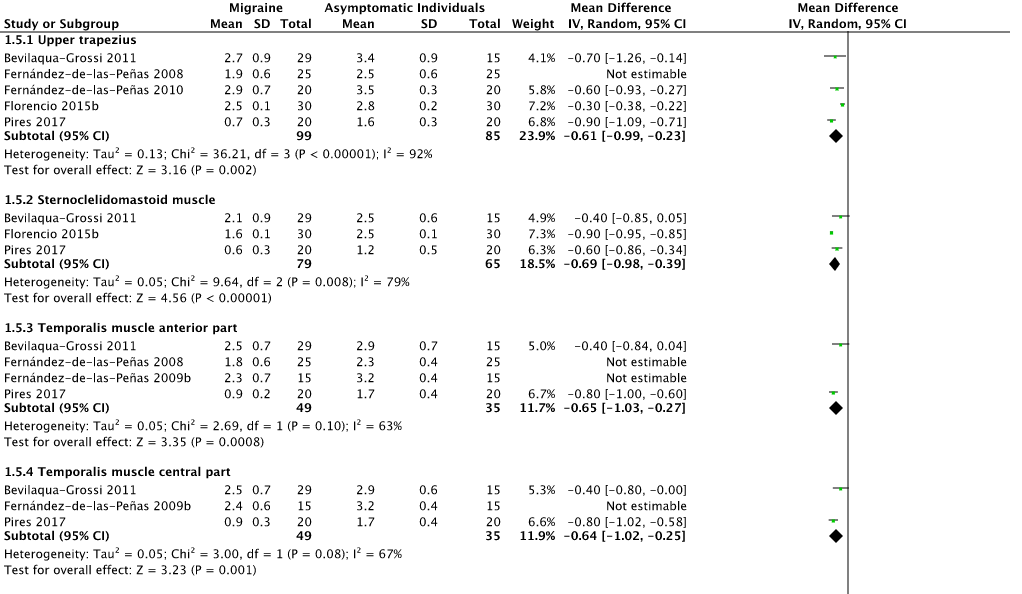


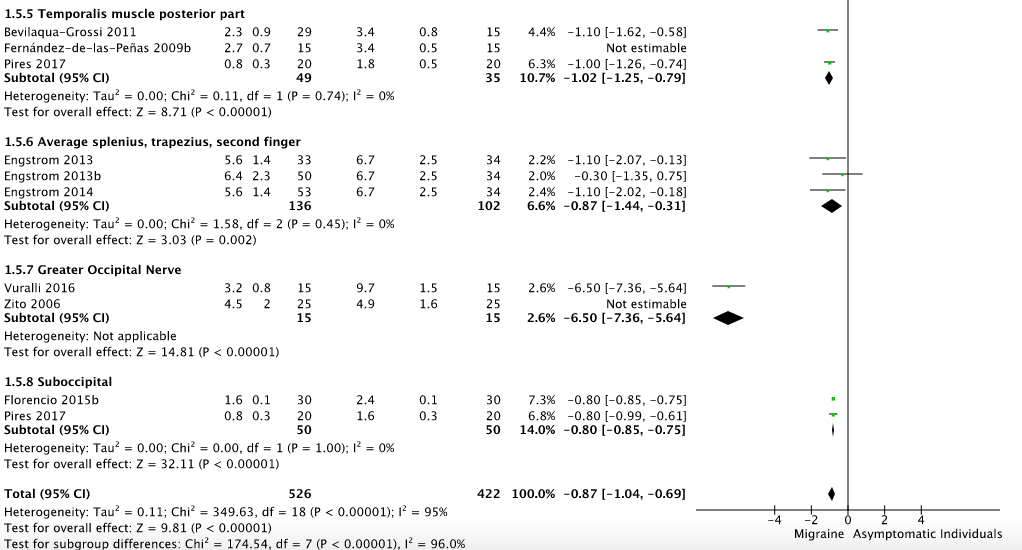


1. Strength

D.1. Meta-analysis


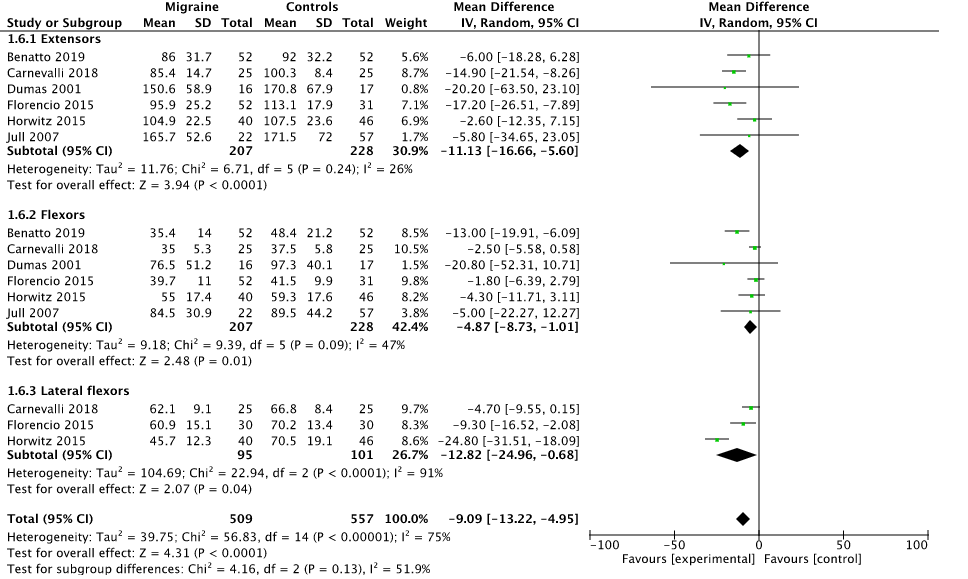


D.2. Post-hoc sensitivity analysis


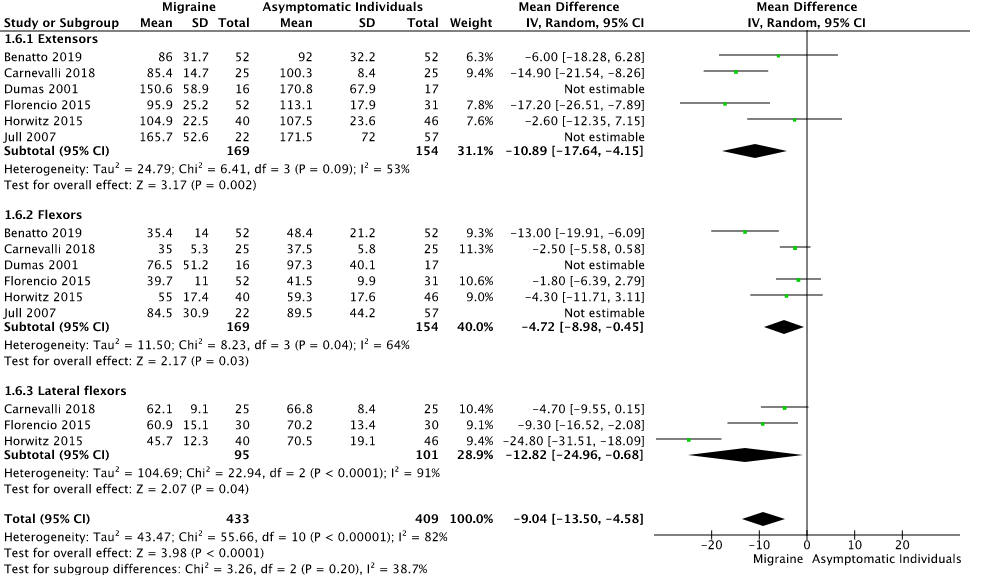


1. CCFT performance at 22 mmHg Rheacer

E.1. Meta-analysis and post-hoc sensivity analysis


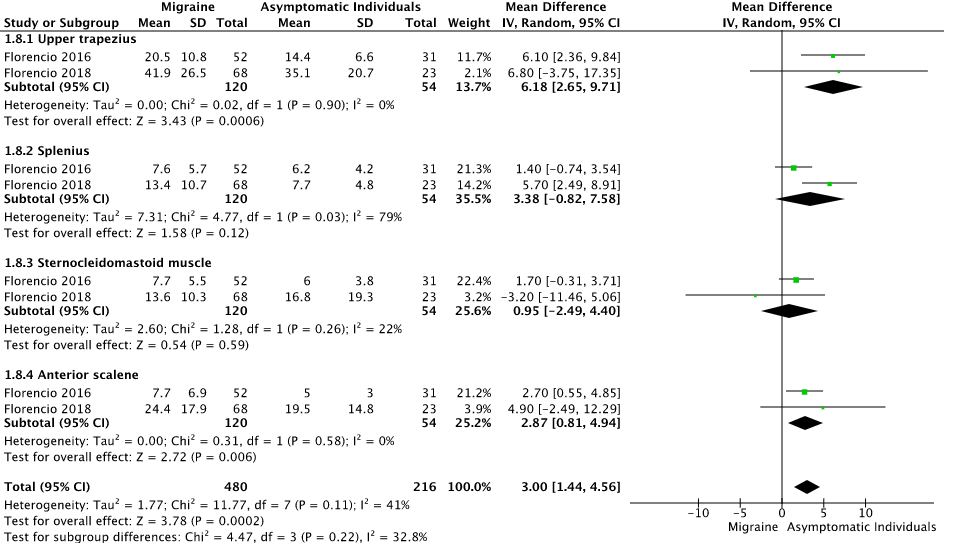


1. CCFT performance at 30 mmHg

F.1. Meta-analysis and post-hoc sensitivity analysis


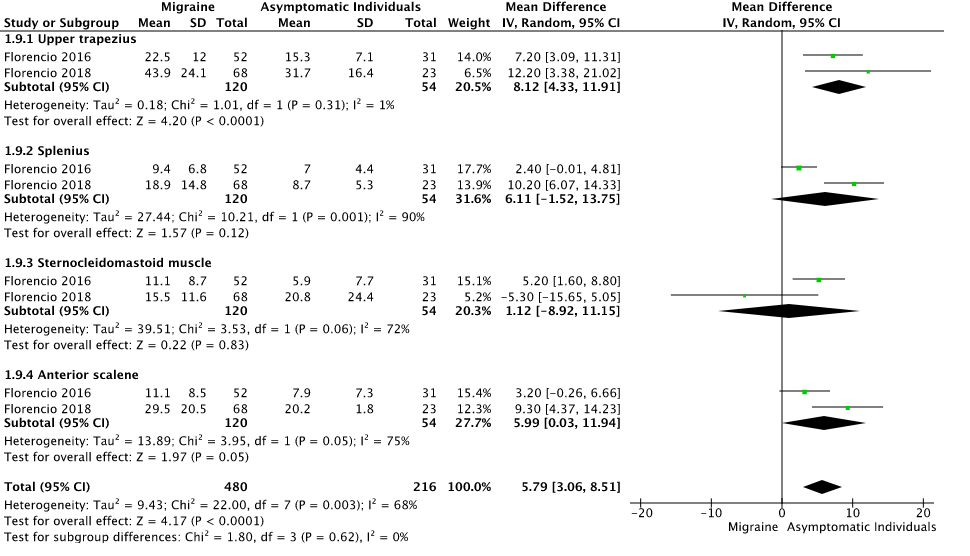

Supplement: Supplementary file 9 — Additional file 9. Forest plots for meta-analysis and post-hoc sensitivity analysis concerning migraine and asymptomatic individuals comparison. [file 12891_2021_4595_MOESM9_ESM.docx]
